# Worldwide prevalence of hepatitis B virus and hepatitis C virus among patients with cirrhosis at country, region, and global levels: a systematic review

**DOI:** 10.1016/S2468-1253(22)00050-4

**Published:** 2022-05-14

**Authors:** Catharina J Alberts, Gary M Clifford, Damien Georges, Francesco Negro, Olufunmilayo A Lesi, Yvan J-F Hutin, Catherine de Martel

**Affiliations:** a Early Detection, Prevention and Infections Branch, International Agency for Research on Cancer (IARC/WHO), Lyon, France; b Divisions of Gastroenterology and Hepatology and of Clinical Pathology, University Hospital, Geneva, Switzerland; c Global HIV, Hepatitis and STIs Programmes, World Health Organization, Geneva, Switzerland; d Department for Universal Health Coverage/Communicable Diseases, World Health Organization Regional Office for the Eastern Mediterranean, Cairo, Egypt

## Abstract

**Background:**

Empirical, updated country-level estimates on the proportion of cirrhosis attributable to viral hepatitis are required. We estimated the prevalence of hepatitis B virus (HBV) and hepatitis C virus (HCV) infection in patients with cirrhosis at country, regional, and global levels as an approximation for the fractions of cirrhosis attributable to viral hepatitis.

**Methods:**

In this systematic review, we searched MEDLINE, Embase, Web of Science, and Scielo between Jan 1, 1993, and Aug 1, 2021. Studies were eligible if they reported on the prevalence of both HBV and HCV infection in representative studies of at least 20 patients with cirrhosis. Studies were excluded if they used first-generation HCV assays or were from a selected population of patients with cirrhosis (eg, patients selected based on specific causes, veterans, injecting drug users). Two authors (CJA and CdM) selected and extracted aggregated data from the selected publications. Data were extracted for study recruitment period, age, sex, and cause of cirrhosis, among others. Data about heavy alcohol consumption and non-alcoholic fatty liver disease (NAFLD) were also extracted when available. Aggregated data from studies from key publications were requested from the authors of the original study if selection of patients was unclear or information on causes was missing. We estimated the country-specific prevalence of causes of cirrhosis by pooling study-level data from the same country using a random-effects model. Subsequently, we estimated the regional (WHO region and UN subregion) and global prevalence by weighting the country-specific prevalence by the number of new liver cancer cases that occurred in 2020, as estimated in GLOBOCAN. The study was registered with PROSPERO, CRD42020149323.

**Findings:**

Our database searches identified 21 338 records, and a further nine records were identified by scanning references of key publications. After excluding duplicates and assessing full-text articles for eligibility, 520 publications from 86 countries or territories (and reporting on 1 376 503 patients with cirrhosis) were included in the systematic review. The prevalence of HBV infection was lower among patients with cirrhosis in Europe, the Americas, and Oceania (UN subregional prevalence ranges 3–14%) than in Africa and Asia (8–61%). HCV infection prevalence was heterogenous, even within regions (12–83%). The combined prevalence of HBV and HCV infection exceeded 50% in most Asian and African regions. Globally, among patients with cirrhosis, 42% had HBV infection and 21% had HCV infection. The contribution of heavy alcohol use was highest in Europe (country range 16–78%), the Americas (17–52%), and Oceania (15–37%) and lowest in Asia (0–41%). Data on NAFLD were limited.

**Interpretation:**

HBV and HCV could account for almost two thirds of the global burden of cirrhosis. With the availability of effective interventions for the prevention or treatment of HBV and HCV, the data presented in this study will help to effectively allocate resources towards viral hepatitis elimination and to design interventions at the country level.

**Funding:**

International Agency for Research on Cancer, World Health Organization.

## Introduction

In 2016, the WHO Global Health Sector Strategy called for hepatitis elimination by 2030 through scaled-up prevention, testing, and treatment.^1^ Elimination of hepatitis as a public health threat was defined as a 90% reduction in incidence and a 65% reduction in mortality, compared with the 2015 baseline. According to the 2017 WHO hepatitis report, chronic infections with hepatitis B virus (HBV) and hepatitis C virus (HCV) were responsible for 96% of the 1·3 million deaths caused by hepatitis viruses worldwide in 2015, of which 720 000 occurred at the cirrhosis stage.^2^ However, exact estimations of the fraction of deaths attributable to these viruses is challenging, because death certificates are often unreliable, and might not capture the underlying cause of death as viral hepatitis.^3^, ^4^

**Research in context**

**Evidence before this study**

Empirical country-level data to estimate mortality from cirrhosis attributable to viral hepatitis are scarce. There are no cirrhosis registries, and mortality data generally do not include the underlying causes of cirrhosis. To address this data gap, WHO recommends applying fractions of cirrhosis attributable to various causes (eg, viral hepatitis) on mortality data from cirrhosis derived from vital registration systems. These attributable fractions can be inferred from published studies on the prevalence of hepatitis B virus (HBV) and hepatitis C virus (HCV) infection among a representative group of patients with cirrhosis. The most recent systematic review reporting on the attributable fraction of viral hepatitis among patients with cirrhosis was published in 2006, and presented data from 29 countries. We systematically searched MEDLINE, Embase, Web of Science, and Scielo databases for original studies, independent of language, published between Jan 1, 1993, and Aug 31, 2021, using various combinations of the keywords “cirrhosis”, “hepatitis B”, “hepatitis C”, and “aetiology”. Studies were eligible if they reported on the prevalence of HBV and HCV in a group of patients with cirrhosis. Studies were excluded if they used first-generation HCV assays or were from a selected population of patients with cirrhosis. Included studies were assessed on methodological aspects of data collection using ten individual quality criteria.

**Added value of this study**

This systematic review estimates the prevalence of HBV and HCV infection and, for cases for which data were available, of heavy alcohol consumption and non-alcoholic fatty liver disease (NAFLD), in patients with cirrhosis. These estimates are based on data from 86 countries or territories representing 87% of the world population. The systematically collected data presented here show the most complete and up-to-date prevalence of HBV and HCV among patients with cirrhosis by country, region, and globally. These prevalence estimates can be used as an approximation of attributable fractions and applied to estimates of incidence and mortality to quantify the proportion of cirrhosis specifically attributable to these causes.

**Implications of all the available evidence**

Improved estimates of mortality from cirrhosis associated with HBV and HCV are necessary to precisely evaluate the burden of viral hepatitis at country, regional, and global levels. Following the WHO call to eliminate hepatitis, these updated data on the prevalence of HBV and HCV will help to assess mortality from cirrhosis associated with HBV and HCV within national contexts to effectively allocate resources to prevent, test for, and treat viral hepatitis. However, continued collection and reporting of empirical data will also be necessary to monitor the effect of efforts towards the 65% mortality reduction from viral hepatitis, one of the criteria used by WHO to define elimination. Other important aetiologies such as alcohol and NAFLD should be considered in this monitoring effort and might become more important as the burden due to viral hepatitis decreases.

In 2016, WHO developed a conceptually simple and pragmatic approach to better estimate mortality from long term sequalae of viral infection.^3^, ^5^ This approach suggested combining data on mortality from cirrhosis and hepatocellular carcinoma derived from vital registration systems with fractions of these sequalae attributable to HBV and HCV—attributable fractions—obtained from representative studies done in patients with cirrhosis or hepatocellular carcinoma. The International Agency for Research on Cancer regularly updates the worldwide prevalence of HBV and HCV infection in published studies of patients with hepatocellular carcinoma and applies these as attributable fractions to hepatocellular carcinoma.^6^, ^7^, ^8^ The Institute for Health Metrics and Evaluation also regularly reports on the cause-specific burden of hepatocellular carcinoma and cirrhosis in the Global Burden of Disease Study (GBD).^9^, ^10^, ^11^ However, there are few up-to-date and systematically collected cause-specific cirrhosis data, especially in countries that could benefit most from focused public health prevention measures. The most recent systematic review on cirrhosis was published in 2006, and identified data from 29 countries.^12^

As well as viral hepatitis, heavy alcohol consumption and non-alcoholic fatty liver disease (NAFLD) are the main other causes of cirrhosis and, in some cases, can also lead to the development of hepatocellular carcinoma.^13^, ^14^ In addition to the already increasing absolute burden of heavy alcohol consumption and NAFLD,^15^ the relative importance of these causes will increase as the fraction of cases due to hepatitis viruses decreases because of hepatitis B immunisation and antiviral treatments.^13^, ^15^

We aimed to provide up-to-date estimates of the prevalence of the main aetiological factors in patients with cirrhosis by country, WHO regions and UN subregions, and worldwide. These included HBV and HCV infection and, in cases for which data were available, heavy alcohol consumption and NAFLD. These prevalence estimates can be used as an approximation of attributable fractions to improve estimates of burden from cirrhosis and viral hepatitis.

## Methods

### Search strategy and selection criteria

We did a systematic review of the literature to estimate the pooled prevalence of HBV and HCV infection among representative studies on patients with cirrhosis. We searched MEDLINE, Embase, Web of Science, and Scielo databases for original studies published between Jan 1, 1993, and Aug 31, 2021, with no language restrictions. The African Journals Online was also searched. The search strategy included various combinations of the following key words: cirrhosis, hepatitis B, hepatitis C, and aetiology (appendix p 2). References from key publications were also scanned.

Studies were eligible for inclusion if they reported on HBV and HCV prevalence; they reported on patients with cirrhosis from a representative, unselected population (eg, from hospitals or health-care centres); they included at least 20 patients; and if patients were diagnosed with compensated cirrhosis, decompensated cirrhosis, or on a waiting list for transplantation or undergoing transplantation. Studies were excluded if they used first-generation HCV assays, if patients were selected on the basis of cause of disease, or if patients were from a selected population of patients with cirrhosis (eg, key populations such as military veterans, prisoners, injecting drug users). Full inclusion and exclusion criteria are provided in the appendix (p 3). When not clearly described in the publication, we relied on the authors’ statement for the diagnosis or cause of cirrhosis. Two authors (CJA and CdM) independently selected studies for inclusion and extracted aggregated data from published reports. Aggregated data from studies from key publications were requested from the authors of the original study if selection of patients was unclear or information on causes was missing. Discrepancies were resolved by mutual agreement between CdM and CJA, after discussion with other co-authors (YJ-FH, FN). Relevant articles not published in English were translated into English for extraction and interpretation of data.

Many studies did not report on HBV and HCV co-infection. For those that did, we reassigned patients with dual infections to only HBV or only HCV according to a ratio equivalent to the overall prevalence of the two viruses in the corresponding study. The study protocol is available online.

### Data extraction

We imported all records to a reference management software (EndNote version X9). After removal of duplicate records according to Bramer and colleagues^16^ we screened the titles and abstracts, and reviewed the full text of all relevant publications following a predefined list of inclusion and exclusion criteria (appendix p 3). Any remaining duplicate records were checked and removed at the extraction stage. When more than one publication reported on the same study population, we included the publication with the most complete or most recent data.

We extracted the following variables whenever available: first author name, publication year, journal name, and country of recruitment. Summary estimates were also extracted for the following variables: stage of cirrhosis (compensated, decompensated, combination of the two, patients on a waiting list for transplantation, and patients undergoing transplantation), study recruitment period, source of population (hospital, outpatient clinic), number of patients within the specified population, age, sex, and cause of cirrhosis (HBV, HCV, dual infection, heavy alcohol consumption, NAFLD).

We created a quality score which encompassed ten individual criteria relevant to our research question that covered various methodological aspects of data. For each item, a study was given one point if a criterion was met, and no points if it was only partially met or not met (appendix p 3). The methodological aspects considered were: (1) aim of the study was to report on the underlying aetiology (yes or no); (2) the study population was an unselected population (eg, all patients, all consecutive patients, randomised patients); (3) inclusion and exclusion criteria were described (yes or no); (4) the definition of cirrhosis was described (yes or no); (5) the numerators added up correctly to the denominator defined by the authors (yes or no); (6) the geographical location of patients was explicitly identified in the main text (yes or no); (7) the study recruitment period was mentioned clearly (yes or no); (8) the data source of the population was described (yes or no); (9) the tests used for detecting HBV and HCV infection were mentioned (yes or no); and (10) collection of the patient data was prospective or cross-sectional (in contrast to retrospective; yes or no).

### Data analysis

HBV and HCV summary prevalence were estimated at the country, regional, and global level. First, we estimated the country-specific prevalence of causes of cirrhosis by pooling study-level data from the same country using a random-effects model with Freeman-Tukey double arcsine transformation with corresponding Wald 95% CIs.^17^ For comparison purposes, we also estimated the pooled prevalence for each country using a fixed-effects model. The fixed-effects model weights were based on within-study variance only, while the random-effects model weights were based on within-study and between-studies variances, using inverse variance method. Larger studies were therefore assigned more weight using the fixed-effects than with the random-effects model.

Subsequently, we estimated the regional (WHO region and UN subregions) and global prevalence by weighting the country-specific prevalence (obtained by random-effects model) by the countries’ respective number of new liver cancer cases that occurred in 2020, as estimated in GLOBOCAN.^18^ We chose this weighting option for three main reasons. First, because liver cancer and cirrhosis share the main risk factors (ie, hepatitis viruses, heavy alcohol use, and NAFLD), an ecological association between their respective burdens is probable at the population level. Second, GLOBOCAN liver cancer data are derived from population-based cancer registries, according to a well-established and publicly available methodology.^18^ Third, this weighting indicator was widely used to estimate regional and global attributable fractions for hepatocellular carcinoma,^8^ allowing comparisons between hepatocellular carcinoma and cirrhosis. We estimated the regional pooled estimates for six WHO regions and for 14 subregions based on UN geographical regions as used in the GLOBOCAN project.^18^ For comparison, we also estimated the regional and global prevalence by weighting individual countries using country population sizes^18^ and various GBD country-specific cirrhosis-related indicators estimated for the year 2017 (number of cirrhosis deaths, number of compensated cirrhosis cases, number of decompensated cirrhosis cases, and total disability-adjusted life-years [DALYs] in 2017).^9^

We did not calculate uncertainty intervals for regional and global estimates. Qualitative factors, including the choice of indicator used to weight country-level pooled estimates, limited the relevance of a numerical estimate of the uncertainty. Instead, we provide a qualitative narrative on the impact of the choice of different burden indicators on the regional-pooled and global-pooled estimates. We also did sensitivity analyses to estimate the effect that the following three scenarios would have on regional and global estimates. First, we excluded studies with a quality score of less than 6. Second, we excluded studies of patients diagnosed with compensated cirrhosis. Third, we excluded studies of patients identified as being on the waiting list or transplant patients.

We analysed data using STATA 17.0 SE and generated maps using R software (version 4.1.0). The study was registered in PROSPERO, CRD42020149323.

### Role of the funding source

The funders of the study had no role in study design, data collection, data analysis, data interpretation or writing of the report.

## Results

**Figure 1:**
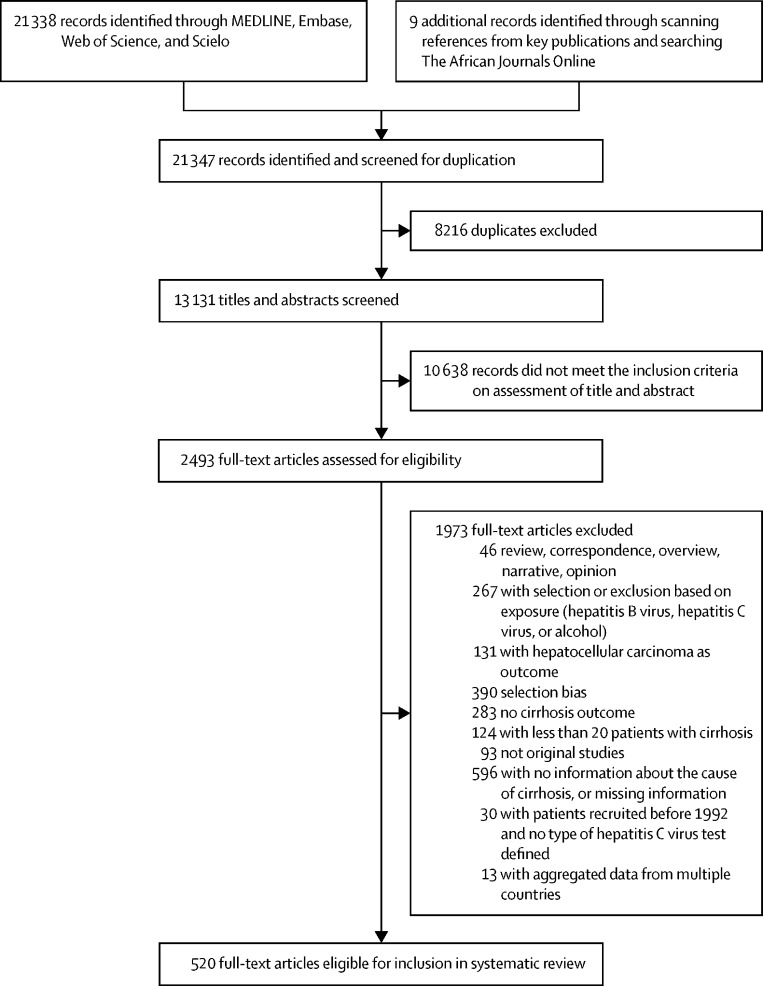
Study selection

21 338 records were identified through searches of MEDLINE, Embase, Web of Science, and Scielo. Nine publications were found by scanning references from key publications and searching The African Journals Online. Of the 13 131 uniquely identified articles during the search period from Jan 1, 1993, to Aug 31, 2021, 2493 publications qualified for full-text review. 1973 records were excluded because they did not meet the inclusion criteria. 520 publications, reporting on 558 individual studies on patients with cirrhosis were included in the systematic review (figure 1). Overall, data on prevalence of HBV and HCV infection were available for 1 376 503 patients with cirrhosis, from 86 countries or territories representing 87% of the world population. The mean age of patients in study populations was lower in countries of South-Central Asia (range 43–56 years) and sub-Saharan Africa (39–52 years) and higher in South America (54–63 years). In 70 (93%) of the 75 countries with data, the proportion of men was equal to or exceeded 50% (table 1).

**Table 1:** Pooled prevalence of HBV or HCV infection among patients with cirrhosis by country or territory*

	**Study years (calendar year)**	**Age (mean)**	**Men (%)**	**Quality score**†**(mean)**	**Number of studies (n)**	**Number of patients (n)**	**HBV prevalence (95% CI)**	**HCV prevalence (95% CI)**
**Oceania**								
Australia	1985–2019	51	73%	7	8	13 044	5% (4–6)	28% (20–35)
New Zealand	1998–2019	50	64%	7	1	272	15% (11–19)	31% (25–36)
**Northern Europe**								
Denmark	2004–13	62	60%	5	2	366	1% (0–3)	4% (2–6)
Estonia	2009–18	55	70%	6	1	98	4% (2–10)	24% (17–34)
Finland	2004–13	..	..	5	1	296	1% (0–2)	5% (3–8)
Iceland	1994–2015	62	61%	8	2	255	3% (1–5)	16% (11–20)
Lithuania	2012–15	52	50%	7	1	334	5% (3–8)	36% (31–41)
Norway	2004–16	..	..	7	2	856	5% (4–7)	18% (15–21)
Sweden	1994–2018	61	67%	7	8	5203	3% (2–3)	26% (20–34)
UK	2000–16	53	67%	5	5	7497	4% (2–5)	21% (16–26)
**Western Europe**								
Austria	1997–2017	53	63%	5	2	577	4% (3–6)	37% (33–41)
Belgium	1995–2014	56	64%	7	4	950	4% (2–7)	16% (11–23)
France	1987–2015	56	67%	6	9	1363	7% (4–10)	24% (17–32)
Germany	1992–2019	55	67%	5	15	2250	10% (7–14)	19% (13–26)
Switzerland	..	..	..	2	1	35	11% (5–26)	37% (23–54)
The Netherlands	2007–14	52	68%	8	1	155	8% (5–14)	9% (5–15)
**Central and eastern Europe**								
Bulgaria	2016	52	63%	7	2	736	20% (18–23)	20% (17–23)
Czech Republic	1991–2013	55	58%	4	3	917	14% (2–32)	16% (11–21)
Hungary	2007–15	54	59%	5	2	294	6% (3–9)	25% (20–31)
Poland	1992–2014	47	57%	5	6	4466	19% (15–23)	24% (23–26)
Romania	2000–18	56	64%	6	4	1048	21% (9–35)	29% (20–39)
Russia	1996–2015	54	39%	5	4	2592	11% (2–27)	25% (13–39)
Slovakia	2014–21	49	61%	7	1	1383	2% (2–3)	4% (3–5)
Ukraine	..	48	42%	3	1	36	6% (2–18)	25% (14–41)
**Southern Europe**								
Albania	1995–2019	56	79%	5	4	581	52% (28–76)	6% (3–11)
Bosnia and Herzegovina	2007–11	56	64%	8	2	160	36% (29–44)	20% (14–26)
Greece	1995–2013	67	72%	7	5	1084	26% (14–40)	32% (27–36)
Italy	1986–2020	59	65%	7	36	13 419	15% (13–17)	50% (44–57)
North Macedonia	..	57	79%	3	1	70	16% (9–26)	9% (4–17)
Portugal	2003–19	53	74%	7	4	58 910	3% (1–6)	18% (6–33)
Serbia	1998	53	..	3	1	65	23% (15–35)	11% (5–21)
Spain	1990–2010	54	71%	6	11	2891	9% (8–11)	38% (34–42)
**Caribbean and Central America**								
Cuba	2005–19	57	58%	7	2	237	13% (9–17)	44% (38–51)
Guatemala	2015	54	48%	5	1	100	7% (3–14)	3% (1–8)
Mexico	1995–2018	59	61%	8	9	3536	1% (0–3)	26% (18–34)
**South America**								
Argentina	1995–2017	61	63%	7	3	650	3% (2–4)	31% (27–34)
Brazil	1990–2019	54	70%	7	18	5050	9% (6–14)	31% (23–39)
Colombia	2005–07	..	61%	9	1	89	8% (4–15)	8% (4–15)
Peru	1991–2004	63	55%	7	3	593	14% (8–21)	12% (10–15)
Uruguay	2015–18	57	63%	8	1	49	2% (0–11)	27% (16–40)
**Northern America**								
Canada	2000–17	57	61%	8	8	164 085	8% (4–13)	36% (22–50)
USA	1990–2019	57	62%	7	44	866 810	3% (3–4)	37% (34–39)
**South-Central Asia**								
Bangladesh	2012	43	72%	5	2	85	66% (56–76)	7% (2–14)
India	1992–2018	47	79%	7	42	13 729	18% (16–21)	15% (12–18)
Islamic Republic of Iran	2000–15	43	61%	5	10	2974	30% (24–38)	10% (6–14)
Kazakhstan	2011–17	..	..	4	1	83	57% (46–67)	14% (8–24)
Nepal	1998–2016	52	72%	7	3	366	18% (10–27)	11% (8–14)
Pakistan	1987–2021	49	62%	7	43	7958	18% (15–22)	68% (63–74)
Sri Lanka	2013–14	56	85%	7	1	107	2% (1–7)	0% (0–3)
**Western Asia**								
Israel	2002–19	66	61%	7	1	1048	11% (9–13)	42% (39–45)
Kuwait	2006–17	43	71%	8	2	124	8% (4–14)	48% (39–57)
Qatar	2004–12	53	83%	5	2	171	18% (12–24)	31% (24–38)
Saudi Arabia	1993–2013	56	55%	5	4	844	23% (13–35)	46% (21–72)
Turkey	1993–2018	55	63%	6	19	4292	39% (34–45)	17% (13–21)
**South-Eastern Asia**								
Cambodia	1990–91	..	..	7	1	53	45% (33–59)	34% (23–47)
Indonesia	1992	53	62%	6	2	164	24% (17–31)	54% (47–62)
Malaysia	2015–20	64	66%	6	2	75	25% (15–36)	20% (11–30)
Myanmar	1998–2000	..	..	5	1	81	27% (19–38)	41% (31–52)
Singapore	2002–17	52	60%	6	4	663	35% (23–47)	18% (10–28)
Thailand	1997–2019	56	64%	5	10	617	29% (25–33)	28% (21–34)
Vietnam	1998–2020	56	64%	7	3	250	35% (24–48)	24% (14–35)
**Eastern Asia**								
China	1984–2020	53	69%	6	39	17 935	68% (60–74)	7% (5–9)
Hong Kong, China	2012–16	64	67%	5	1	466	64% (59–68)	10% (8–13)
Taiwan, China	1991–2017	57	68%	7	14	27 958	39% (30–48)	30% (22–39)
Japan	1980–2019	68	62%	7	39	104 881	12% (11–13)	51% (47–56)
Mongolia	2000–09	49	45%	8	2	1019	51% (48–54)	47% (44–50)
South Korea	1991–2017	57	72%	7	31	23 034	52% (47–56)	7% (6–8)
**Sub-Saharan Africa**								
Burkina Faso	2012–14	47	..	5	1	191	76% (70–82)	15% (10–20)
Burundi	1991–92	47	68%	8	1	80	29% (20–39)	55% (44–65)
Cameroon	2019–20	50	73%	5	1	40	53% (37–67)	30% (18–45)
Democratic Republic of the Congo	2016–18	..	63%	8	1	190	35% (28–42)	11% (7–16)
Ethiopia	1992–2019	42	75%	8	2	180	33% (26–40)	32% (25–39)
Gabon	1990–98	45	..	8	1	67	33% (23–45)	36% (25–48)
Ghana	2015–20	46	72%	9	2	335	47% (41–52)	5% (3–8)
Kenya	..	40	53%	5	1	30	27% (14–44)	0 (0–11)
Mali	1998–99	..	..	9	1	53	53% (40–66)	15% (8–27)
Nigeria	2005–10	46	75%	6	2	173	49% (41–56)	5% (2–9)
Rwanda	1983–87	51	56%	8	1	79	16% (10–26)	48% (37–59)
Senegal	1995–96	39	..	9	1	25	84% (65–94)	0 (0–13)
South Africa	1991–92	48	64%	9	1	77	19% (12–30)	23% (15–34)
Gambia	1997–2001	43	63%	9	1	97	59% (49–68)	9% (5–17)
Uganda	2010–11	52	58%	9	2	184	13% (8–18)	5% (2–9)
**Northern Africa**								
Egypt	1992–2017	53	69%	6	15	1684	4% (2–7)	88% (82–93)
Morocco	2001–11	53	72%	6	1	360	26% (22–31)	60% (55–65)
Sudan	2006–07	49	62%	5	1	61	56% (43–67)	2% (0–9)
Tunisia	2000–15	61	45%	4	3	218	33% (21–45)	36% (17–57)

Studies were included if they reported the number of patients with HBV and HCV in the population. Empty cells indicate that this information was not available (eg, for age, proportion of men, or calendar year). Many studies did not report on HBV and HCV co-infection. For studies that did, we reassigned patients with HBV and HCV co-infections to only HBV or only HCV according to a ratio equivalent to the overall prevalence of the two viruses in the corresponding study. HBV=hepatitis B virus. HCV=hepatitis C virus.

* The corresponding studies with a full references list are cited in the appendix (pp 4–5).

† Quality score (on a scale of 0–10), including ten items relevant to our research question that cover various methodological aspects of data collection.

**Figure 2:**
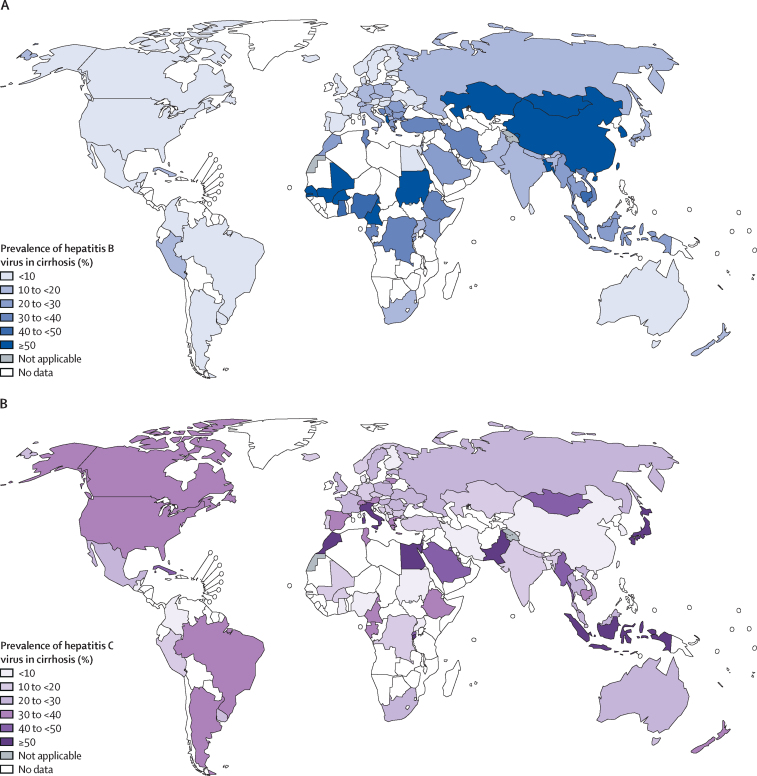
Country-specific pooled prevalence of hepatitis B virus (A) and hepatitis C virus (B) infection among patients with cirrhosis The designations used and the presentation of the material in this Article do not imply the expression of any opinion whatsoever on the part of WHO and the IARC about the legal status of any country, territory, city, or area, or of its authorities, or concerning the delimitation of its frontiers or boundaries.

**Figure 3:**
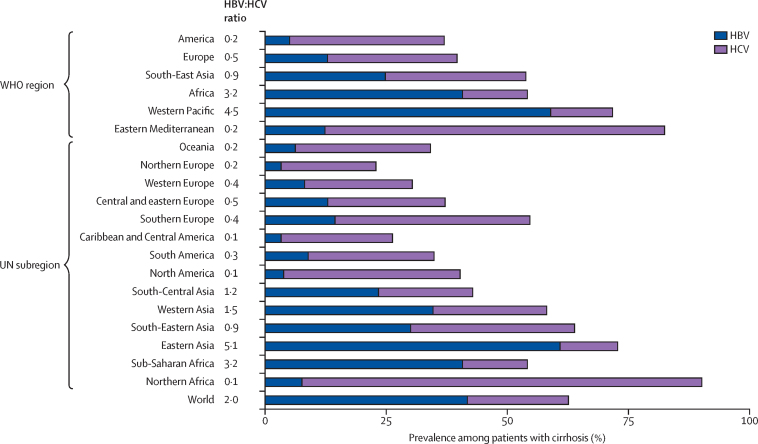
Regional and global pooled prevalence of HBV and HCV infection among patients with cirrhosis stratified by WHO region and UN subregion The HBV:HCV ratio column (centre) represents the relative importance of HBV over HCV per region and globally. HBV=hepatitis B virus. HCV=hepatitis C virus.

In most countries and regions from Europe, Oceania, and the Americas, HCV infection was more prevalent than was HBV infection in patients with cirrhosis (HBV UN subregional prevalence ranges 3–14%; HCV UN subregional prevalence ranges 20–40%; Table 1, Table 2; Figure 2, Figure 3). These regions also tended to have the largest proportion of cirrhosis unrelated to HBV or HCV, except for a few countries. In Italy, Greece, Albania, Bosnia and Herzegovina, and Cuba, the combined contribution of HBV and HCV infections to cirrhosis cases exceeded 50% (table 1).

**Table 2:** Pooled prevalence of HBV or HCV infection among patients with cirrhosis stratified by WHO region and UN subregion

	**HBV prevalence (%)**	**HCV prevalence (%)**	**Viral (%)**	**HBV/HCV ratio**
**WHO region**				
America	5%	32%	37%	0·2
Europe	13%	27%	40%	0·5
South-East Asia	25%	29%	54%	0·9
Africa	41%	13%	54%	3·2
Western Pacific	59%	13%	72%	4·5
Eastern Mediterranean	12%	70%	82%	0·2
**UN subregion**				
Oceania	6%	28%	34%	0·2
Northern Europe	3%	20%	23%	0·2
Western Europe	8%	22%	30%	0·4
Central and Eastern Europe	13%	24%	37%	0·5
Southern Europe	14%	40%	54%	0·4
Caribbean and Central America	3%	23%	26%	0·1
South America	9%	26%	35%	0·3
Northern America	4%	36%	40%	0·1
South-Central Asia	23%	19%	42%	1·2
Western Asia	35%	23%	58%	1·5
South-Eastern Asia	30%	34%	64%	0·9
Eastern Asia	61%	12%	73%	5·1
Sub-Saharan Africa	41%	13%	54%	3·2
Northern Africa	8%	83%	91%	0·1
World	42%	21%	63%	2·0

HBV=hepatitis B virus. HCV=hepatitis C virus.

In Asia, at the regional level, the prevalence of HBV infection was generally higher than HCV infection in patients with cirrhosis, with the exception of South-Eastern Asia where the ratio of HBV over HCV was 0·9 (HBV UN subregional prevalence ranges 23–61%; HCV UN subregional prevalence ranges 12–34%; table 2; figure 3). The ratio of HBV over HCV was highest in eastern Asia (5·1). In China—the largest country in the region and the one with the largest burden in that region—68% (95% CI 60–74) of patients with cirrhosis had HBV infection compared with 7% (5–9) with HCV infection. Exceptions to this pattern at country level were Pakistan, Japan, Saudi Arabia, Indonesia, Kuwait, Qatar, Israel, and Myanmar (table 1)**.**

In the northern African region, there was a predominance of HCV infection in cirrhosis (HBV UN subregional prevalence, 8%; HCV UN subregional prevalence, 83%; figure 3). Egypt had the highest HCV infection prevalence in the region (HBV, 4% [95% CI 2–7]; HCV, 88% [82–93]; table 1). Data from other countries in the region were few, but generally showed higher proportions of HBV than that seen in Egypt (table 1; figure 3).

In the sub-Saharan African region, the prevalence of HBV infection (HBV UN subregional prevalence, 41%) exceeded HCV infection (HCV UN subregional prevalence, 13%) in patients with cirrhosis (table 2). However, data were available from only 15 (31%) of the 48 countries, often represented by a single study with a small number of patients. HBV was predominant in western Africa (eg, Nigeria [49%, 41–56] and Mali [53%, 40–66]) when compared with other regions of sub-Saharan Africa (table 1; figure 2). The share of viral infection was high overall, with 11 (73%) of 15 countries having a combined prevalence of HBV and HCV infections exceeding 50%.

The prevalence of HBV infection was lower among patients with cirrhosis in Europe, the Americas, and Oceania (HBV UN subregional ranges 3–14%) than in Africa and Asia (HBV UN subregional ranges 8–61%). HCV infection prevalence was heterogenous, even within regions (HCV UN subregional ranges 12–83%). The combined prevalence of HBV and HCV infection exceeded 50% in most Asian and African regions.

The global prevalence of HBV infection in patients with cirrhosis was 42%, twice that of HCV infection (21%; figure 3; table 2).

Country-specific estimates were largely unaffected by using a fixed-effects instead of a random-effects model when estimating pooled estimates for each country, apart from the province of Taiwan (China), Canada, and Japan (appendix pp 4–5).

The pooled regional prevalence of HBV and HCV infection remained similar within a UN subregion independent of the weighting indicators used, except for northern Africa (appendix p 6). HBV estimates for northern Africa varied from 22% to 8% and HCV estimates from 60% to 83% when using the respective size of population instead of number of new liver cancer cases as weighting indicator (appendix p 6). Egypt, a country that has a high burden of HCV infection, contributed 42% to the northern African regional estimate when we used population size as a weighting indicator, and 88% when number of new liver cancer cases was used (data not shown).

The pooled global prevalence of HBV and HCV infections in patients with cirrhosis, as well as the relative contributions of the two viruses (HBV:HCV ratio) to cirrhosis, were highly sensitive to the weighting indicator used. The worldwide HBV:HCV ratio varied between 1 and 2, depending on the weighting indicator. For example, the HBV:HCV ratio was 2:1 (42% for HBV *vs* 21% for HCV) when using the number of new liver cancer cases as weighting indicator, 1·5:1 (32% *vs* 22%) when using the number of decompensated cirrhosis cases, or 1:1 (26% *vs* 27%) when using the number of cirrhosis deaths (appendix p 6). The weight (or relative contribution) of individual regions to the global estimates varied depending on the weighting indicator (appendix p 7). For example, eastern Asia contributed 54% to global estimates when the number of new liver cancer cases was used, 35% when the number of decompensated cirrhosis cases was used, or 15% when the number of cirrhosis deaths was used.

Sensitivity analyses did not considerably change the regional and global pooled HBV and HCV prevalence (appendix pp 8–9). The largest estimated change in viral prevalence was a decrease of 7% in western Europe and South-Eastern Asia when excluding studies with a score of less than 6, and an increase of 7% in northern Europe and a decrease of 7% in Oceania when excluding studies reporting on waiting list patients or transplant patients.

Data for heavy alcohol consumption was available from 444 studies done in 71 countries or territories, representing 82% of the world population. For NAFLD, data were available from 164 studies in 43 countries or territories, representing 66% of the world population. The relative contribution of heavy alcohol use to cirrhosis was high in countries from Europe (country range, 16–78%), the Americas (17–52%), and Oceania (15–37%), and lowest in Asia (0–41%, except for Nepal 67% [95% CI 31–95]; appendix pp 10–13). Data on NAFLD were available from only a few studies, predominantly in northern America, eastern Asia, and southern Europe. Countries with the largest amount of data and highest NAFLD prevalence were in north America (USA 13% [95% CI 10–15]; Canada 18% [3–41]; appendix pp 10–13).

## Discussion

This systematic review presents data on almost 1·4 million patients with cirrhosis, from 86 countries or territories that together represent 87% of the world population and provides the prevalence of HBV and HCV infection among patients with cirrhosis by country, region, and globally. The current work provides extensive empirical data at a country level for cirrhosis, a complex pathological change of the liver with multiple aetiologies. Most countries do not have a registry or systematic surveillance system for this condition.

Although the prevalence of viral infections in cirrhosis varied from one country to another, the contribution of HCV was generally higher in countries from the European and American regions, and the combined contribution of the two viruses in patients with cirrhosis was generally less than 50%. By contrast, in countries from African and Asian regions, HBV was more common (although with some exceptions), and the combined prevalence of both viruses among patients with cirrhosis usually exceeded 50%.

We estimated that the global pooled prevalence of HBV infection among patients with cirrhosis was twice that of HCV. Assuming these viruses to be the main drivers of the chronic pathological process leading to cirrhosis (ie, discounting a major contribution from other risk factors of liver disease), these results suggest that in 63% of patients worldwide, cirrhosis can be attributed to either HBV or HCV infection. These results are similar to previously published data on hepatocellular carcinoma (56% of hepatocellular carcinoma cases attributable to HBV infection and 20% of hepatocellular carcinoma cases attributable to HCV infection),^8^ and suggest that the contribution of HBV might be higher for hepatocellular carcinoma (56%) than for cirrhosis (42%). By contrast to HCV, HBV (and NAFLD) can cause hepatocellular carcinoma without going through a cirrhosis phase.^13^

In this study, we used the prevalence of HBV and HCV infections as an approximation of population attributable fractions. Since HBV and HCV infections are strongly associated with cirrhosis^19^ (and hepatocellular carcinoma^20^), the relative risk (RR) is high and therefore the population attributable fraction (=proportion of cases exposed × [RR–1]/[RR]) is close to the proportion of cases exposed in the respective population (eg, patients with cirrhosis).^8^, ^12^, ^21^ To obtain the absolute burden, the population attributable fractions should be applied to a mortality or incidence envelope (eg, the number of deaths or new cases from cirrhosis or hepatocellular carcinoma). Hepatocellular carcinoma incidence and mortality data are mostly derived from cancer registries that are available in many countries. Mortality due to cirrhosis is more difficult to estimate. There are currently no equivalent registries for cirrhosis, and mortality statistics are often unreliable.^4^, ^22^ This work cannot directly help estimate the mortality envelope from cirrhosis and therefore provides the relative, but not the absolute, burden of viral aetiologies to cirrhosis. We hope that our data will serve as input for future cause-specific cirrhosis estimates—for example, the GBD study.^10^, ^11^, ^23^

Although our study primarily focused on country-level estimates strictly derived from empirical data, we also provided regional and global estimates of the prevalence of HBV and HCV infection in cirrhosis. To calculate these pooled estimates, a country-specific weighting indicator was necessary. We chose to use the number of new liver cancer cases, rather than population size or any other cirrhosis indicator derived from GBD 2017. Apart from in northern Africa, where the choice of weighting indicator influenced the results, subanalyses indicated that regional estimates were generally robust to the weighting indicators used. By contrast, global estimates were highly sensitive to the choice of weighting indicator, driven by the large differences in prevalence of HBV and HCV by region and their respective weights. For example, eastern Asia contributed 15%, 14%, 35%, or 39% to the global burden when using GBD 2017 estimates for number of cirrhosis deaths, total DALYs lost due to cirrhosis, number of compensated cirrhosis cases, or number of decompensated cirrhosis cases as respective indicator, despite an expected high correlation between these indicators. Therefore, using different GBD 2017 cirrhosis weighting indicators resulted in substantially different aetiological fractions of HBV and HCV at a global level.

The aforementioned issues concerning extrapolation to larger geographical areas emphasise the relevance of data at a national level rather than at a global and (to a certain extent) regional level. The data presented in this work are a substantial step forward from the previous systematic review on this topic, published in 2006 and containing data on 29 countries.^12^ Here, we provide data on 86 countries or territories, systematically extracted from representative studies on patients with cirrhosis, published in the literature, following a standardised list of inclusion and exclusion criteria.

To assess the representativeness and quality of the data, we carried out several subanalyses and sensitivity analyses. We estimated pooled estimates for each country using a random-effects model for the main analysis and used a fixed-effects model for comparison purposes. Most pooled estimates were largely unchanged by the method used, except for the province of Taiwan (China), Canada, and Japan, where the difference between the two methods should be interpreted within their specific local context. For example, it is possible that country-specific estimates might have changed over time. Cirrhosis attributable fractions might have changed since 2010 in some countries such as Italy or Japan due to the regression of the epidemics of HCV infection, early and massive HBV vaccine implementation (as seen in the province of Taiwan, China), initiatives to test and cure HCV, or increasing prevalence or diagnosis of NAFLD and changes in heavy alcohol use due to shift in local policies.^24^, ^25^, ^26^ Analysing timetrends could shed light on these issues for future research. Furthermore, we did several sensitivity analyses (such as exclusion of studies with a score <6), which had little effect on the findings.

Several limitations can be mentioned. Our study pools data from a long time period and, by design, does not capture recent time trends. This study therefore best describes the situation before and around the time of the launch of the WHO global hepatitis elimination initiative in most countries. Despite our search strategy, the number of published studies meeting our inclusion criteria in some countries or regions was limited. In sub-Saharan Africa, only 15 of 48 countries were represented, with typically only one study per country. Although regional estimates could be indicative for countries where data are few, this extrapolation should be used with caution. For example, the prevalence of HCV infection in northern Africa varies from one country to another—including neighbouring countries —and pooled regional data cannot be used as a substitute for national empirical data. Furthermore, we relied on the authors’ statements for diagnosis or cause of cirrhosis. Although the use of anti-HCV serology could overestimate the prevalence of chronic HCV infection in general population surveys, anti-HCV is a good proxy to identify the aetiology of HCV among patients who already have cirrhosis. We also included studies that used other means of detection (eg, HCV RNA). The number of studies using HCV RNA represented less than 1% of the patients included, impeding further subanalysis. HBV might have been underestimated in some endemic areas of HBV, because a fraction of cirrhosis cases might have been associated with occult HBV.

We chose to present data on heavy alcohol use and NAFLD independently from that of viral hepatitis, for several reasons. First, our search strategy focused on hepatitis viruses, and the data we extracted opportunistically on heavy alcohol use and NAFLD might be an incomplete picture of the literature. Second, unlike HBV and HCV tests, the definition of heavy alcohol consumption and NAFLD is not standardised, making studies more difficult to combine and interpret. Finally, studies did not often provide information on the presence of multiple aetiologies. Despite these limitations, the high relative proportion of alcohol (and NAFLD) reported in Europe, the Americas, and Oceania was consistent with a 2021 study on the attributable fraction of alcohol consumption in cancer.^27^ With the increase in awareness of NAFLD as a risk factor for cirrhosis, we hope that the data gaps highlighted by this review can promote reporting NAFLD alongside other aetiologies.^28^

With the availability of effective interventions for the prevention or treatment of HBV and HCV, the data presented in this study will help to effectively allocate resources towards viral hepatitis elimination and to design interventions at the country level. However, continued collection and reporting of empirical data will also be necessary to monitor the effects of elimination efforts on mortality. Monitoring changes in the attributable burden over the next decades will be most accurately captured by representative sentinel surveillance undertaken by motivated teams, with strong commitment to collect data over time, good assessment of representativity of the population, and an adequate balance between simplicity and the quality of data.^21^, ^28^ Other key aetiologies such as alcohol and NAFLD should be considered in this monitoring effort and might become more important as the burden due to viral hepatitis decreases.

## Data sharing

Study level data can be extracted from original publications. Study level data not publicly available cannot be shared because of legal and ethical requirements regarding the resharing of data from those studies. Request for access to more detailed data or the code used to generate the reported estimates should be addressed to the corresponding author.

For the **study protocol** see https://www.crd.york.ac.uk/prospero/display_record.php?RecordID=149323

## Declaration of interests

FN declares advisory fees and travel grants from Gilead and AbbVie. All other authors declare no competing interests.
