# Supplementary material for: Worldwide prevalence of hepatitis B virus and hepatitis C virus among patients with cirrhosis at country, region, and global levels: a systematic review
Source: Lancet Gastroenterol Hepatol. 2022 May 14;7(8):724–35. doi: 10.1016/S2468-1253(22)00050-4 (PMC9259503; doi:10.1016/S2468-1253(22)00050-4)
Supplement: Supplementary appendix [file mmc1.pdf]

THE LANCET
Gastroenterology & Hepatology

Supplementary appendix

This appendix formed part of the original submission and has been peer reviewed. We post it as supplied by the authors.

Supplement to: Alberts CJ, Clifford GM, et al. Worldwide prevalence of hepatitis B virus and hepatitis C virus among patients with cirrhosis at country, region, and global levels: a systematic review. *Lancet Gastroenterol Hepatol* 2022; published online May 13. https://doi.org/10.1016/S2468-1253(22)00050-4.

Supplementary appendix

Supplementary material:

Supplementary Table 1	2
Supplementary Table 2	3
Supplementary Table 3	4
Supplementary Table 4.a	6
Supplementary Table 4.b	7
Supplementary Table 5	8
Supplementary Table 6	10
Supplementary Figure 1	13
Appendix 1	14

Supplementary Table 1: **Medline search strategy for the systematic review on cirrhosis, 1993-2021. Similar search strategy was used for EMBASE, Web of Science, and Scielo**

(HBV+HCV+cirr) OR (HBV+cirr+etiology) OR (HCV+cirr+etiology)	
**#28**	**#25 OR #26 OR #27**
Hepatitis C AND cirrhosis AND etiology	
**#27**	**#17 AND #24 AND #3**
Hepatitis B AND cirrhosis AND etiology	
**#26**	**#10 AND #24 AND #3**
Hepatitis B AND Hepatitis C AND cirrhosis	
**#25**	**#10 AND #17 AND #24**
Cirrhosis: MeSh + keywords	
**#24**	**#18 OR #19 OR #20 OR #21 OR #22 OR #23**
#23	(decompensat*[tiab] AND (hepatitis[tiab] OR hepatic[tiab] OR liver[tiab] OR cirrhotic[tiab] OR cirrhosis[tiab]))
#22	"End Stage Liver Disease" [MeSH]
#21	"chronic liver"[tiab]
#20	"liver"[tiab] AND "cirrhosis"[tiab]
#19	"liver cirrhosis"[tiab]
#18	"Liver Cirrhosis"[MeSH]
Hepatitis C: MeSh + keywords	
**#17**	**#11 OR #12 OR #13 OR #14 OR #15 OR #16**
#16	"hcv"[tw]
#15	"hepatitis c"[tw]
#14	"hep c"[tw]
#13	"hepatitis c antibodies"[MeSH]
#12	"hepacivirus"[MeSH]
#11	"hepatitis c"[MeSH]
Hepatitis B: MeSh + keywords	
**#10**	**#4 OR #5 OR #6 OR #7 OR #8 OR #9**
#9	"hbv"[tw]
#8	"hepatitis b"[tw]
#7	"hep b"[tw]
#6	"hepatitis b antigens"[MeSH]
#5	"hepatitis b virus"[MeSH]
#4	"hepatitis b"[MeSH]
Etiology	
**#3**	**#1 OR #2**
#2	etiology[tiab]
#1	aetiology[tiab]

tiab=title/abstract=words and numbers included in a citation's title, collection title, abstract, other abstract and keywords. English language abstracts are taken directly from the published article. If an article does not have a published abstract, NLM does not create one.
tw=text words=includes all words and numbers in the title, abstract, other abstract, MeSH terms, MeSH subheadings, publication types, substance names, personal name as subject, corporate author, secondary source, comment/correction notes, and other terms (see Other Term [OT] above) typically non-MeSH subject terms (keywords), including NASA Space Flight Mission, assigned by an organization other than NLM.
Mesh=MeSH term=The NLM Medical Subject Headings controlled vocabulary of biomedical terms that is used to describe the subject of each journal article in MEDLINE.
HBV=hepatitis B virus. HCV=hepatitis C virus.

Supplementary Table 2: **Inclusion and exclusion criteria, and scoring system – systematic-review on cirrhosis, 1993-2021**

Inclusion criteria:
- Series of patients with cirrhosis from an unselected, assumed to be representative, population (e.g. all consecutive patients hospitalized at a given care centre for a specified period)<br>- Patients with cirrhosis diagnosed with compensated or decompensated cirrhosis, on waiting list for transplantation* or undergoing transplantation.*<br>- Series of at least 20 patients with cirrhosis<br>- Studies reporting on both HBV and HCV infection prevalence among patients with cirrhosis, preferably providing results of serological testing using hepatitis B surface antigen (HBsAg) and antibodies against HCV (anti-HCV)
**Exclusion criteria:**
- Selection of patients based on aetiological diagnosis (e.g. selective inclusion or exclusion of patients with HBV, HCV, alcohol consumption, or NALFD)
- Non-representative group of patients (e.g. veterans, patients living with HIV infection, injecting drug users, migrants, prisoners, patients matched to a comparison group based on demographic data or other variables that could introduce a selection bias, etc..)
- Patients with cirrhosis and liver cancer (if HCC was diagnosed in >25% of the study population)
- Estimates provided as a result of modelled data (in contrast to observed empirical data)
- Unclear selection criteria resulting in a likely biased group (e.g., the proportion of patients with HBV or HCV adds up to 100%, or each etiological group has the same size)
- First generation enzyme-linked immunosorbent assay (ELISA) test used for HCV diagnosis or patients tested for HCV before 1992.
**Quality score of 10 items relevant to our research question that covers various methodological aspects of data collection:**
1. the aim of the study was to report on the underlying etiology of cirrhosis in a group of patients (Yes/No)
2. the study population was an unselected population (e.g. all patients, all consecutive patients, randomized patients) (Yes/No)
3. the inclusion and exclusion criteria of the patients in the study were described (Yes/No)
4. the definition† of cirrhosis was described (Yes/No)
5. the numerator(s) added up correctly to the denominator defined by the authors (Yes/No)
6. the geographical location of the patients was explicitly identified in the main text (Yes/No)
7. the study recruitment period was mentioned (Yes/No)
8. the data-source of the population was described (Yes/No)
9. the tests used for detecting HBV and HCV infection were mentioned (Yes/No)
10. collection of patient data was prospective or cross-sectional (in contrast to retrospective) (Yes/No))

HBV=hepatitis B virus. HCC=hepatocellular carcinoma. HCV=hepatitis C virus. NALFD=non-alcoholic fatty liver disease.

*Transplant populations were included if all aetiologies were clearly identified and the group identified as 'others' was not >15% and the HCC group was identified as a separate group such that this group could be excluded from the denominator and numerator. We then subsequently extracted data on all aetiologies identified to be associated with cirrhosis of the liver.

†Under definition of cirrhosis we considered the study's description or mentioning of the following items: development of clinical cirrhosis-related clinical complications, liver histology, liver imaging and/or non-invasive measurements of liver fibrosis stage (e.g. liver stiffness or other validated scoring systems).

***Supplementary table 3:* Pooled prevalence of HBV or HCV infection among patients with cirrhosis by country / territory, using fixed effect approach – systematic-review, 1993-2021**

Country / Territory	HBV (%)	(95%CI)	HCV (%)	(95%CI)	REF
Oceania					
Australia	4%	(4% — 5%)	23%	(22% — 24%)	1-6
New Zealand	15%	(11% — 19%)	31%	(25% — 36%)	1
Northern Europe					
Denmark	1%	(0% — 3%)	4%	(2% — 6%)	7,8
Estonia	4%	(2% — 10%)	24%	(17% — 34%)	9
Finland	1%	(0% — 2%)	5%	(3% — 8%)	7
Iceland	3%	(1% — 5%)	16%	(11% — 20%)	10,11
Lithuania	5%	(3% — 8%)	36%	(31% — 41%)	12
Norway	5%	(4% — 7%)	18%	(15% — 21%)	7,13
Sweden	3%	(2% — 3%)	28%	(27% — 29%)	7,11,14-17
United Kingdom	3%	(3% — 4%)	18%	(17% — 19%)	18-22
Western Europe					
Austria	4%	(3% — 6%)	37%	(33% — 41%)	23,24
Belgium	4%	(3% — 5%)	17%	(15% — 19%)	25-27
France (metropolitan)	7%	(5% — 8%)	25%	(22% — 27%)	28-36
Germany	9%	(8% — 10%)	19%	(18% — 21%)	37-51
Switzerland	11%	(5% — 26%)	37%	(23% — 54%)	52
the Netherlands	8%	(5% — 14%)	9%	(5% — 15%)	53
Central and Eastern Europe					
Bulgaria	20%	(18% — 23%)	20%	(17% — 23%)	13,54
Czech republic	6%	(5% — 8%)	16%	(14% — 19%)	55-57
Hungary	6%	(3% — 9%)	25%	(20% — 31%)	58,59
Poland	18%	(17% — 19%)	24%	(23% — 25%)	60-65
Romania	28%	(25% — 30%)	26%	(24% — 29%)	66-69
Russian Federation	9%	(8% — 10%)	27%	(25% — 29%)	70-73
Slovakia	2%	(2% — 3%)	4%	(3% — 5%)	74
Ukraine	6%	(2% — 18%)	25%	(14% — 41%)	75
Southern Europe					
Albania	47%	(42% — 51%)	6%	(4% — 8%)	76,77
Bosnia and Herzegovina	36%	(29% — 44%)	20%	(14% — 26%)	78,79
Greece	21%	(19% — 24%)	31%	(28% — 34%)	58,80-83
Italy	14%	(13% — 14%)	55%	(54% — 56%)	84-118
North Macedonia	16%	(9% — 26%)	9%	(4% — 17%)	119
Portugal	1%	(1% — 1%)	6%	(6% — 6%)	9,13,120,121
Serbia	23%	(15% — 35%)	11%	(5% — 21%)	122
Spain	9%	(8% — 10%)	36%	(34% — 37%)	123-133
Caribbean and Central America					
Cuba	13%	(9% — 17%)	44%	(38% — 51%)	134,135
Guatemala	7%	(3% — 14%)	3%	(1% — 8%)	136
Mexico	2%	(2% — 3%)	33%	(31% — 34%)	137-145
South America					
Argentina	3%	(2% — 4%)	31%	(27% — 34%)	146,147
Brazil	11%	(10% — 12%)	37%	(36% — 38%)	148-165
Colombia	8%	(4% — 15%)	8%	(4% — 15%)	166
Peru	17%	(14% — 20%)	12%	(10% — 15%)	167-169
Uruguay	2%	(0% — 11%)	27%	(16% — 40%)	170
Northern America					
Canada	6%	(5% — 6%)	13%	(13% — 13%)	171-178
United States of America	3%	(3% — 3%)	31%	(31% — 31%)	179-214
South-Central Asia					
Bangladesh	66%	(56% — 76%)	7%	(2% — 14%)	215,216
India	16%	(16% — 17%)	14%	(14% — 15%)	217-258
Islamic Republic of Iran	32%	(31% — 34%)	11%	(9% — 12%)	259-267
Kazakhstan	57%	(46% — 67%)	14%	(8% — 24%)	268
Nepal	17%	(14% — 21%)	11%	(8% — 14%)	269-271
Pakistan	20%	(19% — 21%)	68%	(66% — 69%)	272-314
Sri Lanka	2%	(1% — 7%)	0%	(0% — 3%)	315
Western Asia					
Israel	11%	(9% — 13%)	42%	(39% — 45%)	316
Kuwait	8%	(4% — 14%)	48%	(39% — 57%)	224,317

Qatar	18%	(12% — 24%)	31%	(24% — 38%)	318,319
Saudi Arabia	23%	(20% — 26%)	52%	(49% — 56%)	320-323
Turkey	38%	(37% — 40%)	18%	(17% — 19%)	324-342
South-Eastern Asia					
Cambodia	45%	(33% — 59%)	34%	(23% — 47%)	343
Indonesia	24%	(17% — 31%)	54%	(47% — 62%)	344,345
Malaysia	25%	(15% — 36%)	20%	(11% — 30%)	346,347
Myanmar	27%	(19% — 38%)	41%	(31% — 52%)	348
Singapore	40%	(36% — 43%)	15%	(12% — 18%)	224,349-351
Thailand	29%	(25% — 33%)	28%	(25% — 32%)	352-361
Viet Nam	34%	(28% — 40%)	23%	(18% — 29%)	362-364
Eastern Asia					
China	71%	(71% — 72%)	4%	(3% — 4%)	224,365-402
Hong Kong, China	64%	(59% — 68%)	10%	(8% — 13%)	403
Taiwan, China	29%	(29% — 30%)	19%	(19% — 20%)	404-416
Japan	8%	(8% — 9%)	35%	(35% — 35%)	417-453
Mongolia	51%	(48% — 54%)	47%	(44% — 50%)	454,455
Republic of Korea	50%	(49% — 51%)	7%	(7% — 8%)	224,456-482
Sub-Saharan Africa					
Burkina Faso	76%	(70% — 82%)	15%	(10% — 20%)	483
Burundi	29%	(20% — 39%)	55%	(44% — 65%)	484
Cameroon	53%	(37% — 67%)	30%	(18% — 45%)	485
Democratic People Republic of Congo	35%	(28% — 42%)	11%	(7% — 16%)	486
Ethiopia	33%	(26% — 40%)	32%	(25% — 39%)	487,488
Gabon	33%	(23% — 45%)	36%	(25% — 48%)	489
Ghana	47%	(41% — 52%)	5%	(3% — 8%)	490,491
Kenya	27%	(14% — 44%)	0%	(0% — 11%)	492
Mali	53%	(40% — 66%)	15%	(8% — 27%)	493
Nigeria	49%	(41% — 56%)	5%	(2% — 9%)	494,495
Rwanda	16%	(10% — 26%)	48%	(37% — 59%)	496
Senegal	84%	(65% — 94%)	0%	(0% — 13%)	497
South Africa	19%	(12% — 30%)	23%	(15% — 34%)	498
The Gambia	59%	(49% — 68%)	9%	(5% — 17%)	499
Uganda	13%	(8% — 18%)	5%	(2% — 9%)	500,501
Northern Africa					
Egypt	3%	(3% — 4%)	92%	(91% — 94%)	198,502-515
Morocco	26%	(22% — 31%)	60%	(55% — 65%)	516
Sudan	56%	(43% — 67%)	2%	(0% — 9%)	517
Tunisia	36%	(30% — 43%)	32%	(26% — 39%)	518-520

CI=confidence interval. HBV=hepatitis B virus. HCV=hepatitis C virus.

Studies were included if they reported the number of patients with HBV and HCV in the population. Many studies did not report on HBV and HCV co-infection. For those that did, we reassigned patients with dual infections to only HBV or only HCV according to a ratio equivalent to the overall prevalence of the two viruses in the corresponding study. For example, in a population of 100 patients, 20 patients had an HBV infection, 40 patients had an HCV infection, and six patients had a dual infection. In total 22 (=20+6*[20/(20+40)]) patients were assigned to the HBV group and 44 (=40+6*[40/(20+40)]) to the HCV group.

Supplementary table 4.a: **Regional and global pooled prevalence of HBV and HCV infection among patients with cirrhosis weighted by different (burden) indicators**

	Pooled prevalence of HBV (%)							Pooled prevalence of HCV (%)							HBV/HCV
	Weighting indicators*							Weighting indicators*							
	Pop.	Cirrhosis†				Cancer‡		Pop.	Cirrhosis†				Cancer‡		
	Size of population	Nr. cirrhosis death	DALY related to cirrhosis	Nr. decompensated cirrhosis	Nr. compensated cirrhosis	Nr. new liver cancer	**Diff (max-min)**	Size of population	Nr. cirrhosis death	DALY related to cirrhosis	Nr. decompensated cirrhosis	Nr. compensated cirrhosis	Nr. new liver cancer	**Diff (max-min)**	Range HBV/HCV ratio (min-max)
Oceania	7%	6%	6%	6%	7%	6%	**1%**	28%	28%	28%	28%	28%	28%	**0%**	(0.2 — 0.3)
Northern Europe	3%	3%	3%	3%	3%	3%	**0%**	20%	20%	20%	19%	20%	20%	**1%**	(0.2 — 0.2)
Western Europe	8%	9%	9%	8%	9%	8%	**1%**	21%	21%	21%	21%	21%	22%	**1%**	(0.4 — 0.4)
Central and Eastern Europe	12%	12%	11%	12%	12%	13%	**2%**	24%	25%	25%	24%	24%	24%	**1%**	(0.4 — 0.5)
Southern Europe	15%	14%	14%	15%	15%	14%	**1%**	38%	39%	38%	39%	40%	40%	**2%**	(0.4 — 0.4)
Caribbean and Central America	3%	2%	2%	2%	2%	3%	**1%**	25%	24%	24%	25%	25%	23%	**2%**	(0.1 — 0.1)
South America	9%	9%	9%	8%	9%	9%	**1%**	25%	27%	27%	24%	25%	26%	**3%**	(0.3 — 0.4)
North America	4%	4%	4%	4%	4%	4%	**0%**	36%	36%	36%	36%	36%	36%	**0%**	(0.1 — 0.1)
South-Central Asia	23%	23%	23%	24%	24%	23%	**1%**	20%	22%	23%	18%	19%	19%	**5%**	(1.0 — 1.3)
Western Asia	32%	31%	31%	31%	30%	35%	**5%**	27%	28%	29%	28%	30%	23%	**7%**	(1.0 — 1.5)
South-Eastern Asia	28%	28%	28%	29%	29%	30%	**2%**	41%	43%	43%	37%	38%	34%	**9%**	(0.7 — 0.9)
Eastern Asia	62%	58%	60%	58%	61%	61%	**4%**	11%	14%	12%	14%	11%	12%	**3%**	(4.1 — 5.6)
Sub-Saharan Africa	39%	40%	41%	41%	40%	41%	**2%**	14%	13%	13%	14%	14%	13%	**1%**	(2.8 — 3.2)
Northern Africa	22%	9%	10%	20%	18%	8%	**14%**	60%	80%	79%	63%	66%	83%	**23%**	(0.1 — 0.4)
**Global**	31%	26%	27%	32%	37%	42%	**16%**	22%	27%	26%	22%	20%	21%	**7%**	**(1.0 — 2.0)**

HBV=hepatitis B virus. HCV=hepatitis C virus. Nr=number. Pop.=population. Diff.=difference. Max=maximum. Min=minimum. DALY=Disability-Adjusted Life Year.

*Weighting indicator used to weigh country-level data to obtain regional and global estimate.

†Cirrhosis related indicators were extracted from GBD 2017, Lancet 2020.

‡Liver related indicator was extracted from GLOBOCAN 2020.

Supplementary table 4.b: **Weight of a region for different (burden) indicators**

	Population	Cirrhosis*				Cancer†
	Size of population	Nr. cirrhosis death	DALY related to cirrhosis	Nr. decompensated cirrhosis	Nr. compensated cirrhosis	Nr. new liver cancer
Oceania	<1%	<1%	<1%	<1%	<1%	<1%
Northern Europe	1%	1%	1%	2%	1%	1%
Western Europe	3%	3%	2%	5%	3%	3%
Central and Eastern Europe	4%	8%	8%	9%	7%	3%
Southern Europe	2%	2%	2%	4%	3%	3%
Caribbean and Central America	3%	4%	4%	4%	4%	2%
South America	5%	5%	4%	5%	2%	5%
Northern America	6%	5%	5%	6%	5%	3%
South-Central Asia	26%	25%	27%	11%	12%	6%
Western Asia	4%	2%	2%	4%	3%	1%
South-Eastern Asia	9%	13%	13%	3%	4%	11%
Eastern Asia	22%	15%	14%	35%	39%	54%
Sub-Saharan Africa	14%	12%	13%	9%	12%	4%
Northern Africa	3%	4%	4%	3%	4%	4%

Nr.=number. DALY=Disability-Adjusted Life Year.

*Cirrhosis related indicators were extracted from GBD 2017, Lancet 2020.

†Liver related indicators were extracted from GLOBOCAN 2020.

Due to rounding percentages do not always sum up to 100%.

Supplementary table 5: **Sensitivity analyses: impact of different sensitivity analyses on regional and global prevalence – systematic-review on cirrhosis, 1993-2021**

**Complete dataset**					
	Nr. Studies	Nr. Patients	HBV (%)	HCV (%)	Viral (%)
Oceania	9	13 316	6%	28%	34%
Northern Europe	22	14 905	3%	20%	23%
Western Europe	32	5 330	8%	22%	30%
Central and Eastern Europe	23	11 472	13%	24%	37%
Southern Europe	64	77 180	14%	40%	54%
Caribbean and Central America	12	3 873	3%	23%	26%
South America	26	6 431	9%	26%	35%
Northern America	52	1 030 895	4%	36%	40%
South-Central Asia	102	25 302	23%	19%	42%
Western Asia	28	6 479	35%	23%	58%
South-Eastern Asia	23	1 903	30%	34%	64%
Eastern Asia	126	175 293	61%	12%	73%
Sub-Saharan Africa	19	1 801	41%	13%	54%
Northern Africa	20	2 323	8%	83%	91%
World	558	1 376 503	42%	21%	63%
**Scenario 1** - excluding studies with a score of <6					
	Nr. Studies	Nr. Patients	HBV (%)	HCV (%)	Viral (%)
Oceania	7	13 262	6%	29%	35%
Northern Europe	13	6 433	3%	23%	26%
Western Europe	12	2 587	6%	17%	23%
Central and Eastern Europe	9	4 925	9%	29%	38%
Southern Europe	36	72 675	13%	44%	57%
Caribbean and Central America	11	3 773	2%	28%	30%
South America	14	5 210	10%	28%	38%
Northern America	25	844 390	4%	34%	38%
South-Central Asia	67	18 773	23%	19%	42%
Western Asia	14	3 861	31%	26%	57%
South-Eastern Asia	13	1 230	29%	28%	57%
Eastern Asia	58	159 709	57%	11%	68%
Sub-Saharan Africa	15	1 516	39%	14%	53%
Northern Africa	7	970	6%	85%	91%
World	301	1 139 314	39%	20%	59%

**Scenario 2** - excluding studies based on patients with 'compensated cirrhosis' only					
	Nr. Studies	Nr. Patients	HBV (%)	HCV (%)	Viral (%)
Oceania	9	13 316	6%	28%	34%
Northern Europe	22	14 905	3%	20%	23%
Western Europe	29	4 851	9%	22%	31%
Central and Eastern Europe	23	11 472	13%	24%	37%
Southern Europe	62	76 778	15%	40%	55%
Caribbean and Central America	11	3 757	3%	22%	25%
South America	25	5 619	9%	25%	34%
Northern America	50	1 030 436	4%	36%	40%
South-Central Asia	99	24 865	23%	19%	42%
Western Asia	28	6 479	35%	23%	58%
South-Eastern Asia	20	1 487	30%	34%	64%
Eastern Asia	119	174 067	61%	12%	73%
Sub-Saharan Africa	19	1 801	41%	13%	54%
Northern Africa	20	2 323	8%	83%	91%
World	536	1 372 156	42%	21%	63%
**Scenario 3** - excluding studies based on ‘waiting list patients’ or ‘transplant patients’					
	Nr. Studies	Nr. Patients	HBV (%)	HCV (%)	Viral (%)
Oceania	5	10 859	5%	22%	27%
Northern Europe	15	5 571	8%	22%	30%
Western Europe	19	3 290	6%	19%	25%
Central and Eastern Europe	19	8 048	13%	25%	38%
Southern Europe	46	71 018	14%	43%	57%
Caribbean and Central America	12	3 873	3%	23%	26%
South America	20	5 663	10%	26%	36%
Northern America	34	869 323	3%	34%	37%
South-Central Asia	91	23 157	24%	19%	43%
Western Asia	23	5 333	33%	23%	56%
South-Eastern Asia	19	1 667	30%	33%	63%
Eastern Asia	110	171 364	60%	12%	72%
Sub-Saharan Africa	19	1 801	41%	13%	54%
Northern Africa	18	2 194	8%	83%	91%
World	450	1 183 161	41%	20%	61%

HBV=hepatitis B virus. HCV=hepatitis C virus. Nr=number.

Supplementary table 6: **Pooled prevalence of heavy alcohol consumption and non-alcoholic fatty liver disease among patients with cirrhosis by country / territory – systematic-review, 1993-2021**

	Heavy alcohol use					NAFLD				
Country / Territory	Nr. of studies (n)	Nr. of patients (n)	Heavy alcohol consumption (%)	(95%CI)	REF†	Nr. of studies (n)	Nr. of patients (n)	NAFLD (%)	(95%CI)	REF†
Oceania										
Australia	8	13 044	37%	(31% — 42%)	1-6	7	13 016	10%	(6% — 16%)	1-5
New Zealand	1	272	15%	(12% — 20%)	1	1	272	18%	(14% — 23%)	1
Northern Europe										
Denmark	2	366	39%	(34% — 44%)	7,8	1	90	2%	(1% — 8%)	8
Estonia	1	98	40%	(31% — 50%)	9	1	98	0%	(0% — 4%)	9
Finland	1	296	24%	(20% — 30%)	7					
Iceland	2	255	40%	(34% — 46%)	10,11	1	157	22%	(16% — 29%)	10
Lithuania	1	334	51%	(46% — 57%)	12					
Norway	1	422	16%	(13% — 20%)	7					
Sweden	8	5203	41%	(27% — 56%)	7,11,14-17	4	3207	8%	(5% — 13%)	14,16
United Kingdom	5	7497	41%	(33% — 50%)	18-22	3	1048	13%	(11% — 15%)	19-21
Western Europe										
Austria	2	577	35%	(31% — 39%)	23,24	1	209	9%	(6% — 13%)	23
Belgium	4	950	65%	(62% — 68%)	25-27	2	434	9%	(6% — 12%)	25
France (metropolitan)	9	1363	65%	(53% — 76%)	28-36	2	232	4%	(2% — 7%)	30,33
Germany	15	2250	42%	(31% — 52%)	37-51	2	161	12%	(7% — 17%)	37,39
Switzerland	1	35	20%	(10% — 36%)	52					
The Netherlands	1	155	39%	(31% — 47%)	53	1	155	7%	(4% — 12%)	53
Central and Eastern Europe										
Czech republic	3	917	40%	(29% — 52%)	55-57					
Hungary	1	238	76%	(70% — 81%)	58	1	238	0%	(0% — 2%)	58
Poland	4	3 375	29%	(20% — 38%)	60,62-64					
Romania	4	1048	39%	(16% — 65%)	66-69					
Russian Federation	4	2592	40%	(32% — 49%)	70-73	1	1512	6%	(5% — 7%)	70
Slovakia	1	1383	61%	(59% — 64%)	74	1	1383	8%	(7% — 10%)	74
Ukraine	1	36	78%	(62% — 88%)	75					
Southern Europe										
Albania	4	581	40%	(17% — 65%)	76,77	3	325	5%	(3% — 8%)	76
Bosnia and Herzegovina	2	160	28%	(21% — 35%)	78,79					

Greece	5	1084	31%	(19% — 44%)	58,80-83	4	1038	4%	(0% — 17%)	58,80,82,83
Italy	31	10 984	22%	(18% — 27%)	84,85,87-90,93-101,103-117	10	5215	6%	(3% — 11%)	84,85,87-90,95,102,106
North Macedonia	1	70	51%	(40% — 63%)	119					
Portugal	3	58 810	59%	(30% — 85%)	9,120,121	1	59	7%	(3% — 16%)	9
Serbia	1	65	49%	(37% — 61%)	122					
Spain	11	2891	54%	(46% — 61%)	123-132					
Caribbean and Central America										
Cuba	2	237	17%	(12% — 22%)	134,135	1	116	9%	(5% — 15%)	134
Guatemala	1	100	52%	(42% — 62%)	136					
Mexico	9	3536	45%	(33% — 59%)	137-145	3	1537	10%	(1% — 26%)	137,139,142
South America										
Argentina	3	650	39%	(27% — 52%)	146,147	1	138	4%	(2% — 8%)	147
Brazil	17	4985	48%	(38% — 57%)	148-153,155-165	7	2480	2%	(1% — 3%)	148,151,153,155-158
Colombia	1	89	44%	(34% — 54%)	166					
Peru	3	593	32%	(24% — 40%)	167-169	1	475	3%	(1% — 4%)	168
Uruguay	1	49	31%	(20% — 45%)	170	1	49	16%	(9% — 29%)	170
Northern America										
Canada	8	164 085	27%	(20% — 36%)	171-178	7	164 015	18%	(3% — 41%)	171-177
United States of America	44	866 810	29%	(26% — 32%)	179-210,212-214	29	824 336	13%	(10% — 15%)	180-190,192-194,196,197,200-204,210
South-Central Asia										
Bangladesh	1	50	2%	(0% — 10%)	216	1	35	3%	(1% — 15%)	215
India	34	12 919	41%	(36% — 47%)	217-225,227-244,248,249,251,252,254,256	15	8867	9%	(5% — 14%)	218,220,222-227,230,233,234,237,239,241
Islamic Republic of Iran	4	368	5%	(0% — 15%)	261,262,266,267					
Nepal	3	366	67%	(31% — 95%)	269-271					
Pakistan	16	2487	3%	(2% — 5%)	274,276,281-283,286,288-290,293,298,301,305,307,308,312	5	1083	8%	(2% — 18%)	272,276,282,288,289
Sri Lanka	1	107	30%	(22% — 39%)	315				—	
Western Asia										
Israel	1	1048	18%	(16% — 21%)	316	1	1048	18%	(15% — 20%)	316
Kuwait	1	100	7%	(3% — 14%)	224	2	124	18%	(12% — 26%)	224,317
Qatar	2	171	33%	(26% — 40%)	318,319					
Saudi Arabia	2	521	2%	(1% — 3%)	321,323					
Turkey	19	4292	7%	(5% — 10%)	324-342	4	1151	8%	(1% — 20%)	324,325,327,336
South-Eastern Asia										
Indonesia	1	86	0%	(0% — 4%)	345					
Malaysia	2	75	10%	(4% — 18%)	346,347	2	75	13%	(6% — 21%)	346,347
Singapore	4	663	7%	(1% — 18%)	224,349-351	2	255	18%	(13% — 23%)	224,349
Thailand	9	552	28%	(14% — 46%)	352-360	8	497	5%	(3% — 7%)	352-359
Viet Nam	2	127	26%	(19% — 34%)	362,363	1	80	6%	(3% — 14%)	362
Eastern Asia										
China	32	17 411	11%	(7% — 15%)	224,365-371,373,374,376,377,379-388,390-395,397-400	2	1682	2%	(1% — 3%)	224,386
Hong Kong, China	1	466	5%	(3% — 7%)	403	1	466	18%	(14% — 21%)	403
Taiwan, China	11	22 290	26%	(18% — 35%)	404,405,407-409,411-413,415,416					

Japan	32	103 866	18%	(16% — 20%)	417-419,421,423-439,441,442,444-448,451,452	14	34 985	8%	(6% — 10%)	418,420,423-427,431,436-439
Republic of Korea	27	21 884	33%	(28% — 38%)	224,456-467,470-472,474-481	6	17 013	2%	(0% — 4%)	224,456,472,477
Sub-Saharan Africa										
Cameroon	1	40	18%	(9% — 32%)	485					
Ethiopia	1	24	17%	(7% — 36%)	487					
Ghana	2	335	42%	(37% — 48%)	490,491	2	335	2%	(0% — 3%)	490,491
Nigeria	1	40	38%	(24% — 53%)	495					
Rwanda	1	79	16%	(10% — 26%)	496					
Uganda	1	85	55%	(45% — 65%)	501					
Northern Africa										
Egypt	2	165	3%	(1% — 7%)	502,514	1	322	2%	(1% — 4%)	508
Morocco	1	360	1%	(0% — 2%)	516					
Tunisia	2	81	5%	(1% — 11%)	518,519					

NAFLD=Non-Alcoholic Fatty Liver Disease. CI=confidence interval. Nr=number. REF=reference.

†Listed references are cited under the corresponding number in Appendix 1.

Studies were only included if they reported the number of patients with HBV and HCV in the population. The etiologies of heavy alcohol consumption and NAFLD were extracted when this information was also reported, as a result not all countries have data on these etiologies and the number of studies and patients on which these estimates are based is lower than for HBV and HCV.
Empty cells mean that this information was not available for NAFLD.

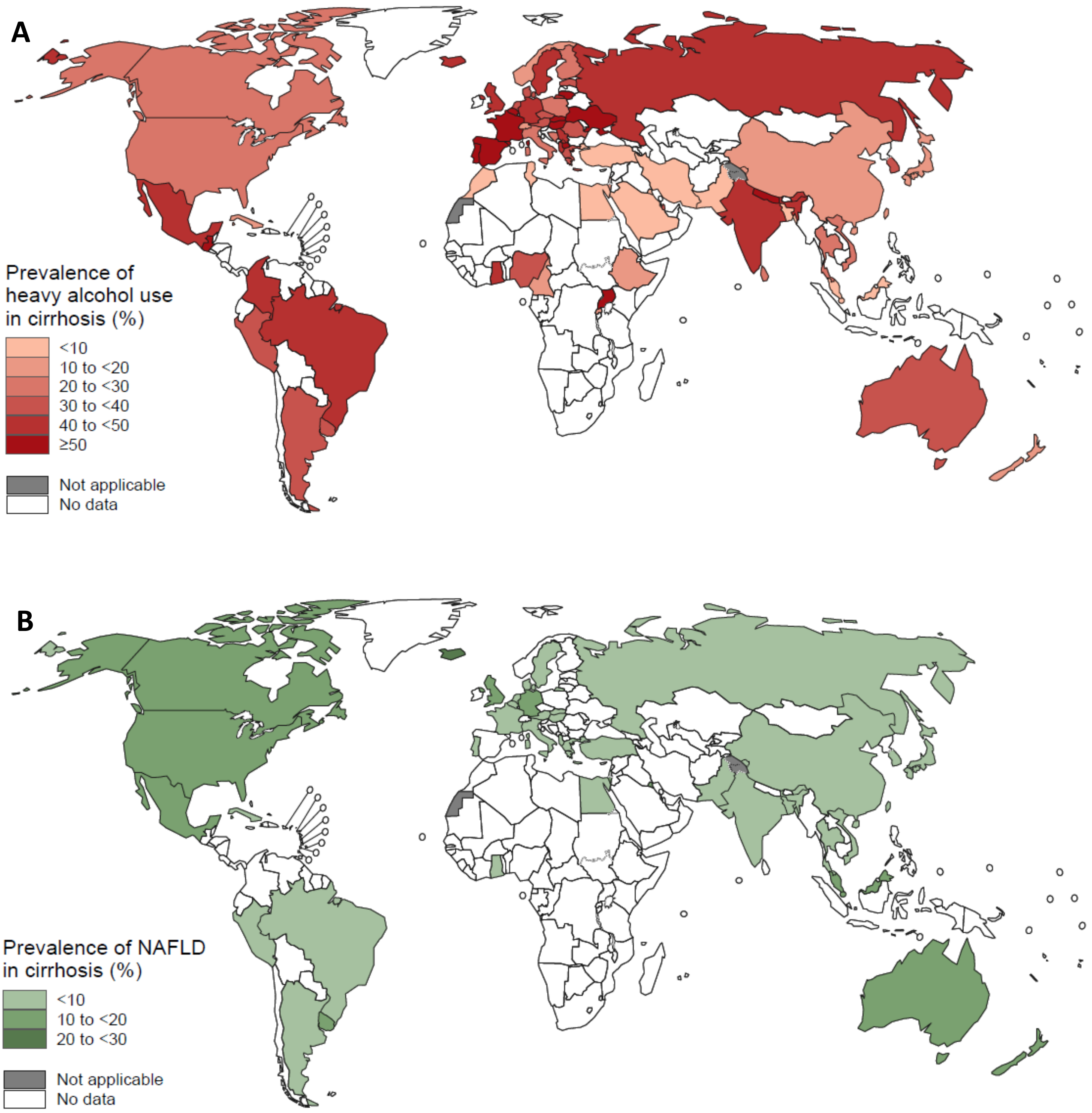

Supplementary Figure 1: **Country specific pooled prevalence of heavy alcohol use (A) and NAFLD (B) among patients with cirrhosis – systematic-review, 1993-2021**
NAFLD=Non-Alcoholic Fatty Liver Disease.

The designations used and the presentation of the material in this Article do not imply the expression of any opinion whatsoever on the part WHO and the IARC about the legal status of any country, territory, city, or area, or of its authorities, or concerning the delimitation of its frontiers or boundaries.

Appendix 1 – References

1. Howell J, Majumdar A, Fink M, et al. Turning the tide on hepatitis C-related liver transplantation: the return on investment in hepatitis C treatment in Australia and New Zealand. *Liver Transpl* 2021.
2. McPhail SM, Amarasena S, Stuart KA, et al. Assessment of health-related quality of life and health utilities in Australian patients with cirrhosis. *Jgh Open* 2021; **5**(1): 133-42.
3. Valery PC, McPhail S, Stuart KA, et al. Changing prevalence of aetiological factors and comorbidities among Australians hospitalised for cirrhosis. *Internal medicine journal* 2020.
4. Irvine KM, Wockner LF, Hoffmann I, et al. Multiplex Serum Protein Analysis Identifies Novel Biomarkers of Advanced Fibrosis in Patients with Chronic Liver Disease with the Potential to Improve Diagnostic Accuracy of Established Biomarkers. *PloS one* 2016; **11**(11).
5. Testro AG, Visvanathan K, Skinner N, et al. Acute allograft rejection in human liver transplant recipients is associated with signaling through toll-like receptor 4. *Journal of Gastroenterology and Hepatology (Australia)* 2011; **26**(1): 155-63.
6. Napoli J, Bishop GA, McCaughan GW. Increased intrahepatic messenger RNA expression of interleukins 2, 6, and 8 in human cirrhosis. *Gastroenterology* 1994; **107**(3): 789-98.
7. Fosby B, Melum E, Bjoro K, et al. Liver transplantation in the Nordic countries-An intention to treat and post-transplant analysis from the Nordic Liver Transplant Registry 1982-2013. *Scandinavian Journal of Gastroenterology* 2015; **50**(6): 797-808.
8. Thiele M, Askgaard G, Timm HB, Hamberg O, Gluud LL. Predictors of health-related quality of life in outpatients with cirrhosis: Results from a prospective cohort. *Hepatitis Research and Treatment* 2013; **2013**.
9. Pereira R, Buglevski M, Perdigoto R, et al. Intra-abdominal hypertension and abdominal compartment syndrome in the critically ill liver cirrhotic patient-prevalence and clinical outcomes. A multicentric retrospective cohort study in intensive care. *PloS one* 2021; **16**(5 May).
10. Olafsson S, Rögnvaldsson S, Bergmann OM, Jonasson JG, Benitez Hernandez U, Björnsson ES. A nationwide population-based prospective study of cirrhosis in Iceland. *JHEP Reports* 2021; **3**(3).
11. Gunnarsdottir SA, Olsson R, Olafsson S, et al. Liver cirrhosis in Iceland and Sweden: incidence, aetiology and outcomes. *Scand J Gastroenterol* 2009; **44**(8): 984-93.
12. Basyte-Bacevice V, Skieceviciene J, Valantiene I, et al. TM6SF2 and MBOAT7 Gene Variants in Liver Fibrosis and Cirrhosis. *International journal of molecular sciences* 2019; **20**(6).
13. Duffell E, Cortez-Pinto H, Simonova M, et al. Estimating the attributable fraction of cirrhosis and hepatocellular carcinoma due to hepatitis B and C. *J Viral Hepat* 2021.
14. Hagström H, Lindfors A, Holmer M, et al. Etiologies and outcomes of cirrhosis in a large contemporary cohort. *Scand J Gastroenterol* 2021: 1-6.
15. von Platen A, D'Souza MA, Rooyackers O, Nowak G. Intrahepatic Microdialysis for Monitoring of Metabolic Markers to Detect Rejection Early After Liver Transplantation. *Transplantation Proceedings* 2021; **53**(1): 130-5.
16. Vaz J, Eriksson B, Stromberg U, Buchebner D, Midlov P. Incidence, aetiology and related comorbidities of cirrhosis: a Swedish population-based cohort study. *Bmc Gastroenterology* 2020; **20**(1).
17. Kalaitzakis E, Olsson R, Henfridsson P, et al. Malnutrition and diabetes mellitus are related to hepatic encephalopathy in patients with liver cirrhosis. *Liver international : official journal of the International Association for the Study of the Liver* 2007; **27**(9): 1194-201.
18. Halliday N, Martin K, Collett D, Allen E, Thorburn D. Is liver transplantation out-of-hours' non-inferior to in-hours' transplantation? A retrospective analysis of the UK Transplant Registry. *BMJ Open* 2019; **9**(2).
19. Wiles R, Patanwala I, Hankinson B, et al. Can acoustic radiation force imaging of the liver and spleen predict the presence of gastroesophageal varices? *Clinical Radiology* 2018; **73**(12): 1046-51.
20. Fernandez Del Rio R, O'Hara ME, Holt A, et al. Volatile Biomarkers in Breath Associated With Liver Cirrhosis - Comparisons of Pre- and Post-liver Transplant Breath Samples. *EBioMedicine* 2015; **2**(9): 1243-50.
21. Cacciottolo TM, Gelson WT, Maguire G, Davies SE, Griffiths WJ. Pi*Z heterozygous alpha-1 antitrypsin states accelerate parenchymal but not biliary cirrhosis. *European journal of gastroenterology & hepatology* 2014; **26**(4): 412-7.
22. Rajoriya N, Forrest EH, Gray J, et al. Long-term follow-up of endoscopic histoacryl glue injection for the management of gastric variceal bleeding. *QJM* 2011; **104**(1): 41-7.
23. Schwarzer R, Reiberger T, Mandorfer M, et al. The von Willebrand Factor antigen to platelet ratio (VITRO) score predicts hepatic decompensation and mortality in cirrhosis. *Journal of Gastroenterology* 2020; **55**(5): 533-42.
24. Silberhumer GR, Pokorny H, Hetz H, et al. Combination of extended donor criteria and changes in the Model for End-Stage Liver Disease score predict patient survival and primary dysfunction in liver transplantation: a retrospective analysis. *Transplantation* 2007; **83**(5): 588-92.
25. Kaze E, Descamps OS, Henrion J. The changing pattern of cirrhosis in belgium: A study based on two cohorts pro-spectively collected 15 years apart. *Acta Gastro-Enterologica Belgica* 2020; **83**(4): 559-63.

26. Moreau N, Wittebole X, Fleury Y, Forget P, Laterre PF, Castanares-Zapatero D. Neutrophil-to-Lymphocyte Ratio Predicts Death in Acute-on-Chronic Liver Failure Patients Admitted to the Intensive Care Unit: A Retrospective Cohort Study. *Shock* 2018; **49**(4): 385-92.
27. Henrion J, De Maeght S, Deltenre P, et al. Impact of hepatitis C virus infection on the aetiology of cirrhosis and hepatocarcinoma in three affiliated hospitals in southern Belgium. *Acta gastro-enterologica Belgica* 2002; **65**(2): 80-2.
28. Javaud N, Bonnin L, Lapostolle F, et al. Prognosis of cirrhotic patients admitted to Emergency Departments: A multicenter study. *American Journal of Emergency Medicine* 2019; **37**(7): 1317-21.
29. Dercle L, Billey C, Cognet T, et al. Spleno-hepatic index to predict portal hypertension by equilibrium radionuclide ventriculography. *Nuclear Medicine Communications* 2018; **39**(12): 1138-42.
30. Goumard C, Perdigao F, Cazejust J, Zalinski S, Soubrane O, Scatton O. Is computed tomography volumetric assessment of the liver reliable in patients with cirrhosis? *HPB : the official journal of the International Hepato Pancreato Biliary Association* 2014; **16**(2): 188-94.
31. Eyraud D, Granger B, Ionescu C, et al. Thrombocytopenia, splenomegaly, and portal blood flow in patients who have undergone liver transplantation for cirrhosis. *Liver Transpl* 2012; **18**(3): 340-6.
32. Levesque E, Hoti E, Azoulay D, et al. Prospective evaluation of the prognostic scores for cirrhotic patients admitted to an Intensive Care Unit. *Journal of Hepatology* 2012; **56**(1): 95-102.
33. Nguyen-Khac E, Saint-Leger P, Tramier B, Coevoet H, Capron D, Dupas JL. Noninvasive Diagnosis of Large Esophageal Varices by Fibroscan: Strong Influence of the Cirrhosis Etiology. *Alcoholism-Clinical and Experimental Research* 2010; **34**(7): 1146-53.
34. Nguyen-Khac E, Thevenot T, Capron D, et al. Are ascitic electrolytes usable in cirrhotic patients? Correlation of sodium, potassium, chloride, urea, and creatinine concentrations in ascitic fluid and blood. *European Journal of Internal Medicine* 2008; **19**(8): 613-8.
35. Denie C, Poynard T, Gadano A, et al. [Influence of anemia on hemodynamic changes in patients with cirrhosis]. *Gastroenterologie clinique et biologique* 1997; **21**(1): 29-33.
36. GanneCarrie N, Chastang C, Chapel F, et al. Predictive score for the development of hepatocellular carcinoma and additional value of liver large cell dysplasia in western patients with cirrhosis. *Hepatology* 1996; **23**(5): 1112-8.
37. Sturm L, Roth L, Zoldan K, et al. Blood reelin in the progression of chronic liver disease. *Advances in Medical Sciences* 2021; **66**(1): 148-54.
38. Thude H, Tiede P, Marget M, Peine S, Nashan B, Koch M. Protein tyrosine phosphatase non-receptor type 22 (PTPN22) gene polymorphisms in liver transplant donors and impact on acute cellular liver transplant rejection. *HLA* 2020; **95**(1): 40-4.
39. Buechter M, Manka P, Gerken G, et al. Transjugular Intrahepatic Portosystemic Shunt in Patients with Portal Hypertension: Patency Depends on Coverage and Interventionalist's Experience. *Digestive Diseases* 2018; **36**(3): 218-27.
40. Busch CJ, Siegler BH, Werle H, et al. Risk factors for early viral infections after liver transplantation. *Langenbecks Archives of Surgery* 2018; **403**(4): 509-19.
41. Herden U, Fischer L, Sterneck M, Grabhorn E, Nashan B. Long-term follow-up after full-split liver transplantation and its applicability in the recent transplant era. *Clinical Transplantation* 2018; **32**(3).
42. Mollaiyan A, Bettinger D, Rössle M. The underdilation of nitinol stents at TIPS implantation: Solution or illusion? *European Journal of Radiology* 2017; **89**: 123-8.
43. Pischke S, Lege MC, von Wulffen M, et al. Factors associated with long-term survival after liver transplantation: A retrospective cohort study. *World journal of hepatology* 2017; **9**(8): 427-35.
44. Sivanathan V, Kittner JM, Sprinzl MF, et al. [Etiology and complications of liver cirrhosis: data from a German centre]. *Deutsche medizinische Wochenschrift (1946)* 2014; **139**(36): 1758-62.
45. Wiegand J, Kuhne M, Pradat P, Mossner J, Trepo C, Tillmann HL. Different patterns of decompensation in patients with alcoholic vs. non-alcoholic liver cirrhosis. *Aliment Pharmacol Ther* 2012; **35**(12): 1443-50.
46. Beckebaum S, Iacob S, Sweid D, et al. Efficacy, safety, and immunosuppressant adherence in stable liver transplant patients converted from a twice-daily tacrolimus-based regimen to once-daily tacrolimus extended-release formulation. *Transplant International* 2011; **24**(7): 666-75.
47. Weismuller TJ, Fikatas P, Schmidt J, et al. Multicentric evaluation of model for end-stage liver disease-based allocation and survival after liver transplantation in Germany--limitations of the 'sickest first'-concept. *Transplant international : official journal of the European Society for Organ Transplantation* 2011; **24**(1): 91-9.
48. Saner FH, Gensicke J, Olde Damink SW, et al. Neurologic complications in adult living donor liver transplant patients: an underestimated factor? *Journal of neurology* 2010; **257**(2): 253-8.
49. Grüngreiff K. Frequency of hepatic encephalopathy in liver cirrhosis: Retrospective 10-years-analysis of outpatients. *Verdauungskrankheiten* 2003; **21**(6): 263-8.
50. Broering DC, Topp S, Schaefer U, et al. Split liver transplantation and risk to the adult recipient: Analysis using matched pairs. *Journal of the American College of Surgeons* 2002; **195**(5): 648-57.

51. Su Q, Benner A, Hofmann WJ, Otto G, Pichlmayr R, Bannasch P. Human hepatic preneoplasia: Phenotypes and proliferation kinetics of foci and nodules of altered hepatocytes and their relationship to liver cell dysplasia. *Virchows Archiv* 1997; **431**(6): 391-406.
52. Sadallah S, Giostra E, Mentha G, Schifferli JA. Increased levels of soluble complement receptor 1 in serum patients with liver diseases. *Hepatology* 1996; **24**(1): 118-22.
53. Bot D, Droop A, Lucassen CJ, et al. Both muscle quantity and quality are predictors of waiting list mortality in patients with end-stage liver disease. *Clinical Nutrition ESPEN* 2021; **42**: 272-9.
54. Teoharov P, Mendisova A, Ivanova R, Kostov N, Mechkov G. Evidence for hepatitis B markers in cirrhotic patients without surface antigen (HBsAg). *Problems of Infectious and Parasitic Diseases* 1998; **26**(2): 26-7.
55. Petrtyl J, Bruha R, Urbanek P, Marecek Z, Kalab M. [Hepatic cein catheterisation - selected assessment aspects]. *Vnitrni lekarstvi* 2013; **59**(7): 587-90.
56. Trunečka P, Froněk J, Janoušek L, et al. The first 1,000 liver transplantations in IKEM. *Gastroenterologie a Hepatologie* 2013; **67**(5): 399-406.
57. Stransky J, Horejsova M, Chlumska A, Honzakova E, Vandasova J, Nemecek V. Prevalence of anti-HCV antibodies in chronic liver disease in the Czech Republic. *Infection* 1997; **25**(1): 49-50.
58. Gatselis NK, Tornai T, Shums Z, et al. Golgi protein-73: A biomarker for assessing cirrhosis and prognosis of liver disease patients. *World Journal of Gastroenterology* 2020; **26**(34): 5130-45.
59. Rokusz A, Nagy E, Gerlei Z, et al. Quantitative morphometric and immunohistochemical analysis and their correlates in cirrhosis - A study on explant livers. *Scandinavian Journal of Gastroenterology* 2016; **51**(1): 86-94.
60. Grąt M, Stypułkowski J, Patkowski W, et al. Challenging the principle of utility as a barrier for wider use of liver transplantation for hepatocellular cancer. *Annals of Surgical Oncology* 2017; **24**(11): 3188-95.
61. Krawczyk M, Grat M, Kornasiewicz O, et al. Results of liver transplantation in the Department of General, Transplant and Liver Surgery at the Medical University of Warsaw in patients with chronic hepatitis B and C viruses infection. *Przeglad epidemiologiczny* 2013; **67**(1): 5-10, 93-7.
62. Piekarska A, Zboinska J, Szymczak W, Kuydowicz J. Independent prognostic factors in patients with liver cirrhosis. *Hepato-Gastroenterology* 2008; **55**(84): 1034-40.
63. Petelenz M, Muslalik J, Mularczyk A, Zlotkowska R, Klimacka-Nawrot E. Hepatocellular carcicoma - Analysis of cases hospitalized from 1992 to 2006. *Experimental and Clinical Hepatology* 2007; **3**(1): 18-22.
64. Grzeszczuk A, Chlabicz S, Panasiuk A. Serum hyaluronan as a possible biomarker of liver insufficiency in cirrhotic patients. *Roczniki Akademii Medycznej w Bialymstoku (1995)* 2002; **47**: 80-5.
65. Panasiuk A. Autoantibodies in chronic liver diseases. *Roczniki Akademii Medycznej w Bialymstoku (1995)* 2001; **46**: 106-12.
66. Vecerzan L, Olteanu A, Maniu I, et al. Thrombin Generation in Chronic Liver Diseases-A Pilot Study. *Healthcare (Basel)* 2021; **9**(5).
67. Popescu I, Ionescu M, Brasoveanu V, et al. The Romanian National Program for Liver Transplantation - 852 Procedures in 815 Patients over 17 Years (2000-2017): A Continuous Evolution to Success. *Chirurgia (Bucharest, Romania : 1990)* 2017; **112**(3): 229-43.
68. Teiusanu A, Andrei M, Arbanas T, Nicolaie T, Diculescu M. Nutritional status in cirrhotic patients. *Maedica* 2012; **7**(4): 284-9.
69. Bota S, Sporea I, Şirli R, Popescu A, Dănilă M, Şendroiu M. The influence of liver residual mass on the values of acoustic radiation force impulse elastography (ARFI) in cirrhotic patients. *Medical Ultrasonography* 2011; **13**(3): 195-9.
70. Nadinskaia MY, Kodzoeva KB, Ulyanova KA, et al. Risk factors associated with portal vein thrombosis in liver cirrhosis: A case-control study. *Terapevticheskii arkhiv* 2019; **91**(2): 73-81.
71. Nepomnyashchikh DL, Vavilin VA, Aidagulova SV, et al. Cytochrome P450 2D6 polymorphism is a molecular genetic marker of liver cirrhosis progression. *Bulletin of experimental biology and medicine* 2012; **152**(5): 633-6.
72. Fisun A, Beliakin SA, Bobrov AN, Pliusnin SV, Khazanov AI. [Etiology of hepatic cirrhosis and its outcome in patients observed in a multidisciplinary hospital]. *Voenno-meditsinskii zhurnal* 2009; **330**(4): 17-23.
73. Zakirov IG. [Liver cirrhosis and liver cancer associated with viral hepatitis B and C in republic of Tatarstan]. *Zhurnal mikrobiologii, epidemiologii, i immunobiologii* 2003; (1): 26-8.
74. Skladany L, Jancekova D, Vnenčakova J, Selčanova SA, Bystrianska N, Švač J. Acute-on-chronic liver failure: A single-centre experience. *Clinical and Experimental Hepatology* 2020; **6**(2): 92-8.
75. Djiambou-Nganjeu H. Hepatic Encephalopathy in Patients in Lviv (Ukraine). *Journal of translational internal medicine* 2018; **6**(3): 146-51.
76. Xhimi T, Adriana B, Liri C, et al. Epidemiological evaluation of the liver cirrhosis in Albania: comparison with the historical data and indications to antiviral treatment appropriateness. *EASL conference, Poster Nr 1480* 2021.
77. Kraja B, Sina M, Mone I, et al. Predictive Value of the Model of End-Stage Liver Disease in Cirrhotic Patients with and without Spontaneous Bacterial Peritonitis. *Gastroenterology Research and Practice* 2012.

78. Prohic D, Mesihovic R, Vanis N, Puhalovic A. Prognostic Significance of Ascites and Serum Sodium in Patients with Low Meld Scores. *Medical archives (Sarajevo, Bosnia and Herzegovina)* 2016; **70**(1): 48-52.
79. Saray A, Mesihovic R, Vanis N, Gornjakovic S, Prohic D. Clinical significance of haemostatic tests in chronic liver disease. *Medical archives (Sarajevo, Bosnia and Herzegovina)* 2012; **66**(4): 231-5.
80. Karageorgos SA, Stratakou S, Koulentaki M, et al. Long-term change in incidence and risk factors of cirrhosis and hepatocellular carcinoma in crete, Greece: A 25-year study. *Annals of Gastroenterology* 2017; **30**(3): 357-63.
81. Bamias G, Gizis M, Delladetsima I, et al. Elevated Serum Levels of the Antiapoptotic Protein Decoy-Receptor 3 Are Associated with Advanced Liver Disease. *Canadian Journal of Gastroenterology and Hepatology* 2016; **2016**.
82. Giannousis IP, Papatheodoridis GV, Deutsch MJ, et al. The burden and recent epidemiological changes of the main chronic liver diseases in a Greek referral tertiary centre. *European journal of gastroenterology & hepatology* 2010; **22**(2): 172-9.
83. Tsiakalos A, Karatzaferis A, Ziakas P, Hatzis G. Acute-phase proteins as indicators of bacterial infection in patients with cirrhosis. *Liver International* 2009; **29**(10): 1538-42.
84. Brancaccio G, Vitale A, Signoriello G, Gaeta GB, Cillo U. Changing indications for liver transplant: slow decline of hepatitis viruses in Italy. *Infectious Diseases* 2020; **52**(8): 557-62.
85. Iavarone M, D'Ambrosio R, Soria A, et al. High rates of 30-day mortality in patients with cirrhosis and COVID-19. *Journal of Hepatology* 2020; **73**(5): 1063-71.
86. Ricco G, Cosma C, Bedogni G, et al. Modeling the time-related fluctuations of AFP and PIVKA-II serum levels in patients with cirrhosis undergoing surveillance for hepatocellular carcinoma. *Cancer Biomarkers* 2020; **29**(2): 189-96.
87. Khouri Chalouhi C, Vernuccio F, Rini F, et al. Hepatobiliary phase in cirrhotic patients with different Model for End-stage Liver Disease score: comparison of the performance of gadoxetic acid to gadobenate dimeglumine. *European Radiology* 2019; **29**(6): 3090-9.
88. Di Stefano C, Vanni E, Mirabella S, et al. Risk factors for arterial hypertension after liver transplantation. *Journal of the American Society of Hypertension* 2018; **12**(3): 220-9.
89. Ferrarese A, Germani G, Gambato M, et al. Hepatitis C virus related cirrhosis decreased as indication to liver transplantation since the introduction of direct-acting antivirals: A single-center study. *World journal of gastroenterology* 2018; **24**(38): 4403-11.
90. Stroffolini T, Sagnelli E, Gaeta GB, et al. Characteristics of liver cirrhosis in Italy: Evidence for a decreasing role of HCV aetiology. *European journal of internal medicine* 2017; **38**: 68-72.
91. Scalone L, Fagiuoli S, Ciampichini R, et al. The societal burden of chronic liver diseases: results from the COME study. *BMJ open gastroenterology* 2015; **2**(1): e000025.
92. Vitale A, Volk ML, De Feo TM, et al. A method for establishing allocation equity among patients with and without hepatocellular carcinoma on a common liver transplant waiting list. *Journal of hepatology* 2014; **60**(2): 290-7.
93. Pesce A, Scilletta R, Branca A, et al. Does transient elastography (FibroScan(R)) have a role in decision making in hepatocellular carcinoma? *HPB : the official journal of the International Hepato Pancreato Biliary Association* 2012; **14**(6): 403-8.
94. Angelico M, Cillo U, Fagiuoli S, et al. Liver Match, a prospective observational cohort study on liver transplantation in Italy: study design and current practice of donor-recipient matching. *Digestive and liver disease : official journal of the Italian Society of Gastroenterology and the Italian Association for the Study of the Liver* 2011; **43**(2): 155-64.
95. De Lisi S, Peralta S, Arini A, Simone F, Craxì A. Oesophagogastroduodenoscopy in patients with cirrhosis: Extending the range of detection beyond portal hypertension. *Digestive and Liver Disease* 2011; **43**(1): 48-53.
96. Zani C, Pasquale L, Bressanelli M, et al. The epidemiological pattern of chronic liver diseases in a community undergoing voluntary screening for hepatitis B and C. *Digestive and liver disease : official journal of the Italian Society of Gastroenterology and the Italian Association for the Study of the Liver* 2011; **43**(8): 653-8.
97. Cocito D, Maule S, Paolasso I, et al. High prevalence of neuropathies in patients with end-stage liver disease. *Acta Neurologica Scandinavica* 2010; **122**(1): 36-40.
98. Fabris C, Toniutto P, Falleti E, et al. MTHFR C677T polymorphism and risk of HCC in patients with liver cirrhosis: Role of male gender and alcohol consumption. *Alcoholism: Clinical and Experimental Research* 2009; **33**(1): 102-7.
99. Tarantino G, Citro V, Conca P, et al. What are the implications of the spontaneous spleno-renal shunts in liver cirrhosis? *BMC gastroenterology* 2009; **9**: 89.
100. Bolognesi M, Sacerdoti D, Mescoli C, et al. Different hemodynamic patterns of alcoholic and viral endstage cirrhosis: Analysis of explanted liver weight, degree of fibrosis and splanchnic Doppler parameters. *Scandinavian Journal of Gastroenterology* 2007; **42**(2): 256-62.
101. Gruttadauria S, Marsh JW, Cintorino D, et al. Adult to adult living-related liver transplant: Report on an initial experience in Italy. *Digestive and Liver Disease* 2007; **39**(4): 342-50.

102. Palmentieri B, de Sio I, La Mura V, et al. The role of bright liver echo pattern on ultrasound B-mode examination in the diagnosis of liver steatosis. *Digestive and Liver Disease* 2006; **38**(7): 485-9.
103. Amodio P, Valenti P, Del Piccolo F, et al. P300 latency for the diagnosis of minimal hepatic encephalopathy: Evidence that spectral EEG analysis and psychometric tests are enough. *Digestive and Liver Disease* 2005; **37**(11): 861-8.
104. Giannini EG, Risso D, Caglieris S, Testa R. Longitudinal modifications of the MELD score have prognostic meaning in patients with liver cirrhosis. *Journal of Clinical Gastroenterology* 2005; **39**(10): 912-4.
105. Burra P, Senzolo M, Pizzolato G, et al. Does liver-disease aetiology have a role in cerebral blood-flow alterations in liver cirrhosis? *European journal of gastroenterology & hepatology* 2004; **16**(9): 885-90.
106. Stroffolini T, Sagnelli E, Almasio P, Ferrigno L, Craxi A, Mele A. Characteristics of liver cirrhosis in Italy: results from a multicenter national study. *Digestive and liver disease : official journal of the Italian Society of Gastroenterology and the Italian Association for the Study of the Liver* 2004; **36**(1): 56-60.
107. Trevisani F, Castelli E, Foschi FG, et al. Impaired tuftsin activity in cirrhosis: Relationship with splenic function and clinical outcome. *Gut* 2002; **50**(5): 707-12.
108. Benvegnu L, Noventa F, Bernardinello E, Pontisso P, Gatta A, Alberti A. Evidence for an association between the aetiology of cirrhosis and pattern of hepatocellular carcinoma development. *Gut* 2001; **48**(1): 110-5.
109. Biancofiore G, Romanelli AM, Bindi ML, et al. Very early tracheal extubation without predetermined criteria in a liver transplant recipient population. *Liver Transplantation* 2001; **7**(9): 777-82.
110. Giannini E, Botta F, Fasoli A, et al. Progressive liver functional impairment is associated with an increase in AST/ALT ratio. *Digestive diseases and sciences* 1999; **44**(6): 1249-53.
111. Caradonna P, Costamagna G, Benedetti G, Gentiloni N, Gasbarrini GB. Chronic pancreatitis prevalence in liver cirrhosis. Morphological and functional study. *The Italian journal of gastroenterology* 1996; **28**(2): 91-4.
112. Russello M, Benigno R, Zammataro M. Hepatitis C virus in elderly patients with chronic liver diseases. *Archives of gerontology and geriatrics* 1996; **22 Suppl 1**: 317-20.
113. Montalto G, Soresi M, Tripi S, et al. [Incidence of anti-HCV positivity in a Sicilian population with liver diseases]. *Annali italiani di medicina interna : organo ufficiale della Societa italiana di medicina interna* 1995; **10**(2): 89-92.
114. Bellentani S, Tiribelli C, Saccoccio G, et al. Prevalence of chronic liver disease in the general population of northern Italy: the Dionysos Study. *Hepatology* 1994; **20**(6): 1442-9.
115. Benvegnu L, Fattovich G, Noventa F, et al. Concurrent hepatitis B and C virus infection and risk of hepatocellular carcinoma in cirrhosis. A prospective study. *Cancer* 1994; **74**(9): 2442-8.
116. Conte D, Fraquelli M, Mandelli C, et al. Probability of developing gallstones and related risk factors in 400 cirrhotics. *European Journal of Gastroenterology and Hepatology* 1994; **6**(1): 55-8.
117. De Bac C, Stroffolini T, Gaeta GB, Taliani G, Giusti G. Pathogenic factors in cirrhosis with and without hepatocellular carcinoma: a multicenter Italian study. *Hepatology* 1994; **20**(5): 1225-30.
118. Tabone M, Galatola G, Corrao G, Arico S. Anti-HCV antibodies in patients with chronic liver disease and different amounts of alcohol intake: A multivariate analysis. *European Journal of Gastroenterology and Hepatology* 1993; **5**(9): 749-54.
119. Ristovska EC, Genadieva-Dimitrova M, Caloska-Ivanova V, Misevski J. Von-willebrand factor as a predictor of three-month mortality in patients with liver cirrhosis compared to MELD score. *Acta Gastro-Enterologica Belgica* 2019; **82**(4): 487-93.
120. Silva MJ, Rosa MV, Nogueira PJ, Calinas F. Ten years of hospital admissions for liver cirrhosis in Portugal. *European journal of gastroenterology & hepatology* 2015; **27**(11): 1320-6.
121. Santos C, Marcelino P, Carvalho T, et al. The value of tubular enzymes for early detection of acute kidney injury after liver transplantation: An observational study. *Transplantation Proceedings* 2010; **42**(9): 3639-43.
122. Nikolic JA, Todorovic V, Bozic M, et al. Serum insulin-like growth factor (IGF)-II is more closely associated with liver dysfunction than is IGF-I in patients with cirrhosis. *Clinica chimica acta; international journal of clinical chemistry* 2000; **294**(1-2): 169-77.
123. Grilo Bensusan I, Pascasio JM, López-Pardo FJ, et al. Hepatopulmonary syndrome: Which blood gas analysis criteria and position should we use for diagnosis? *Revista Espanola de Enfermedades Digestivas* 2017; **109**(12): 843-9.
124. Alcalde Vargas A, Pascasio Acevedo JM, Gutierrez Domingo I, et al. Prevalence and characteristics of bone disease in cirrhotic patients under evaluation for liver transplantation. *Transplantation proceedings* 2012; **44**(6): 1496-8.
125. Gutierrez Domingo I, Pascasio Acevedo JM, Alcalde Vargas A, et al. Prevalence of hepatitis B and A virus markers and vaccination indication in cirrhotic patients evaluated for liver transplantation in Spain. *Transplantation proceedings* 2012; **44**(6): 1502-4.
126. Muro M, López-Álvarez MR, Campillo JA, et al. Influence of human leukocyte antigen mismatching on rejection development and allograft survival in liver transplantation: Is the relevance of HLA-A locus matching being underestimated? *Transplant Immunology* 2012; **26**(2-3): 88-93.

127. Lo Iacono O, Rincon D, Hernando A, et al. Serum levels of soluble vascular cell adhesion molecule are related to hyperdynamic circulation in patients with liver cirrhosis. *Liver international : official journal of the International Association for the Study of the Liver* 2008; **28**(8): 1129-35.
128. Romo EM, Munoz-Robles JA, Castillo-Rama M, et al. Peripheral blood lymphocyte populations in end-stage liver diseases. *Journal of clinical gastroenterology* 2007; **41**(7): 713-21.
129. Santoyo J, Suarez MA, Fernandez-Aguilar JL, et al. True impact of the indication of cirrhosis and the MELD on the results of liver transplantation. *Transplantation proceedings* 2006; **38**(8): 2462-4.
130. Velazquez RF, Rodriguez M, Navascues CA, et al. Prospective analysis of risk factors for hepatocellular carcinoma in patients with liver cirrhosis. *Hepatology* 2003; **37**(3): 520-7.
131. Fernandez E, Rodrigo L, Riestra S, Carcia S, Gutierrez F, Ocio G. Adenosine deaminase isoenzymes and neopterin in liver cirrhosis. *Journal of clinical gastroenterology* 2000; **30**(2): 181-6.
132. Macias Rodriguez MA, Rendon Unceta P, Tejada Cabrera M, et al. Risk factors for hepatocellular carcinoma in patients with liver cirrhosis. *Revista espanola de enfermedades digestivas : organo oficial de la Sociedad Espanola de Patologia Digestiva* 2000; **92**(7): 458-69.
133. Macias-Rodriguez MA, Rendon-Unceta P, Martinez-Sierra MC, Teyssiere-Blas I, Diaz-Garcia F, Martin-Herrera L. Prognostic usefulness of ultrasonographic signs of portal hypertension in patients with child-pugh stage A liver cirrhosis. *Am J Gastroenterol* 1999; **94**(12): 3595-600.
134. Castellanos-Fernández MI, Borges-González SA, Stepanova M, et al. Health-related quality of life in Cuban patients with chronic liver disease: A real-world experience. *Annals of Hepatology* 2021; **22**.
135. Castellanos Fernández MI, Santana Porbén S, García Jordá E, et al. Influence of hyponutrition on occurrence of complications and mortality among cirrhosis patients. *Nutricion Hospitalaria* 2008; **23**(1): 68-74.
136. Alvarez CS, Hernandez E, Escobar K, et al. Aflatoxin B(1)exposure and liver cirrhosis in Guatemala: a case-control study. *Bmj Open Gastroenterology* 2020; **7**(1).
137. Méndez-Guerrero O, Calle-Rodas DA, Cervantes-Alvarez E, et al. Renal and brain failure predict mortality of patients with acute-on-chronic liver failure admitted to the intensive care unit. *Annals of Hepatology* 2021; **22**.
138. Romano-Munive AF, Tellez-Ávila FI. Bleeding from gastrointestinal ectopic varices is not associated with haemorrhage from oesophageal or gastric varices. *Przeglad Gastroenterologiczny* 2020; **15**(1): 60-4.
139. Mendez-Sanchez N, Zamarripa-Dorsey F, Panduro A, et al. Current trends of liver cirrhosis in Mexico: Similitudes and differences with other world regions. *World journal of clinical cases* 2018; **6**(15): 922-30.
140. Torres-Valadez R, Roman S, Jose-Abrego A, et al. Early Detection of Liver Damage in Mexican Patients with Chronic Liver Disease. *Journal of translational internal medicine* 2017; **5**(1): 49-57.
141. Gonzalez-Ojeda A, Cervantes-Guevara G, Chavez-Sanchez M, et al. Platelet count/spleen diameter ratio to predict esophageal varices in Mexican patients with hepatic cirrhosis. *World journal of gastroenterology* 2014; **20**(8): 2079-84.
142. González-González JA, García-Compean D, Vázquez-Elizondo G, Garza-Galindo A, Jáquez-Quintana JO, Maldonado-Garza H. Nonvariceal upper gastrointestinal bleeding in patients with liver cirrhosis. Clinical features, outcomes and predictors of in-hospital mortality. A prospective study. *Annals of Hepatology* 2011; **10**(3): 287-95.
143. Mendez-Sanchez N, Aguilar-Ramirez JR, Reyes A, et al. Etiology of liver cirrhosis in Mexico. *Annals of hepatology* 2004; **3**(1): 30-3.
144. Gongora-Biachi RA, Castro-Sansores CJ, Gonzalez-Martinez P, Lara-Perera DM, Garrido-Palma J, Lara-Perera V. Frequency of antibodies against the hepatitis C virus in patients with hepatic cirrhosis in Yucatan, Mexico. *Salud publica de Mexico* 2003; **45**(5): 346-50.
145. Campollo O, Valencia-Salinas JJ, Berumen-Arellano A, Perez-Aranda MA, Panduro-Cerda A, Segura-Ortega J. [Epidemiological characteristics of liver cirrhosis at the Hospital Civil of Guadalajara]. *Salud publica de Mexico* 1997; **39**(3): 195-200.
146. García DS, Artola YM, Poncino DA, Rubino MF, Escobar R, Khoury M. Abstract etiology of cirrhosis: Epidemiological changes between the periods 1995-2002 and 2010-2017. *Acta Gastroenterologica Latinoamericana* 2020; **50**(3): 271-8.
147. Haddad L, Marciano S, Cleres M, et al. Characteristics of Liver Transplantation in Argentina: A Multicenter Study. *Transplantation proceedings* 2018; **50**(2): 478-84.
148. Santos-Lins LS, Aguiar ILS, Codes L, et al. Poor oral health-related quality of life in pre-and post-liver transplantation patients. *Open Dentistry Journal* 2021; **15**: 196-203.
149. Vaz NF, da Cunha VNR, Cunha-Silva M, Sevá-Pereira T, de Souza Almeida JR, Mazo DF. Evolution of diagnostic criteria for acute kidney injury in patients with decompensated cirrhosis: A prospective study in a tertiary university hospital. *Clinics and Research in Hepatology and Gastroenterology* 2020; **44**(4): 551-63.
150. Augustinho FC, Zocche TL, Borgonovo A, et al. Applicability of Sepsis-3 criteria and quick Sequential Organ Failure Assessment in patients with cirrhosis hospitalised for bacterial infections. *Liver International* 2019; **39**(2): 307-15.
151. Chueiri Neto F, Emídio LA, Perales SR, et al. Bloodstream Infections in Early Postsurgery Liver Transplant: An Analysis of 401 Patients Over 10 Years. *Transplantation Proceedings* 2019; **51**(6): 1972-7.

152. Amaral AED, Rode MP, Cisilotto J, et al. MicroRNA profiles in serum samples from patients with stable cirrhosis and miRNA-21 as a predictor of transplant-free survival. *Pharmacological research* 2018; **134**: 179-92.
153. Appel-da-Silva MC, Miozzo SA, Dossin IA, Tovo CV, Branco F, de Mattos AA. Incidence of hepatocellular carcinoma in outpatients with cirrhosis in Brazil: A 10-year retrospective cohort study. *World journal of gastroenterology* 2016; **22**(46): 10219-25.
154. Freitas AC, Shiguihara RS, Monteiro RT, Pazeto TL, Coelho JC. COMPARATIVE STUDY ON LIVER TRANSPLANTATION WITH AND WITHOUT HEPATOCELLULAR CARCINOMA WITH CIRRHOSIS: ANALYSIS OF MELD, WAITING TIME AND SURVIVAL. *Arquivos brasileiros de cirurgia digestiva : ABCD = Brazilian archives of digestive surgery* 2016; **29**(1): 21-5.
155. John JA, de Mattos AA, da Silva Miozzo SA, et al. Survival and risk factors related to death in outpatients with cirrhosis treated in a clinic in Southern Brazil. *European journal of gastroenterology & hepatology* 2015; **27**(12): 1372-7.
156. Garcia JHP, Mesquita DFG, Coelho GR, et al. Results from a liver transplant center in northeastern Brazil that performed more than 100 transplants in 2011. *Transplantation Proceedings* 2014; **46**(6): 1803-6.
157. Gonçalves PL, Gonçalves CS, Pereira FE. Mortality from liver cirrhosis in Espírito Santo State, Brazil. *Cadernos de saúde pública* 2014; **30**(6): 1335-40.
158. Paranagua-Vezozzo DC, Ono SK, Alvarado-Mora MV, et al. Epidemiology of HCC in Brazil: incidence and risk factors in a ten-year cohort. *Annals of hepatology* 2014; **13**(4): 386-93.
159. Thiele GB, da Silva OM, Fayad L, et al. Clinical and laboratorial features of spontaneous bacterial peritonitis in southern Brazil. *Sao Paulo Medical Journal* 2014; **132**(4): 205-10.
160. Goncalves PL, Zago-Gomes Mda P, Marques CC, Mendonca AT, Goncalves CS, Pereira FE. Etiology of liver cirrhosis in Brazil: chronic alcoholism and hepatitis viruses in liver cirrhosis diagnosed in the state of Espirito Santo. *Clinics (Sao Paulo, Brazil)* 2013; **68**(3): 291-5.
161. de Carvalho FM, Pereira TD, Goncalves PL, Jarske RD, Pereira FEL, Louro ID. Hepatocellular carcinoma and liver cirrhosis TP53 mutation analysis reflects a moderate dietary exposure to aflatoxins in Espirito Santo State, Brazil. *Molecular Biology Reports* 2013; **40**(8): 4883-7.
162. Silva LD, Rocha AM, Rocha GA, et al. The presence of Helicobacter pylori in the liver depends on the Th1, Th17 and Treg cytokine profile of the patient. *Memorias do Instituto Oswaldo Cruz* 2011; **106**(6): 748-54.
163. Gotardo DR, Strauss E, Teixeira MC, Machado MC. Liver transplantation and quality of life: relevance of a specific liver disease questionnaire. *Liver international : official journal of the International Association for the Study of the Liver* 2008; **28**(1): 99-106.
164. Strauss E, da Costa MF. The importance of bacterial infections as precipating factors of chronic hepatic encephalopathy in cirrhosis. *Hepato-gastroenterology* 1998; **45**(21): 900-4.
165. Gaburri D, Gaburri AK, Hubner E, et al. [Intestinal parasitosis and hepatic cirrhosis]. *Arquivos de gastroenterologia* 1997; **34**(1): 7-12.
166. Cortes-Mancera F, Loureiro CL, Hoyos S, et al. Etiology and viral genotype in patients with end-stage liver diseases admitted to a hepatology unit in Colombia. *Hepatitis Research and Treatment* 2011; **2011**.
167. Marcos LA, Bussalleu A, Terashima A, Espinoza JR. Detection of antibodies against Fasciola hepatica in cirrhotic patients from Peru. *Journal of helminthology* 2009; **83**(1): 23-6.
168. Bustíos C, Dávalos M, Román R, Zumaeta E. Características Epidemiológicas y Clínicas de la Cirrosis Hepática en la Unidad de Hígado del HNERM Es-Salud

[Clinical and epidemiological profile of cirrhosis in the liver unit at Edgardo Rebagliati Martins National Hospital]. *Revista de Gastroenterología del Perú* 2007; **27**(3): 238-45.
169. Barham WB, Figueroa R, Phillips IA, Hyams KC. Chronic liver disease in Peru: role of viral hepatitis. *Journal of medical virology* 1994; **42**(2): 129-32.
170. Perez L, Pincelli V, Pittamiglio L, et al. Fibroscan como diagnóstico de hipertensión portal en pacientes cirróticos. *Revista Uruguaya de Medicina Interna* 2019; **4**(3): 26-34.
171. Flemming JA, Djerboua M, Groome PA, Booth CM, Terrault NA. NAFLD and Alcohol-Associated Liver Disease Will Be Responsible for Almost All New Diagnoses of Cirrhosis in Canada by 2040. *Hepatology* 2021.
172. Philip G, Djerboua M, Carlone D, Flemming JA. Validation of a hierarchical algorithm to define chronic liver disease and cirrhosis etiology in administrative healthcare data. *PloS one* 2020; **15**(2).
173. Le Cleac' HA, Villeneuve JP, Sylvestre MP, Huard G, Giard JM, Ditisheim S. Gastric antral vascular ectasia is more frequent in patients with non-alcoholic steatohepatitis-induced cirrhosis. *Canadian Liver Journal* 2019; **2**(3): 84-90.
174. Chakraborty D, Akbari A, Knoll GA, et al. Serum BTP concentrations are not affected by hepatic dysfunction. *BMC Nephrology* 2018; **19**(1).
175. Aravinthan AD, Barbas AS, Doyle AC, et al. Characteristics of liver transplant candidates delisted following recompensation and predictors of such delisting in alcohol-related liver disease: a case–control study. *Transplant International* 2017; **30**(11): 1140-9.

176. Sharma SA, Kowgier M, Hansen BE, et al. Toronto HCC risk index: A validated scoring system to predict 10-year risk of HCC in patients with cirrhosis. *Journal of hepatology* 2017.
177. Sy E, Ronco JJ, Searle R, Karvellas CJ. Prognostication of critically ill patients with acute-on-chronic liver failure using the Chronic Liver Failure-Sequential Organ Failure Assessment: A Canadian retrospective study. *Journal of critical care* 2016; **36**: 234-9.
178. Buczkowski AK, Schaeffer DF, Kim PTW, et al. Spatulated end-to-end bile duct reconstruction in orthotopic liver transplantation. *Clinical Transplantation* 2007; **21**(1): 7-12.
179. Sobotka LA, Mumtaz K, Hinton A, et al. Cannabis use may reduce healthcare utilization and improve hospital outcomes in patients with cirrhosis. Annals of Hepatology; 2021.
180. Khan AA, Hadi YB, Thompson JM, Kupec JT. Acute kidney injury after multiphase imaging for lesions detected on hepatocellular carcinoma surveillance in patients with cirrhosis. *Bmj Open Gastroenterology* 2020; **7**(1).
181. Wang S, Toy M, Hang Pham TT, So S. Causes and trends in liver disease and hepatocellular carcinoma among men and women who received liver transplants in the U.S., 2010-2019. *PloS one* 2020; **15**(9 September).
182. Danforth D, Gabriel RA, Clark AI, et al. Preoperative risk factors for massive transfusion, prolonged ventilation requirements and mortality in patients undergoing liver transplantation. *Korean journal of anesthesiology* 2019.
183. Kim D, Cholankeril G, Li AA, et al. Trends in hospitalizations for chronic liver disease-related liver failure in the United States, 2005-2014. *Liver International* 2019; **39**(9): 1661-71.
184. Sunjaya DB, Lennon RJ, Shah VH, Kamath PS, Simonetto DA. Prevalence and Predictors of Third-Generation Cephalosporin Resistance in the Empirical Treatment of Spontaneous Bacterial Peritonitis. *Mayo Clinic Proceedings* 2019; **94**(8): 1499-508.
185. Al-Azzawi Y, Spaho L, Mahmoud M, Kheder J, Foley A, Cave D. Video Capsule Endoscopy in the Assessment of Portal Hypertensive Enteropathy. *International journal of hepatology* 2018; **2018**: 5109689.
186. Cullaro G, Park M, Lai JC. "Normal" Creatinine Levels Predict Persistent Kidney Injury and Waitlist Mortality in Outpatients With Cirrhosis. *Hepatology* 2018; **68**(5): 1953-60.
187. Jacobs JP, Dong TS, Agopian V, et al. Microbiome and bile acid profiles in duodenal aspirates from patients with liver cirrhosis: The Microbiome, Microbial Markers and Liver Disease Study. *Hepatology research : the official journal of the Japan Society of Hepatology* 2018; **48**(13): 1108-17.
188. Sandrasegaran K, Cui E, Elkady R, et al. Can functional parameters from hepatobiliary phase of gadoxetate MRI predict clinical outcomes in patients with cirrhosis? *European Radiology* 2018; **28**(10): 4215-24.
189. Singal AG, Tiro J, Li X, Adams-Huet B, Chubak J. Hepatocellular Carcinoma Surveillance Among Patients With Cirrhosis in a Population-based Integrated Health Care Delivery System. *Journal of clinical gastroenterology* 2017; **51**(7): 650-5.
190. Tavakoli H, Robinson A, Liu B, et al. Cirrhosis Patients with Nonalcoholic Steatohepatitis Are Significantly Less Likely to Receive Surveillance for Hepatocellular Carcinoma. *Digestive diseases and sciences* 2017; **62**(8): 2174-81.
191. Yang JD, Dai J, Singal AG, et al. Improved Performance of Serum Alpha-Fetoprotein for Hepatocellular Carcinoma Diagnosis in HCV Cirrhosis with Normal Alanine Transaminase. *Cancer epidemiology, biomarkers & prevention : a publication of the American Association for Cancer Research, cosponsored by the American Society of Preventive Oncology* 2017; **26**(7): 1085-92.
192. Allegretti AS, Ortiz G, Kalim S, et al. Siglec-7 as a Novel Biomarker to Predict Mortality in Decompensated Cirrhosis and Acute Kidney Injury. *Digestive diseases and sciences* 2016; **61**(12): 3609-20.
193. Yang JD, Mohamed HA, Cvinar JL, Gores GJ, Roberts LR, Kim WR. Diabetes Mellitus Heightens the Risk of Hepatocellular Carcinoma Except in Patients With Hepatitis C Cirrhosis. *Am J Gastroenterol* 2016; **111**(11): 1573-80.
194. Berry K, Taylor J, Liou IW, Ioannou GN. Portal vein thrombosis is not associated with increased mortality among patients with cirrhosis. *Clinical Gastroenterology and Hepatology* 2015; **13**(3): 585-93.
195. Civan JM, Lindenmeyer CC, Whitsett M, Herrine SK. A Clinical Decision Rule Based on the AST-to-Platelet Ratio Index Improves Adherence to Published Guidelines on the Management of Acute Variceal Bleeding. *Journal of Clinical Gastroenterology* 2015; **49**(7): 599-606.
196. Gopal P, Yopp AC, Waljee AK, et al. Factors that affect accuracy of alpha-fetoprotein test in detection of hepatocellular carcinoma in patients with cirrhosis. *Clinical gastroenterology and hepatology : the official clinical practice journal of the American Gastroenterological Association* 2014; **12**(5): 870-7.
197. Wong RJ, Chou C, Bonham CA, Concepcion W, Esquivel CO, Ahmed A. Improved survival outcomes in patients with non-alcoholic steatohepatitis and alcoholic liver disease following liver transplantation: An analysis of 2002-2012 United Network for Organ Sharing data. *Clinical Transplantation* 2014; **28**(6): 713-21.
198. Xiao J, Zhao Y, Varghese RS, et al. Evaluation of metabolite biomarkers for hepatocellular carcinoma through stratified analysis by gender, race, and alcoholic cirrhosis. *Cancer Epidemiology Biomarkers and Prevention* 2014; **23**(1): 64-72.

199. Singal AG, Mukherjee A, Elmunzer BJ, et al. Machine learning algorithms outperform conventional regression models in predicting development of hepatocellular carcinoma. *Am J Gastroenterol* 2013; **108**(11): 1723-30.
200. Stepanova M, Wai H, Saab S, Mishra A, Venkatesan C, Younossi ZM. The portrait of an adult liver transplant recipient in the United States from 1987 to 2013. *JAMA internal medicine* 2014; **174**(8): 1407-9.
201. Barney EJ, Little EC, Gerkin RD, et al. Coated transjugular intrahepatic portosystemic shunt does not improve thrombocytopenia in patients with liver cirrhosis. *Digestive Diseases and Sciences* 2012; **57**(9): 2430-7.
202. Sundaram V, Jones DT, Shah NH, et al. Posttransplant Biliary Complications in the Pre- and Post-Model for End-Stage Liver Disease Era. *Liver Transplantation* 2011; **17**(4): 428-35.
203. Bell BP, Manos MM, Zaman A, et al. The epidemiology of newly diagnosed chronic liver disease in gastroenterology practices in the United States: Results from population-based surveillance. *American Journal of Gastroenterology* 2008; **103**(11): 2727-36.
204. Carey EJ, Gautam M, Ingall T, Douglas DD. The effect of liver transplantation on autonomic dysfunction in patients with end-stage liver disease. *Liver Transpl* 2008; **14**(2): 235-9.
205. Durante AJ, Meek JI, St Louis T, Navarro VJ, Sofair AN. Quantifying the burden of chronic viral hepatitis-related cirrhosis hospitalizations in New Haven County, Connecticut. *Connecticut medicine* 2008; **72**(7): 393-7.
206. Cerfolio RJ, Bryant AS. Efficacy of video-assisted thoracoscopic surgery with talc pleurodesis for porous diaphragm syndrome in patients with refractory hepatic hydrothorax. *The Annals of thoracic surgery* 2006; **82**(2): 457-9.
207. Kane L, Kahaleh M, Shami VM, et al. Comparison of the Grading of esophageal varices by transnasal endoluminal ultrasound and esophagogastroduodenoscopy. *Clinical Gastroenterology and Hepatology* 2005; **3**(8): 806-10.
208. Fu KA, DiNorcia J, Sher L, et al. Predictive factors of neurological complications and one-month mortality after liver transplantation. *Frontiers in Neurology* 2014; **5**(DEC).
209. McTaggart RA, Terrault NA, Vardanian AJ, Bostrom A, Feng S. Hepatitis C etiology of liver disease is strongly associated with early acute rejection following liver transplantation. *Liver Transpl* 2004; **10**(8): 975-85.
210. Wells CD, Murrill WB, Arguedas MR. Comparison of health-related quality of life preferences between physicians and cirrhotic patients: implications for cost-utility analyses in chronic liver disease. *Digestive diseases and sciences* 2004; **49**(3): 453-8.
211. Arguedas MR, McGuire BM, Fallon MB. Implementation of vaccination in patients with cirrhosis. *Digestive Diseases and Sciences* 2002; **47**(2): 384-7.
212. Peterson MS, Baron RL, Marsh JW, Jr., Oliver JH, 3rd, Confer SR, Hunt LE. Pretransplantation surveillance for possible hepatocellular carcinoma in patients with cirrhosis: epidemiology and CT-based tumor detection rate in 430 cases with surgical pathologic correlation. *Radiology* 2000; **217**(3): 743-9.
213. Zaman A, Hapke R, Flora K, Rosen H, Benner K. Prevalence of upper and lower gastrointestinal tract findings in liver transplant candidates undergoing screening endoscopic evaluation. *American Journal of Gastroenterology* 1999; **94**(4): 895-9.
214. Eigenbrodt ML, McCashland TM, Dy RM, Clark J, Galati J. Heterozygous alpha 1-antitrypsin phenotypes in patients with end stage liver disease. *Am J Gastroenterol* 1997; **92**(4): 602-7.
215. Haque MN, Al-Mahtab M, Das DC, et al. Effect of Granulocyte Colony-stimulating Factor and Erythropoietin on Patients with Acute-on-chronic Liver Failure. *Euroasian J Hepatogastroenterol* 2020; **10**(2): 64-7.
216. Kabir MA, Chowdhury J, Bari MA, Bodruddoza K, Saha AK, Alam SB. Detection of precipitating factors of hepatic encephalopathy in chronic liver disease patients in a tertiary hospital. *Journal of Medicine (Bangladesh)* 2018; **19**(1): 10-4.
217. Gupta T, Goyal S. Paradigm shift in etiology of upper gastrointestinal bleed in emergency department. *Journal of Renal and Hepatic Disorders* 2021; **5**(1): 14-8.
218. Acharya G, Kaushik RM, Gupta R, Kaushik R. Child-Turcotte-Pugh Score, MELD Score and MELD-Na Score as Predictors of Short-Term Mortality among Patients with End-Stage Liver Disease in Northern India. *Inflammatory Intestinal Diseases* 2020; **5**(1): 1-10.
219. Choudhary NS, Sonavane A, Saraf N, et al. Poor Performance Status Predicts Mortality After Living Donor Liver Transplantation. *Journal of Clinical and Experimental Hepatology* 2019.
220. Jain M, Venkataraman J, Varghese J, Vij M, Reddy MS, Rela M. Explant liver evaluation decodes the mystery of cryptogenic cirrhosis! *JGH Open* 2019.
221. Jindal A, Mukund A, Kumar G, Sarin SK. Efficacy and safety of transjugular intrahepatic portosystemic shunt in difficult-to-manage hydrothorax in cirrhosis. *Liver International* 2019; **39**(11): 2164-73.
222. Maitra T, Das SP, Barman P, Deka J. Spectrum of complications of chronic liver disease in gauhati medical college and hospital: A hospital based study. *Journal of Clinical and Diagnostic Research* 2019; **13**(6): OC07-OC12.
223. Rajender A, Mathur S, Choudhary P, et al. Restless leg syndrome a common undiagnosed comorbidity of clinical significance in cirrhosis. *Gastroenterology and Hepatology from Bed to Bench* 2019; **12**(1): 13-6.

224. Shah ND, Ventura-Cots M, Abraldes JG, et al. Alcohol-Related Liver Disease Is Rarely Detected at Early Stages Compared With Liver Diseases of Other Etiologies Worldwide. *Clinical gastroenterology and hepatology : the official clinical practice journal of the American Gastroenterological Association* 2019; **17**(11): 2320-9.e12.
225. Bansal N, Vij V, Rastogi M, Wadhawan M, Kumar A. Retrospective analysis of explants liver pathology: Experience from a tertiary care center in India. *Indian Journal of Transplantation* 2018; **12**(1): 41-7.
226. Prerna G, Omesh G, Deepinder K, Rajoo SC. Etiological Profile of Cirrhosis in a Tertiary Care Institute in Northern India. *Journal of Gastrointestinal Infections* 2018; **8**(1): 28-31.
227. Verma N, Kaur A, Sharma R, et al. Outcomes after multiple courses of granulocyte colony-stimulating factor and growth hormone in decompensated cirrhosis: A randomized trial. *Hepatology* 2018; **68**(4): 1559-73.
228. Yadav SK, Saigal S, Choudhary NS, et al. Cytomegalovirus infection in living donor liver transplant recipients significantly impacts the early post-transplant outcome: A single center experience. *Transplant infectious disease : an official journal of the Transplantation Society* 2018; **20**(4): e12905.
229. Gupta T, Dhiman RK, Rathi S, et al. Impact of Hepatic and Extrahepatic Insults on the Outcome of Acute-on-Chronic Liver Failure. *Journal of clinical and experimental hepatology* 2017; **7**(1): 9-15.
230. Mukherjee PS, Vishnubhatla S, Amarapurkar DN, et al. Etiology and mode of presentation of chronic liver diseases in India: A multi centric study. *PloS one* 2017; **12**(10): e0187033.
231. Goel A, Jat SL, Sasi A, Paliwal VK, Aggarwal R. Prevalence, severity, and impact on quality of life of restless leg syndrome in patients with liver cirrhosis in India. *Indian journal of gastroenterology : official journal of the Indian Society of Gastroenterology* 2016; **35**(3): 216-21.
232. Shalimar, Kumar D, Vadiraja PK, et al. Acute on chronic liver failure because of acute hepatic insults: Etiologies, course, extrahepatic organ failure and predictors of mortality. *Journal of gastroenterology and hepatology* 2016; **31**(4): 856-64.
233. Sharma B, Marwah R, Raina S, Sharma N, Kaushik M, Kaushal SS. A study on the etiology of cirrhosis of liver in adults living in the Hills of Himachal Pradesh, India. *Tropical gastroenterology : official journal of the Digestive Diseases Foundation* 2016; **37**(1): 37-41.
234. Amarapurkar D, Amarapurkar A. Indications of Liver Biopsy in the Era of Noninvasive Assessment of Liver Fibrosis. *Journal of clinical and experimental hepatology* 2015; **5**(4): 314-9.
235. Paul K, Kaur J, Kazal HL. To study the incidence, predictive factors and clinical outcome of spontaneous bacterial peritonitis in patients of cirrhosis with ascites. *Journal of Clinical and Diagnostic Research* 2015; **9**(7): 9-12.
236. Sharma P, Bansal R, Matin A, et al. Role of Transient Elastography (Fibroscan) in Differentiating Severe Acute Hepatitis and Acute on Chronic Liver Failure. *Journal of clinical and experimental hepatology* 2015; **5**(4): 303-9.
237. Maiwall R, Kumar S, Chaudhary AK, et al. Serum ferritin predicts early mortality in patients with decompensated cirrhosis. *Journal of Hepatology* 2014; **61**(1): 43-50.
238. Ray G. Trends of chronic liver disease in a tertiary care referral hospital in Eastern India. *Indian journal of public health* 2014; **58**(3): 186-94.
239. Nayak NC, Jain D, Vasdev N, Gulwani H, Saigal S, Soin A. Etiologic types of end-stage chronic liver disease in adults: analysis of prevalence and their temporal changes from a study on native liver explants. *European journal of gastroenterology & hepatology* 2012; **24**(10): 1199-208.
240. Chawla YK, Kashinath RC, Duseja A, Dhiman RK. Predicting Mortality Across a Broad Spectrum of Liver Disease-An Assessment of Model for End-Stage Liver Disease (MELD), Child-Turcotte-Pugh (CTP), and Creatinine-Modified CTP Scores. *Journal of clinical and experimental hepatology* 2011; **1**(3): 161-8.
241. Cherian JV, Deepak N, Ponnusamy RP, Somasundaram A, Jayanthi V. Non-invasive predictors of esophageal varices. *Saudi Journal of Gastroenterology* 2011; **17**(1): 64-8.
242. Jain S, Agarwal S, Tamhankar P, Verma P, Choudhuri G. Lack of association of primary iron overload and common HFE gene mutations with liver cirrhosis in adult Indian population. *Indian journal of gastroenterology : official journal of the Indian Society of Gastroenterology* 2011; **30**(4): 161-5.
243. Mohan P, Venkataraman J. Prevalence and risk factors for unsuspected spontaneous ascitic fluid infection in cirrhotics undergoing therapeutic paracentesis in an outpatient clinic. *Indian journal of gastroenterology : official journal of the Indian Society of Gastroenterology* 2011; **30**(5): 221-4.
244. Pazhanivel M, Jayanthi V. Diabetes mellitus and cirrhosis liver. *Minerva gastroenterologica e dietologica* 2010; **56**(1): 7-11.
245. Sahu P, Kar SK, Mohapatra A, Dwibedi B, Singh SP. Seroepidemiological study of hepatitis B and C viral infection in acute and chronic hepatitis patients in a hospital setup in Orissa. *Journal of Pure and Applied Microbiology* 2010; **4**(1): 251-5.
246. Sharma B, Srinivasan R, Chawla YK, et al. Clinical utility of prothrombin induced by vitamin K absence in the detection of hepatocellular carcinoma in Indian population. *Hepatology International* 2010; **4**(3): 569-76.
247. Jain M, Chakravarti A, Kar P. Clinical significance of isolated anti-hbc positivity in cases of chronic liver disease in new delhi, India. *Journal of global infectious diseases* 2009; **1**(1): 29-32.

248. Irshad M, Dhar I, Khushboo, Singh S, Kapoor S. Comparison of serological and nucleic Acid based assays used to diagnose hepatitis C virus (HCV) infection in acute and chronic liver diseases. *International journal of health sciences* 2007; **1**(1): 3-10.
249. Joshi N, Rao S, Kumar A, Patil S, Rani S. Hepatitis A vaccination in chronic liver disease: is it really required in a tropical country like India? *Indian journal of medical microbiology* 2007; **25**(2): 137-9.
250. Paul SB, Sreenivas V, Gulati MS, et al. Incidence of hepatocellular carcinoma among Indian patients with cirrhosis of liver: an experience from a tertiary care center in northern India. *Indian journal of gastroenterology : official journal of the Indian Society of Gastroenterology* 2007; **26**(6): 274-8.
251. Agarwal N, Naik S, Aggarwal R, et al. Occult hepatitis B virus infection as a cause of cirrhosis of liver in a region with intermediate endemicity. *Indian journal of gastroenterology : official journal of the Indian Society of Gastroenterology* 2003; **22**(4): 127-31.
252. Borse N, Sawant P, Gala B. Assessment of renal and hepatic hemodynamics in cirrhosis of liver. *Indian Journal of Gastroenterology* 2002; **21**(6): 213-5.
253. Irshad M, Joshi YK, Choudhary PS. Etiological spectrum of viral hepatitis in various forms of liver diseases in Delhi, India. *International Medical Journal* 2001; **8**(2): 97-100.
254. Ray G, Ghoshal UC, Banerjee PK, et al. Aetiological spectrum of chronic liver disease in eastern India. *Tropical gastroenterology : official journal of the Digestive Diseases Foundation* 2000; **21**(2): 60-2.
255. Das U, Kar P, Gopalkrishna V, Sharma JK, Madan K, Das BC. Comparative evaluation of hepatitis C virus infection in serum and liver tissue of patients with chronic liver disease by reverse transcription-polymerase chain reaction. *Clinical microbiology and infection : the official publication of the European Society of Clinical Microbiology and Infectious Diseases* 1999; **5**(5): 256-61.
256. Sawant P, Rathi PM, Upadhyaya A. Hepatitis B subtypes and hepatitis C genotypes in cirrhosis in western India: results of a pilot study. *The Journal of the Association of Physicians of India* 1999; **47**(6): 580-3.
257. Berry N, Chakravarti A, Kar P, Das BC, Santhanam, Mathur MD. Association of hepatitis C virus & hepatitis B virus in chronic liver disease. *The Indian journal of medical research* 1998; **108**: 255-9.
258. Panigrahi AK, Panda SK, Dixit RK, et al. Magnitude of hepatitis C virus infection in India: prevalence in healthy blood donors, acute and chronic liver diseases. *Journal of medical virology* 1997; **51**(3): 167-74.
259. Adib A, Ghayumi SMA, Fallahi MJ, Arasteh P. Evaluation of high oxygen saturation status in cirrhotic patients. *Shiraz E Medical Journal* 2020; **21**(4): 1-5.
260. Hatami B, Ashtari S, Sharifian A, et al. Changing the cause of liver cirrhosis from hepatitis B virus to fatty liver in Iranian patients. *Gastroenterology and hepatology from bed to bench* 2017; **10**(Suppl1): S20-s6.
261. Pourafkari L, Ghaffari S, Nazeri L, et al. Electrocardiographic findings in hepatic cirrhosis and their association with the severity of disease. *Cor et Vasa* 2017; **59**(2): e105-e13.
262. Jafarian A, Kasraianfard A, Najafi A, et al. Patient outcomes in a liver transplant program in Iran. *Experimental and Clinical Transplantation* 2014; **12**(SUPPL. 1): 86-91.
263. Karimi MH, Hejr S, Geramizadeh B, Nikeghbalian S, Kamali-Sarvestani E, Yaghobi R. Study of the relationships between IL-23R, IL-17, IL-21 polymorphisms and serum level of IL-17, IL-21 with acute graft rejection in iranian liver transplant recipients. *Immunological Investigations* 2014; **43**(1): 69-85.
264. Abedian S, Asl Soleimani H, Saberifiroozi M, Malekzadeh R. Common Digestive and Liver Diseases among 5880 Patients Admitted to Shariati Hospital,Tehran,Iran during 2000-2009. *Middle East journal of digestive diseases* 2012; **4**(1): 28-33.
265. Khoshbaten M, Rostami Nejad M, Ansarin K, et al. The association between clinical symptoms, laboratory findings and serum endothelin 1 concentrations, in cirrhotic patients with and without hepatopulmonary syndrome. *Gastroenterology and hepatology from bed to bench* 2012; **5**(Suppl 1): S13-9.
266. Rahimkhani M, Ghofrani H. HELICOBACTER PYLORI AND PEPTIC ULCER IN CIRRHOTIC PATIENTS. *Pakistan Journal of Medical Sciences* 2008; **24**(6): 849-52.
267. Tarzamni MK, Somi MH, Farhang S, Jalilvand M. Portal hemodynamics as predictors of high risk esophageal varices in cirrhotic patients. *World Journal of Gastroenterology* 2008; **14**(12): 1898-902.
268. Baimakhanov Z, Kaniev S, Doskhanov M, et al. Evolution of Liver Transplantation in Kazakhstan: Two-Era Experience of a Single Center, the First Report. Transplantation proceedings. 2019/11/18 ed; 2019. p. 3360-3.
269. Purbey BK, Gurung RB, Panday R, Acharya B, Mehta RK. The Etiology of Upper Gastrointestinal Bleeding in Patients with Liver Cirrhosis in Dhulikhel Hospital. *Kathmandu University medical journal (KUMJ)* 2017; **15**(60): 292-5.
270. K.C S, Sharma D, Khadka S, et al. Prevalence of Gall Stones in Liver Cirrhosis. *JNMA; journal of the Nepal Medical Association* 2015; **53**(200): 275-9.
271. Shrestha SM, Shrestha S, Shrestha A, et al. High prevalence of hepatitis B virus infection and inferior vena cava obstruction among patients with liver cirrhosis or hepatocellular carcinoma in Nepal. *Journal of gastroenterology and hepatology* 2007; **22**(11): 1921-8.

272. Abro HA, Shaikh BA, Mugheri AH, Ansari IA, Shaikh ZA, Kumar A. Proportion of nonalcoholic steatohepatitis in patients with chronic liver disease. *Pakistan Journal of Medical and Health Sciences* 2021; **15**(6): 1272-4.
273. Bhatti AA, Khan MF, Bhatti HUR, Punshi A. Frequency of portal vein thrombosis in patients with liver cirrhosis. *Pakistan Journal of Medical and Health Sciences* 2021; **15**(4): 887-8.
274. Alvi H, Zuberi BF, Rasheed T, Ibrahim MA. Evaluation of endoscopic variceal band ligation sessions in obliteration of esophageal varices. *Pakistan Journal of Medical Sciences* 2020; **36**(2): 37-41.
275. Muhammad N, Khan MH, Jawaid HA, Ali MO, Shah SMA, Saeed S. Etiology of liver cirrhosis in district buner, khyber pakhtunkhwa. *Journal of Medical Sciences (Peshawar)* 2020; **28**(4): 364-6.
276. Naqvi IH, Ahmed J, Salim M, et al. Restless Leg syndrome in patients with liver cirrhosis! Its frequency, severity, and correlation. *CNS & neurological disorders drug targets* 2020.
277. Javaid AH, Anjum W, Gull S. ANALYSIS OF FREQUENCY OF RE-BLEEDING AFTER SUCCESSFUL ENDOSCOPIC MANAGEMENT OF ESOPHAGEAL VARICES IN CIRRHOTIC PATIENTS. *Indo American Journal of Pharmaceutical Sciences* 2019; **6**(2): 4311-4.
278. Kirplani PD, Qadar LT, Ochani RK, et al. Recognition of Antibiotic Resistance in Spontaneous Bacterial Peritonitis Caused by Escherichia coli in Liver Cirrhotic Patients in Civil Hospital Karachi. *Cureus* 2019; **11**(7): e5284.
279. Iqbal J, Khalid MA, Hanif FM, et al. Correlation between MELD and UNa/K ratio in predicting renal dysfunction in cirrhotic patients. *Journal of Translational Internal Medicine* 2018; **6**(4): 181-4.
280. Nawaz F, Nawaz R, Sadiq F. Common causes of hepatic encephalopathy at Govt. Teaching Hospital Shahdara. *Pakistan Journal of Medical and Health Sciences* 2018; **12**(4): 1497-9.
281. Fayyaz M, Rana R, Iftikhar T, Makki MU. Important mortality predictors in patients of decompensated chronic liver disease presenting to a Tertiary Care Hospital of Lahore. *Pakistan Journal of Medical and Health Sciences* 2017; **11**(4): 1377-9.
282. Kausar R, Manzoor F, Ahmed M. To determine the frequency of Vitamin D deficiency in patients with liver cirhosis. *Medical Forum Monthly* 2017; **28**(4): 18-21.
283. Laeeq SM, Hanif FM, Luck NH, Mandhwani RK, Iqbal J, Mehdi SH. Living-Donor Liver Transplant Follow-Up: A SingleCenter Experience. *Experimental and clinical transplantation : official journal of the Middle East Society for Organ Transplantation* 2017; **15**(Suppl 1): 254-7.
284. Parkash O, Mohyuddin GR, Ayub A, Nazir I, Maan AA, Hamid S. Electrophysiological changes in patients with liver cirrhosis in a tertiary care hospital in Karachi, Pakistan. *Journal of Ayub Medical College, Abbottabad : JAMC* 2016; **28**(4): 676-9.
285. Salim A, Malik K, Farooq MO, Butt U, Butt AK, Alam A. Early Initiation Of Beta Blockers Following Primary Endoscopic Therapy For Bleeding Oesophageal Varices In Cirrhotics. *Journal of Ayub Medical College, Abbottabad : JAMC* 2017; **29**(2): 186-9.
286. Umer N, Makki MU, Kiran SK, Jadoon NA. Serum ferritin as a predictor of 30 days mortality in Patients of decompensated chronic liver disease. *Journal of Ayub Medical College, Abbottabad : JAMC* 2017; **29**(3): 415-8.
287. Dar FS, Zia H, Hafeez Bhatti AB, et al. Short Term Donor Outcomes After Hepatectomy in Living Donor Liver Transplantation. *Journal of the College of Physicians and Surgeons--Pakistan : JCPSP* 2016; **26**(4): 272-6.
288. Fareed W, Tauseef A, Wasay S, Alam T, Altaf R, Nawaz Z. Etiology of cirrhosis among adults in Karachi-perspective from three tertiary care hospitals. *Rawal Medical Journal* 2016; **41**(3): 277-9.
289. Khan A, Ayub M, Khan WM. Hyperammonemia Is Associated with Increasing Severity of Both Liver Cirrhosis and Hepatic Encephalopathy. *International Journal of Hepatology* 2016; **2016**.
290. Zuberi BF, Alvi H, Zuberi FF, Rasheed T, Nawaz Z, Fatima tuz Z. Frequency of minimal hepatic encepalopathy in illeterate patients with compensated cirrhosis. *Pakistan Journal of Medical Sciences* 2016; **32**(3): 595-8.
291. Hussain W, Khalid AB, Usmani T, Ghufran A, Shah H. Low dose albumin for the prevention of renal impairment following large volume paracentesis in cirrhosis. *Pakistan Journal of Medical Sciences* 2015; **31**(3): 592-6.
292. Abbas Z, Yakoob J, Usman MW, Shakir T, Hamid S, Jafri W. Effect of Helicobacter pylori and its virulence factors on portal hypertensive gastropathy and interleukin (IL)-8, IL-10, and tumor necrosis factor-alpha levels. *Saudi Journal of Gastroenterology* 2014; **20**(2): 120-7.
293. Shah HA, Azam Z, Rauf J, et al. Carvedilol vs. esophageal variceal band ligation in the primary prophylaxis of variceal hemorrhage: A multicentre randomized controlled trial. *Journal of Hepatology* 2014; **60**(4): 757-64.
294. Butt Z, Jadoon NA, Salaria ON, et al. Diabetes mellitus and decompensated cirrhosis: Risk of hepatic encephalopathy in different age groups. *Journal of Diabetes* 2013; **5**(4): 449-55.
295. Mehmood MA, Waseem T, Ahmad FZ, Humayun MA. Measuring partial pressure of ammonia in arterial or venous blood vs total ammonia levels in hepatic encephalopathy. *Journal of Gastroenterology and Hepatology Research* 2013; **2**(5): 602-6.
296. Haq MM, Faisal N, Khalil A, Haqqi SAH, Shaikh H, Arain N. Midazolam for sedation during diagnostic or therapeutic upper gastrointestinal endoscopy in cirrhotic patients. *European Journal of Gastroenterology and Hepatology* 2012; **24**(10): 1214-8.

297. Phulpoto JA, Shah IA, Bhatti Z. Prevalence of hepatocellular carcinoma in cirrhotic patients of Northern Sindh attending liver clinics at Ghulam Mohammad Mahar Medical College Hospitals Sukkur and Khairpur. *Journal of the Liaquat University of Medical and Health Sciences* 2012; **11**(1): 29-33.
298. Zainab S, Qureshi HJ, Bukhari SMR. Study of etiology and prevalence of esophageal varices in patients of liver cirrhosis. *Pakistan Journal of Medical and Health Sciences* 2012; **6**(2): 459-62.
299. Abbasi A, Bhutto AR, Butt N, Munir SM, Dhillo AK. Frequency of portal hypertensive gastropathy and its relationship with biochemical, haematological and endoscopic features in cirrhosis. *Journal of the College of Physicians and Surgeons--Pakistan : JCPSP* 2011; **21**(12): 723-6.
300. Ahmed W, Qureshi H, Arif A, Alam SE. Changing trend of viral hepatitis--"A twenty one year report from Pakistan Medical Research Council Research Centre, Jinnah Postgraduate Medical Centre, Karachi". *JPMA The Journal of the Pakistan Medical Association* 2010; **60**(2): 86-9.
301. Khan MR, Kassi M, Janjua SA. Abdominal wall hernia repair in cirrhotic patients: Outcomes seen at a tertiary care hospital in a developing country. *Tropical Doctor* 2010; **40**(1): 5-8.
302. Khan TM, Ali A, Iman NU. Yield of ascitic fluid cultures in spontaneous bacterial peritonitis in cirrhosis. *Journal of Medical Sciences* 2010; **18**(1): 59-62.
303. Khan P, Ahmad A, Muhammad N, Khan TM, Ahmad B. Screening of 110 cirrhotic patients for hepatitis B and C at Saidu Teaching Hospital Saidu Sharif Swat. *Journal of Ayub Medical College, Abbottabad : JAMC* 2009; **21**(1): 119-21.
304. Memon MS, Burney A, Ghani MH, et al. Frequency of hepatic hydrothorax in patients with liver cirrhosis: A tertiary care hospital experience. *Journal of the Liaquat University of Medical and Health Sciences* 2009; **8**(2): 106-8.
305. Soomro AA, Devrajani BR, Shaikh K, Shah SZA, Devrajani T, Bibi I. Serum zinc level in patients with liver cirrhosis. *Pakistan Journal of Medical Sciences* 2009; **25**(6): 986-91.
306. Ali M, Abbas SZ, Sultana F, Akhtar W, Shaw S, Abbas SQ. Non-B, non-C hepatitis as a cause of advanced chronic liver disease requiring medical admission at a rural centre in Pakistan. *Pakistan Journal of Medical Sciences* 2008; **24**(2): 278-82.
307. Almani SA, Memon AS, Memon AI, Shah MI, Rahpoto MQ, Solangi R. Cirrhosis of liver: Etiological factors, complications and prognosis. *Journal of the Liaquat University of Medical and Health Sciences* 2008; **7**(2): 61-6.
308. Rahopoto MQ, Almani SA, Shaikh MA, et al. Frequency of night blindness in cirrhosis and effective restoration of vision with vitamin A therapy. *Journal of the Liaquat University of Medical and Health Sciences* 2008; **7**(2): 75-8.
309. Ismail FW, Mumtaz K, Shah HA, et al. Factors predicting in-hospital mortality in patients with cirrhosis hospitalized with gastro-esophageal variceal hemorrhage. *Indian Journal of Gastroenterology* 2006; **25**(5): 240-3.
310. Mashud I, Khan H, Khattak AM. Relative frequency of hepatitis B and C viruses in patients with hepatic cirrhosis at DHQ Teaching Hospital D. I. Khan. *Journal of Ayub Medical College, Abbottabad : JAMC* 2004; **16**(1): 32-4.
311. Bukhtiari N, Hussain T, Iqbal M, Malik AM, Qureshi AH, Hussain A. Hepatitis B and C single and co-infection in chronic liver disease and their effect on the disease pattern. *JPMA The Journal of the Pakistan Medical Association* 2003; **53**(4): 136-40.
312. Iqbal S, Ruknuddin A. Liver cirrhosis in North-West Frontier Province of Pakistan. *Journal of the College of Physicians and Surgeons Pakistan* 2002; **12**(5): 289-91.
313. Durrani AB, Rana AB, Siddiqi HS, Marwat BU. The spectrum of chronic liver disease in Balochistan. *Medical Forum Monthly* 2001; **12**(4): 20-2.
314. Farooqi JI, Farooqi RJ. Relative frequency of Hepatitis 'B' virus and Hepatitis 'C' virus infections in patients of cirrhosis in NWFP. *Journal of the College of Physicians and Surgeons Pakistan* 2000; **10**(6): 217-9.
315. Niriella MA, Kobbegala VJ, Karalliyadda HN, et al. Sero-prevalence and vaccination status of hepatitis A and hepatitis B among adults with cirrhosis in Sri Lanka: a hospital based cohort study. *BMC research notes* 2017; **10**(1): 303.
316. Tailakh MA, Poupko L, Kayyal N, et al. Liver Cirrhosis, Etiology and Clinical Characteristics Disparities Among Minority Population. *J Immigr Minor Health* 2021.
317. Abdeen SM, Olusi SO, Askar HA, Thalib L, Al-Azemi A, George S. The predictive value of CD38 positive hepatic stellate cell count for assessing disease activity and fibrosis in patients with chronic hepatitis. *Acta Histochemica* 2009; **111**(6): 520-30.
318. Elzouki AN, Suliman S, Alhasan R, Abdullah A, Othman M, Badi A. Predicting mortality of patients with cirrhosis admitted to medical intensive care unit: An experience of a single tertiary center. *Arab Journal of Gastroenterology* 2016; **17**(4): 159-63.
319. Kahn FY, Abbas MT, El Mudathir M, Errayes M, El Hiday AH. Clinical pattern of cirrhotic ascities in the state of Qatar. *Qatar Medical Journal* 2008; **17**(1): 46-50.
320. Enezi A, Al-Jahdali F, Ahmed AE, et al. Symptoms of Daytime Sleepiness and Sleep Apnea in Liver Cirrhosis Patients. *Annals of hepatology* 2017; **16**(4): 591-8.

321. Al-Harbi A, Abdullah K, Al-Abdulkareem A, Alghamdi A, Al-Jahdali H. Prevalence, severity, and prognostic effect of hepatopulmonary syndrome in liver transplant candidates. *Annals of Transplantation* 2016; **21**: 180-4.
322. Al-Hamoudi WK, Abdelrahman AA, Helmy A, et al. The role of Fibroscan in predicting the presence of varices in patients with cirrhosis. *European journal of gastroenterology & hepatology* 2015; **27**(11): 1307-12.
323. Singal AK, Ayoola AE. Prevalence and factors affecting occurrence of type 2 diabetes mellitus in Saudi patients with chronic liver disease. *Saudi journal of gastroenterology : official journal of the Saudi Gastroenterology Association* 2008; **14**(3): 118-21.
324. Idilman R, Aydogan M, Oruncu MB, et al. NATURAL HISTORY OF CIRRHOSIS: CHANGING TRENDS IN ETIOLOGY OVER THE YEARS. *Digestive diseases (Basel, Switzerland)* 2020.
325. Tokmak S, Harmanci Özakyol A. Elevated levels of fecal calprotectin in cirrhotic patients and spontaneous bacterial peritonitis. *Duzce Medical Journal* 2019; **21**(3): 214-7.
326. Atalan HK, Gucyetmez B, Aslan S, Yazar S, Polat KY. Postoperative acute kidney injury in living donor liver transplantation recipients. *International Journal of Artificial Organs* 2018; **41**(1): 37-42.
327. Sahin A, Artas H, Tunc N, Yalniz M, Bahcecioglu IH. Hematological indices in portal hypertension: Cirrhosis versus noncirrhotic portal hypertension. *Journal of Clinical Medicine* 2018; **7**(8).
328. Sahinturk Y, Cekin AH. Neutrophil-to-Lymphocyte Ratio as a Potential Early Marker of Antibiotic Resistance in Patients with Infected Cirrhotic Ascites. *Clinical Laboratory* 2018; **64**(9): 1403-11.
329. Balkan A, Alkan S, Barutçu S, et al. Etiological distribution and clinical features of cirrhotic patients: Single tertiary referral center experience. *Acta Medica Mediterranea* 2016; **32**(3): 669-74.
330. Biyik M, Ataseven H, Biyik Z, et al. KDIGO (Kidney Disease: Improving Global Outcomes) criteria as a predictor of hospital mortality in cirrhotic patients. *Turkish Journal of Gastroenterology* 2016; **27**(2): 173-9.
331. Cagin YF, Atayan Y, Erdogan MA, Dagtekin F, Colak C. Incidence and clinical presentation of portal vein thrombosis in cirrhotic patients. *Hepatobiliary and Pancreatic Diseases International* 2016; **15**(5): 499-503.
332. Kaya M, Baran Ş, Güya C, Kaplan MA. Prevalence and predictive factors for development of splenic artery aneurysms in cirrhosis. *Indian Journal of Gastroenterology* 2016; **35**(3): 201-6.
333. Bozbaş Ş S, Eroğlu S, Eyüboğlu FÖ, Moray G, Haberal M. Pulmonary hypertension improves after orthotopic liver transplant in patients with chronic liver disease. *Experimental and Clinical Transplantation* 2015; **13**: 115-9.
334. Topdagi O, Okcu N, Bilen N. The frequency of complications and the etiology of disease in patients with liver cirrhosis in erzurum. *The Eurasian journal of medicine* 2014; **46**(2): 110-4.
335. Celikbilek M, Selcuk H, Yilmaz U. The effect of hepatotropic virus (HBV-HCV) infections on tuberculin skin test in patients with cirrhosis. *The Turkish journal of gastroenterology : the official journal of Turkish Society of Gastroenterology* 2012; **23**(3): 234-8.
336. Bassullu N, Turkmen I, Yaprak O, et al. General evaluation of hepatectomy and hepatocellular carcinoma cases. *Turk patoloji dergisi* 2011; **27**(3): 221-9.
337. Tamsel S, Demirpolat G, Killi R, et al. Vascular complications after liver transplantation: Evaluation with Doppler US. *Abdominal Imaging* 2007; **32**(3): 339-47.
338. Curgunlu A, Vural P, Canbaz M, Erten N, Karan MA, Tascioglu C. Plasma nitrate/nitrite and endothelin-1 in patients with liver cirrhosis. *Journal of clinical laboratory analysis* 2005; **19**(5): 177-81.
339. Baskol M, Gursoy S, Baskol G, Ozbakir O, Guven K, Yucesoy M. Five days of ceftriaxone to treat culture negative neutrocytic ascites in cirrhotic patients. *Journal of clinical gastroenterology* 2003; **37**(5): 403-5.
340. Ozer B, Serin E, Yilmaz U, et al. Clinicopathologic features and risk factors for hepatocellular carcinoma: results from a single center in southern Turkey. *The Turkish journal of gastroenterology : the official journal of Turkish Society of Gastroenterology* 2003; **14**(2): 85-90.
341. Kaymakoglu S, Eraksoy H, Okten A, et al. Spontaneous ascitic infection in different cirrhotic groups: prevalence, risk factors and the efficacy of cefotaxime therapy. *European journal of gastroenterology & hepatology* 1997; **9**(1): 71-6.
342. Sezgin O, Bahar K, Uzunalimoglu O. Gallbladder wall thickening and emptying function in liver cirrhosis. *Turkish Journal of Gastroenterology* 1997; **8**(4): 397-401.
343. Thuring EG, Joller-Jemelka HI, Sareth H, Sokhan U, Reth C, Grob P. Prevalence of markers of hepatitis viruses A, B, C and of HIV in healthy individuals and patients of a Cambodian province. *The Southeast Asian journal of tropical medicine and public health* 1993; **24**(2): 239-49.
344. Handajani R, Soetjipto, Lusida MI, et al. Prevalence of GB virus C/Hepatitis G virus infection among various populations in Surabaya, Indonesia, and identification of novel groups of sequence variants. *Journal of clinical microbiology* 2000; **38**(2): 662-8.
345. Hadiwandowo S, Tsuda F, Okamoto H, et al. Hepatitis B virus subtypes and hepatitis C virus genotypes in patients with chronic liver disease or on maintenance hemodialysis in Indonesia. *Journal of medical virology* 1994; **43**(2): 182-6.
346. Ng YZ, Lai LL, Wong SW, Mohamad SY, Chuah KH, Chan WK. Attenuation parameter and liver stiffness measurement using FibroTouch vs Fibroscan in patients with chronic liver disease. *PloS one* 2021; **16**(5 May).

347. Wendy Tan AY, Chieng JY. Endoscopic variceal ligation as primary prophylaxis for oesophageal variceal bleeding at a Malaysian tertiary hospital. *The Medical journal of Malaysia* 2018; **73**(6): 361-4.
348. Nakai K, Win KM, Oo SS, Arakawa Y, Abe K. Molecular characteristic-based epidemiology of hepatitis B, C, and E viruses and GB virus C/hepatitis G virus in Myanmar. *Journal of clinical microbiology* 2001; **39**(4): 1536-9.
349. Chang PE, Tan CK, Cheah CC, Li W, Chow WC, Wong YJ. Validation of the Expanded Baveno-VI Criteria for Screening Gastroscopy in Asian Patients with Compensated Advanced Chronic Liver Disease. *Digestive Diseases and Sciences* 2021; **66**(4): 1343-8.
350. Chang PE, Hartono JL, Ngai YL, Dan YY, Lim KB, Chow WC. Optimal liver stiffness measurement values for the diagnosis of significant fibrosis and cirrhosis in chronic liver disease in Singapore. *Singapore medical journal* 2019; **60**(10): 532-7.
351. Wai CT, Woon WA, Tan YM, Lee KH, Tan KC. Pretransplant Model for End-stage Liver Disease score has no impact on posttransplant survival in living donor liver transplantation. *Transplantation proceedings* 2012; **44**(2): 396-8.
352. Suksamai A, Chaiprasert A, Chirapongsathorn S. Serum cystatin C as a predictor of 90-day mortality among patients admitted with complications of cirrhosis. *JGH Open* 2021; **5**(5): 607-13.
353. Nawalerspanya S, Sripongpun P, Chamroonkul N, Kongkamol C, Piratvisuth T. Validation of original, expanded Baveno VI, and stepwise & platelet-MELD criteria to rule out varices needing treatment in compensated cirrhosis from various etiologies. *Annals of Hepatology* 2020; **19**(2): 209-13.
354. Paocharern P, Sethasine S. Correlation of spleen stiffness by transient elastrography with parameters of indicated portal hypertension and the presence of esophageal varices. *Journal of the Medical Association of Thailand* 2018; **101**(4): S112-S8.
355. Piyachaturawat P, Siramolpiwat S, Sonsiri K, Tangkijvanich P, Treeprasertsuk S. Changes in transient elastography in early cirrhotic patients after receiving nonselective B-blocker for primary variceal bleeding prophylaxis: Three-month follow up. *JGH Open* 2018; **2**(5): 172-7.
356. Teerasarntipan T, Tiranathanagul K, Thanapirom K, et al. Clif-sofa and urine neutrophil gelatinase-associated lipocalin score for the diagnosis of acute-on-chronic liver failure and as a prognostic tool for mortality prediction. *Journal of the Medical Association of Thailand* 2018; **101**(11): 1487-94.
357. Asavakarn S, Sirivatanauksorn Y, Promraj R, et al. Systematic Pharmaceutical Educational Approach to Enhance Drug Adherence in Liver Transplant Recipients. *Transplantation proceedings* 2016; **48**(4): 1202-7.
358. Sirivatanauksorn Y, Dumronggittigule W, Limsrichamrern S, et al. Quality of life among liver transplantation patients. *Transplantation Proceedings* 2012; **44**(2): 532-8.
359. Sirivatanauksorn Y, Taweerutchana V, Limsrichamrern S, et al. Recipient and perioperative risk factors associated with liver transplant graft outcomes. *Transplantation Proceedings* 2012; **44**(2): 505-8.
360. Surawong A, Rojnuckarin P, Juntiang J, Akkawat B, Komolmit P, Intragumtornchai T. Hyperfibrinolysis and the risk of hemorrhage in stable cirrhotic patients. *Asian Biomedicine* 2010; **4**(2): 199-206.
361. Tangkijvanich P, Theamboonlers A, Hirsch P, Thongngam D, Kullavanijaya P, Poovorawan Y. Hepatitis viruses and chronic liver disease. *The Southeast Asian journal of tropical medicine and public health* 1999; **30**(3): 489-95.
362. Thong VD, Anh HTV. Prediction of esophageal varices based on serum-ascites albumin gradient in cirrhotic patients. *Gastroenterology Insights* 2021; **12**(2): 270-7.
363. Bui Huu H, Ha Thuc N, Thi Le HP, et al. Characterization of SCCA-IgM as a biomarker of liver disease in an Asian cohort of patients. *Scandinavian journal of clinical and laboratory investigation* 2018; **78**(3): 204-10.
364. Tran HT, Ushijima H, Quang VX, et al. Prevalence of hepatitis virus types B through E and genotypic distribution of HBV and HCV in Ho Chi Minh City, Vietnam. *Hepatology research : the official journal of the Japan Society of Hepatology* 2003; **26**(4): 275-80.
365. Bai DS, Zhou BH, Qian JJ, Zhang C, Jin SJ, Jiang GQ. Effects of laparoscopic splenectomy and azygoportal disconnection on liver synthesis function and cirrhosis: a 2-year prospective study. *Surgical Endoscopy* 2020; **34**(11): 5074-82.
366. Cao Z, Liu Y, Cai M, et al. The Use of NACSELD and EASL-CLIF Classification Systems of ACLF in the Prediction of Prognosis in Hospitalized Patients With Cirrhosis. *The American journal of gastroenterology* 2020; **115**(12): 2026-35.
367. Duan Z, Li L, Li J, Zhou S. Validation of the Combined Model Based on Platelet Count and Albumin to Rule out High-Risk Varices in Liver Cirrhosis. *BioMed Research International* 2020; **2020**.
368. Guan X, Xing F, Li Y. Alcohol consumption increases the incidence of hepatocellular carcinoma in patients with hepatitis B cirrhosis but not in patients with hepatitis C cirrhosis. *European journal of gastroenterology & hepatology* 2020.
369. Su H, Tong J, Liu X, et al. Characteristics and outcome of nosocomial bloodstream infection in patients with acute-on-chronic liver failure. *European Journal of Gastroenterology and Hepatology* 2020: 83-8.

370. Wu Y, Zhu Y, Feng Y, et al. Royal Free Hospital-Nutritional Prioritizing Tool improves the prediction of malnutrition risk outcomes in liver cirrhosis patients compared with Nutritional Risk Screening 2002. *British Journal of Nutrition* 2020; **124**(12): 1293-302.
371. Guo J, Shi J, Wang H, et al. Emerging gram-positive bacteria and drug resistance in cirrhosis patients with spontaneous bacterial peritonitis: A retrospective study. *Experimental and Therapeutic Medicine* 2019; **17**(6): 4568-76.
372. Hou JC, Zheng H, Qiang Z, et al. [Impact of psoas muscle index on early postoperative mortality and complications after liver transplantation]. *Zhonghua wai ke za zhi [Chinese journal of surgery]* 2018; **56**(5): 374-8.
373. Xu WP, Wang ZR, Zou X, et al. Serum Wisteria floribunda agglutinin-positive Mac-2-binding protein evaluates liver function and predicts prognosis in liver cirrhosis. *Journal of Digestive Diseases* 2018; **19**(4): 242-53.
374. Zhou XL, Mi YQ, Xu L, Liu YG, Zhong Y, Su ST. Clinical value of protein induced by vitamin K absence or antagonist-II in diagnosis of hepatocellular carcinoma. *World Chinese Journal of Digestology* 2018; **26**(7): 425-33.
375. Cheng Y, Huang LX, Zhang L, et al. Longitudinal intrinsic brain activity changes in cirrhotic patients before and one month after liver transplantation. *Korean Journal of Radiology* 2017; **18**(2): 370-7.
376. Jiang TT, Luo XP, Sun JM, Gao J. Clinical outcomes of transcatheter selective superior mesenteric artery urokinase infusion therapy vs transjugular intrahepatic portosystemic shunt in patients with cirrhosis and acute portal vein thrombosis. *World Journal of Gastroenterology* 2017; **23**(41): 7470-7.
377. Wang L, Feng YM, Ma XW, et al. Diagnostic efficacy of noninvasive liver fibrosis indexes in predicting portal hypertension in patients with cirrhosis. *PloS one* 2017; **12**(8).
378. Wang Y, Xin Z, Pan B, et al. Venous anastomosis using a non-penetrating vascular closure system in orthotopic liver transplantation. *Clinical Transplantation* 2017; **31**(12).
379. Qu J, Feng P, Luo Y, Lü X. Impact of hepatic function on serum procalcitonin for the diagnosis of bacterial infections in patients with chronic liver disease: A retrospective analysis of 324 cases. *Medicine (United States)* 2016; **95**(30).
380. Xiao Y, Ma C, Liu J, Li H, Zhang Z, Zhou S. Liver cirrhosis: Evaluation by using proper hepatic artery to splenic artery diameter ratio and Gd-EOB-DTPA-enhanced MR. *International Journal of Clinical and Experimental Medicine* 2016; **9**(5): 8039-46.
381. Zhao JC, Qi XS, Hou FF, et al. Prevalence, Risk Factors and In-hospital Outcomes of QTc Interval Prolongation in Liver Cirrhosis. *American Journal of the Medical Sciences* 2016; **352**(3): 285-95.
382. Zhao L, Winklhofer S, Yang Z, Wang K, He W. Optimal Adaptive Statistical Iterative Reconstruction Percentage in Dual-energy Monochromatic CT Portal Venography. *Academic radiology* 2016; **23**(3): 337-43.
383. Chen M, Zheng T, Han S, et al. A preliminary study of plasma cyclase-associated protein 2 as a novel biomarker for early stage and alpha-fetoprotein negative hepatocellular carcinoma patients. *Clinics and Research in Hepatology and Gastroenterology* 2015; **39**(2): 215-21.
384. Dai J, Qi X, Peng Y, et al. Association between D-dimer level and portal venous system thrombosis in liver cirrhosis: A retrospective observational study. *International Journal of Clinical and Experimental Medicine* 2015; **8**(9): 15296-301.
385. Shi R, Shen ZY, Teng da H, et al. Gallstones in liver transplant recipients: A single-center study in China. *The Turkish journal of gastroenterology : the official journal of Turkish Society of Gastroenterology* 2015; **26**(5): 429-34.
386. Xiong J, Wang J, Huang J, Sun W, Wang J, Chen D. Non-alcoholic steatohepatitis-related liver cirrhosis is increasing in China: a ten-year retrospective study. *Clinics (Sao Paulo, Brazil)* 2015; **70**(8): 563-8.
387. Chen HS, Trilok G, Wang F, Qi XL, Xiao JJ, Yang CQ. A single hospital study on portal vein thrombosis in cirrhotic patients - clinical characteristics & risk factors. *Indian Journal of Medical Research* 2014; **139**: 260-6.
388. Cheng Z, Li JW, Chen J, Fan YD, Guo P, Zheng SG. Therapeutic effects of laparoscopic splenectomy and esophagogastric devascularization on liver cirrhosis and portal hypertension in 204 cases. *Journal of Laparoendoscopic and Advanced Surgical Techniques* 2014; **24**(9): 612-6.
389. Ying X, Zhao Y, Wang JL, et al. Serum anti-osteopontin autoantibody as a novel diagnostic and prognostic biomarker in patients with hepatocellular carcinoma. *Oncology reports* 2014; **32**(4): 1550-6.
390. Bin Hu A, Wu LW, Tai Q, Zhu XF, He XS. Safety and efficacy of four steroid-minimization protocols in liver transplant recipients: 3-year follow-up in a single center. *Journal of Digestive Diseases* 2013; **14**(1): 38-44.
391. Song GJ, Feng B, Rao HY, Wei L. Etiological features of cirrhosis inpatients in Beijing, China. *Chinese medical journal* 2013; **126**(13): 2430-4.
392. You S, Rong Y, Zhu B, et al. Changing etiology of liver failure in 3,916 patients from northern China: a 10-year survey. *Hepatology international* 2013; **7**(2): 714-20.
393. Yu LL, Yin LY, Chen W, et al. [Prospective study on the relation between serum vitamin D levels and liver cirrhosis risk]. *Zhonghua gan zang bing za zhi = Zhonghua ganzangbing zazhi = Chinese journal of hepatology* 2013; **21**(3): 202-6.
394. Li C, Mi K, Wen TF, et al. Risk factors and outcomes of massive red blood cell transfusion following living donor liver transplantation. *Journal of Digestive Diseases* 2012; **13**(3): 161-7.
395. Zhang C, Zhao L, Ma L, et al. Vitamin D status and expression of vitamin D receptor and LL-37 in patients with spontaneous bacterial peritonitis. *Digestive Diseases and Sciences* 2012; **57**(1): 182-8.

396. Chen J, Wang Y, Shen Z, Zhu Z, Song Y, Han R. Early diagnostic value of plasma PCT and BG assay for CRBSI after OLT. *Transplantation Proceedings* 2011; **43**(5): 1777-9.
397. Xu X, Ling Q, Wei Q, et al. An effective model for predicting acute kidney injury after liver transplantation. *Hepatobiliary and Pancreatic Diseases International* 2010; **9**(3): 259-63.
398. Wang NY, Zhang D, Zhao W, Fang GX, Shi YL, Duan MH. Clinical application of an enzyme-linked immunosorbent assay detecting hepatoma-specific γ-glutamyltransferase. *Hepatology Research* 2009; **39**(10): 979-87.
399. Liu XY, Hu JH, Wang HF, Chen JM. [Etiological analysis of 1977 patients with acute liver failure, subacute liver failure and acute-on-chronic liver failure]. *Zhonghua gan zang bing za zhi = Zhonghua ganzangbing zazhi = Chinese journal of hepatology* 2008; **16**(10): 772-5.
400. Wang Y, Liu Y, Han R, et al. Hemostatic variation during perioperative period of orthotopic liver transplantation without venovenous bypass. *Thrombosis Research* 2008; **122**(2): 161-6.
401. Okuno H, Xie ZC, Lu BY, et al. Genotypes of hepatitis C virus in Guangxi province, southern China. *Journal of gastroenterology and hepatology* 1994; **9**(2): 169-71.
402. Wang Y, Okamoto H, Tsuda F, Nagayama R, Tao QM, Mishiro S. Prevalence, genotypes, and an isolate (HC-C2) of hepatitis C virus in Chinese patients with liver disease. *Journal of medical virology* 1993; **40**(3): 254-60.
403. Liu K, Wong VW, Lau K, et al. Prognostic Value of Controlled Attenuation Parameter by Transient Elastography. *Am J Gastroenterol* 2017; **112**(12): 1812-23.
404. Huang CH, Tseng HJ, Amodio P, et al. Hepatic encephalopathy and spontaneous bacterial peritonitis improve cirrhosis outcome prediction: A modified seven-stage model as a clinical alternative to MELD. *Journal of Personalized Medicine* 2020; **10**(4): 1-13.
405. Chen PC, Chen BH, Huang CH, et al. Integrated model for end-stage liver disease maybe superior to some other model for end-stage liver disease-based systems in addition to Child-Turcotte-Pugh and albumin-bilirubin scores in patients with hepatitis B virus-related liver cirrhosis and spontaneous bacterial peritonitis. *European journal of gastroenterology & hepatology* 2019; **31**(10): 1256-63.
406. Kuo YH, Kee KM, Hsu NT, et al. Using AST-platelet ratio index and fibrosis 4 index for detecting chronic hepatitis C in a large-scale community screening. *PloS one* 2019; **14**(10).
407. Sung CM, Chen KF, Lin YF, et al. Predicting Clinical Outcomes of Cirrhosis Patients With Hepatic Encephalopathy From the Fecal Microbiome. *Cellular and Molecular Gastroenterology and Hepatology* 2019; **8**(2): 301-18.e2.
408. Wang HK, Chen CY, Lin NC, et al. Comparison of Two Devices for Intraoperative Portal Venous Flow Measurement in Living-Donor Liver Transplantation: Transit Time Ultrasound and Conventional Doppler Ultrasound. *Transplantation Proceedings* 2018; **50**(4): 1157-9.
409. Wang HY, Chen MJ, Lin CC, et al. Gastric variceal bleeding in the elderly. *International Journal of Gerontology* 2012; **6**(4): 258-61.
410. Xu HW, Lu SN, Hung CH, Chang KC, Hu TH, Wang JH. Liver stiffness measurement in cirrhotic patient --implications of disease activity and treatment efficacy. *The Kaohsiung journal of medical sciences* 2012; **28**(12): 641-8.
411. Tu KH, Jenq CC, Tsai MH, et al. Outcome scoring systems for short-term prognosis in critically ill cirrhotic patients. *Shock* 2011; **36**(5): 445-50.
412. Chou AL, Huang WW, Lin MN, Su CC. Human herpesvirus type 8 in patients with cirrhosis independent of thrombocytopenia. *Journal of clinical pathology* 2010; **63**(3): 254-8.
413. Chou AL, Huang WW, Tsao SM, Li CT, Su CC. Human herpesvirus type 8 in patients with cirrhosis: Correlation with sex, alcoholism, hepatitis B virus, disease severity, and thrombocytopenia. *American Journal of Clinical Pathology* 2008; **130**(2): 231-7.
414. Chang TS, Lo SK, Shyr HY, et al. Hepatitis C virus infection facilitates gallstone formation. *Journal of gastroenterology and hepatology* 2005; **20**(9): 1416-21.
415. Chen WC, Lo GH, Lai KH, Cheng JS, Hsu PI, Lin CK. Development of hepatocellular carcinoma after successful management of esophageal variceal bleeding. *Journal of the Chinese Medical Association : JCMA* 2004; **67**(11): 557-64.
416. Chen CH, Wang JH, Lu SN, et al. Comparison of prevalence for paraumbilical vein patency in patients with viral and alcoholic liver cirrhosis. *American Journal of Gastroenterology* 2002; **97**(9): 2415-8.
417. Yatsuhashi H, Sano H, Hirano T, Shibasaki Y. Real-world hospital mortality of liver cirrhosis inpatients in Japan: a large-scale cohort study using a medical claims database: Prognosis of liver cirrhosis. *Hepatology Research* 2021.
418. Enomoto H, Ueno Y, Hiasa Y, et al. Transition in the etiology of liver cirrhosis in Japan: a nationwide survey. *Journal of Gastroenterology* 2020; **55**(3): 353-62.
419. Sato S, Namisaki T, Furukawa M, et al. Effect of l-carnitine on health-related quality of life in patients with liver cirrhosis. *Biomedical Reports* 2020; **13**(6): 1-9.
420. Sawada Y, Kawaratani H, Kubo T, et al. Effect of furosemide on muscle cramps in patients with liver cirrhosis. *Journal of Gastroenterology and Hepatology (Australia)* 2020; **35**(1): 76-81.

421. Sharshar M, Yagi S, Iida T, et al. Liver transplantation in patients with portal vein thrombosis: A strategic road map throughout management. *Surgery (United States)* 2020; **168**(6): 1160-8.
422. Tamai H, Minamiguchi H, Ida Y, et al. Combination with portosystemic shunt occlusion and antiviral therapy improves prognosis of decompensated cirrhosis. *JGH Open* 2020; **4**(4): 670-6.
423. Kida Y. Positive Response to Tolvaptan Treatment Would Be a Good Prognostic Factor for Cirrhotic Patients with Ascites. *Digestive Diseases* 2019; **37**(3): 239-46.
424. Kido-Nakahara M, Nakahara T, Furusyo N, et al. Pruritus in chronic liver disease: A questionnaire survey on 216 patients. *Acta Dermato-Venereologica* 2019; **99**(2): 220-1.
425. Kojima S, Watanabe N, Koizumi J, et al. Current status of portal vein thrombosis in Japan: Results of a questionnaire survey by the Japan Society for Portal Hypertension. *Hepatology Research* 2018; **48**(4): 244-54.
426. Genda T, Ichida T, Sakisaka S, et al. Survival in patients with Child-Pugh class C cirrhosis: Analysis of the liver transplant registry in Japan. *Hepatology research : the official journal of the Japan Society of Hepatology* 2017; **47**(11): 1155-64.
427. Ishikawa T, Aibe Y, Matsuda T, Iwamoto T, Takami T, Sakaida I. Plasma Glucose Level Is Predictive of Serum Ammonia Level After Retrograde Occlusion of Portosystemic Shunts. *AJR American journal of roentgenology* 2017; **209**(3): W169-w76.
428. Iwasa M, Sugimoto R, Hara N, Mifuji-Moroka R, Takei Y. Hyponatremia observed in hepatic cirrhosis is associated with renal function, use of diuretics and survival. *Japanese Journal of Clinical Pharmacology and Therapeutics* 2016; **47**(1): 21-4.
429. Kanki A, Ito K, Yamamoto A, et al. Evaluation of renal cortical thickness by non-contrastenhanced MR imaging with spatially selective IR pulses: Comparison between cirrhotic and non-cirrhotic patients. *British Journal of Radiology* 2016; **89**(1064).
430. Horie Y, Ebinuma H, Kikuchi M, Nakamoto N, Kanai T. Current status of alcoholic liver diseases in Japan. *Acta Hepatologica Japonica* 2015; **56**(7): 366-8.
431. Toshikuni N, Ozaki K, George J, Tsutsumi M. Serum endocan as a survival predictor for patients with liver cirrhosis. *Canadian journal of gastroenterology & hepatology* 2015; **29**(8): 427-30.
432. Nakamura T, Sata M, Hiroishi K, et al. Contribution of diuretic therapy with human serum albumin to the management of ascites in patients with advanced liver cirrhosis: A prospective cohort study. *Molecular and Clinical Oncology* 2014; **2**(3): 349-55.
433. Nakanishi H, Kurosaki M, Nakanishi K, et al. Impaired brain activity in cirrhotic patients with minimal hepatic encephalopathy: Evaluation by near-infrared spectroscopy. *Hepatology Research* 2014; **44**(3): 319-26.
434. Taguchi K, Yamanaka-Okumura H, Mizuno A, et al. Insulin resistance as early sign of hepatic dysfunction in liver cirrhosis. *The journal of medical investigation : JMI* 2014; **61**(1-2): 180-9.
435. Ohashi N, Tsuji N, Naito Y, et al. Relationship between urinary fractional excretion of sodium and life prognosis in liver cirrhosis patients. *Hepatology Research* 2013; **43**(11): 1156-62.
436. Ozaki K, Matsui O, Gabata T, Kobayashi S, Koda W, Minami T. Confluent hepatic fibrosis in liver cirrhosis: possible relation with middle hepatic venous drainage. *Japanese journal of radiology* 2013; **31**(8): 530-7.
437. Suzuki K, Onodera M, Kakisaka K, et al. Serum free fatty acid concentration as a surrogate marker for serum carnitine status in patients with liver cirrhosis. *Acta Hepatologica Japonica* 2013; **54**(11): 796-7.
438. Takahashi H, Suzuki M, Shigefuku R, et al. Xenon computed tomography can evaluate the improvement of hepatic hemodynamics before and after endoscopic injection sclerotherapy. *Journal of gastroenterology* 2013; **48**(12): 1353-61.
439. Takuma Y, Nouso K, Morimoto Y, et al. Measurement of Spleen Stiffness by Acoustic Radiation Force Impulse Imaging Identifies Cirrhotic Patients With Esophageal Varices. *Gastroenterology* 2013; **144**(1): 92-U195.
440. Tomoda T, Nouso K, Miyahara K, et al. Prognotic impact of serum follistatin in patients with hepatocellular carcinoma. *Journal of Gastroenterology and Hepatology (Australia)* 2013; **28**(8): 1391-6.
441. Yamanaka-Okumura H, Nakamura-Kutsuzawa T, Teramoto A, et al. Non-esterified fatty acid is being validated as a substitute measure for non-protein respiratory quotient in patients with cirrhosis. *e-SPEN Journal* 2013; **8**(3): e90-e4.
442. Yoshida D, Nagao Y, Tomikawa M, et al. Predictive factors for platelet count after laparoscopic splenectomy in cirrhotic patients. *Hepatology international* 2012; **6**(3): 657-61.
443. Michitaka K, Hiraoka A, Kume M, et al. Amino acid imbalance in patients with chronic liver diseases. *Hepatology Research* 2010; **40**(4): 393-8.
444. Horie Y, Yamagishi Y, Kikuchi M, et al. [Influence of habitual drinking and viral hepatitis type C in the progression of liver cirrhosis]. *Nihon Arukoru Yakubutsu Igakkai zasshi = Japanese journal of alcohol studies & drug dependence* 2009; **44**(1): 38-42.
445. Ohashi M, Sugata K, Ihira M, et al. Human herpesvirus 6 infection in adult living related liver transplant recipients. *Liver Transpl* 2008; **14**(1): 100-9.

446. Takeshita E, Matsui H, Shibata N, et al. Earlier recurrence of esophageal varices, following therapy, in patients with primary biliary cirrhosis (PBC) compared with non-PBC patients. *Journal of Gastroenterology* 2004; **39**(11): 1085-9.
447. Kamamura M, Honda H, Inoue H, et al. Study of the causes of higher mortality rates from chronic liver diseases in Tokushima Prefecture. *The journal of medical investigation : JMI* 2002; **49**(3-4): 163-71.
448. Nagata T, Matsumoto A, Uehara Y, et al. Oxygenation abnormalities in normoxemic patients with mild liver cirrhosis. *Internal Medicine* 2002; **41**(6): 435-40.
449. Shibuya A, Ikewaki N. High serum glyceraldehyde-3-phosphate dehydrogenase levels in patients with liver cirrhosis. *Hepatology Research* 2002; **22**(3): 174-9.
450. Ishii M, Gama H, Chida N, et al. Simultaneous measurements of serum alpha-fetoprotein and protein induced by vitamin K absence for detecting hepatocellular carcinoma. South Tohoku District Study Group. *Am J Gastroenterol* 2000.
451. Miyakawa H, Izumi N, Marumo F, Sato C. Roles of alcohol, hepatitis virus infection, and gender in the development of hepatocellular carcinoma in patients with liver cirrhosis. *Alcoholism, clinical and experimental research* 1996; **20**(1 Suppl): 91a-4a.
452. Kaneko S, Unoura M, Takeuchi M, et al. The role of hepatitis C virus in hepatocellular carcinoma in Japan. *Intervirology* 1994; **37**(2): 108-13.
453. Sato Y, Nakata K, Kato Y, et al. Early recognition of hepatocellular carcinoma based on altered profiles of alpha-fetoprotein. *The New England journal of medicine* 1993; **328**(25): 1802-6.
454. Dondog B, Lise M, Dondov O, Baldandorj B, **Franceschi S**. Hepatitis B and C virus infections in hepatocellular carcinoma and cirrhosis in Mongolia. *European journal of cancer prevention : the official journal of the European Cancer Prevention Organisation (ECP)* 2011; **20**(1): 33-9.
455. Oyunsuren T, Sanduijav R, Davaadorj D, Nansalmaa D. Hepatocellular carcinoma and its early detection by AFP testing in Mongolia. *Asian Pacific journal of cancer prevention : APJCP* 2006; **7**(3): 460-2.
456. Jang WY, Chung WJ, Jang BK, et al. Changes in Characteristics of Patients with Liver Cirrhosis Visiting a Tertiary Hospital over 15 Years: a Retrospective Multi-Center Study in Korea. *Journal of Korean Medical Science* 2020; **35**(29).
457. Park HJ, Jang HY, Kim SY, et al. Non-enhanced magnetic resonance imaging as a surveillance tool for hepatocellular carcinoma: Comparison with ultrasound. *Journal of Hepatology* 2020; **72**(4): 718-24.
458. Park JG, Tak WY, Park SY, et al. Effects of branched-chain amino acid (BCAA) supplementation on the progression of advanced liver disease: A korean nationwide, multicenter, prospective, observational, cohort study. *Nutrients* 2020; **12**(5).
459. Kim MN, Kim BK, Kim SU, et al. Longitudinal assessment of alpha-fetoprotein for early detection of hepatocellular carcinoma in patients with cirrhosis. *Scandinavian Journal of Gastroenterology* 2019; **54**(10): 1283-90.
460. Kim TH, Lee HA, Seo YS, et al. Assessment and prediction of acute kidney injury in patients with decompensated cirrhosis with serum cystatin C and urine N-acetyl-β-D-glucosaminidase. *Journal of Gastroenterology and Hepatology (Australia)* 2019; **34**(1): 234-40.
461. Park SH, Joo MS, Kim BH, et al. Clinical characteristics and prevalence of adrenal insufficiency in hemodynamically stable patients with cirrhosis. *Medicine* 2018; **97**(26): e11046.
462. Ahn JM, Kim CH, Um SH, et al. Validation study associating glutaminase promoter variations with hepatic encephalopathy in East Asian populations. *Journal of Gastroenterology and Hepatology (Australia)* 2017; **32**(4): 901-7.
463. Ko YS, Bae JH, Sinn DH, et al. [The Clinical Significance of Serum Alpha-fetoprotein in Diagnosing Hepatocellular Carcinoma in a Health Screening Population]. *The Korean journal of gastroenterology = Taehan Sohwagi Hakhoe chi* 2017; **69**(4): 232-8.
464. Na HY, Kim JH, Choe WH, Kwon SY, Yoo BC. Clinical Features of Spontaneous Bacterial Peritonitis: A 10-year Experience from a Single Center. *The Korean journal of gastroenterology = Taehan Sohwagi Hakhoe chi* 2017; **69**(2): 129-34.
465. Hong YS, Sinn DH, Gwak GY, et al. Characteristics and outcomes of chronic liver disease patients with acute deteriorated liver function by severity of underlying liver disease. *World journal of gastroenterology* 2016; **22**(14): 3785-92.
466. Kang SH, Yim HJ, Kim SY, et al. Proton pump inhibitor therapy is associated with reduction of early bleeDing risk after prophylactic endoscopic variceal band ligation a retrospective cohort study. *Medicine (United States)* 2016; **95**(8).
467. Lee SK, Choi JY, Yeo DM, et al. Risk factors of biliary intervention by imaging after living donor liver transplantation. *World Journal of Gastroenterology* 2016; **22**(7): 2342-8.
468. Lim TS, Kim DY, Han KH, et al. Combined use of AFP, PIVKA-II, and AFP-L3 as tumor markers enhances diagnostic accuracy for hepatocellular carcinoma in cirrhotic patients. *Scand J Gastroenterol* 2016; **51**(3): 344-53.
469. Park Y, Kim SU, Park SY, et al. A novel model to predict esophageal varices in patients with compensated cirrhosis using acoustic radiation force impulse elastography. *PloS one* 2015; **10**(3).

470. Lee S, Park SJ, Cheon JH, et al. Child-pugh score is an independent risk factor for immediate bleeding after colonoscopic polypectomy in liver cirrhosis. *Yonsei Medical Journal* 2014; **55**(5): 1281-8.
471. Seo YS, Park SY, Kim MY, et al. Lack of difference among terlipressin, somatostatin, and octreotide in the control of acute gastroesophageal variceal hemorrhage. *Hepatology* 2014; **60**(3): 954-63.
472. Koo JK, Kim JH, Choi YJ, et al. Predictive value of Refit Model for End-Stage Liver Disease, Refit Model for End-Stage Liver Disease-Na, and pre-existing scoring system for 3-month mortality in Korean patients with cirrhosis. *Journal of Gastroenterology and Hepatology (Australia)* 2013; **28**(7): 1209-16.
473. Choi JH, Kim ER, Min BH, et al. The feasibility and safety of the endoscopic submucosal dissection of superficial gastric neoplastic lesions in patients with compensated liver cirrhosis: A retrospective study. *Gut and Liver* 2012; **6**(1): 58-63.
474. Kim TW, Kim HJ, Chon CU, et al. Is there any vindication for low dose nonselective beta-blocker medication in patients with liver cirrhosis? *Clinical and molecular hepatology* 2012; **18**(2): 203-12.
475. Nam SW. Long-term complementary dietary supplement of vegetable protein may decrease degree of hepatic encephalopathy in patients with decompensated liver cirrhosis. *Research Journal of Medical Sciences* 2012; **6**(3): 113-6.
476. Yu YD, Lee SG, Joh JW, et al. Results of a phase 4 trial of Tacrobell® in liver transplantation patients: A multicenter study in South Korea. *Hepato-Gastroenterology* 2012; **59**(114): 357-63.
477. Kwon JH, Park ST, Kim GD, et al. [The value of serum retinol-binding protein 4 levels for determining disease severity in patients with chronic liver disease]. *The Korean journal of hepatology* 2009; **15**(1): 59-69.
478. Seo YS, Jung ES, An H, et al. Serum cystatin C level is a good prognostic marker in patients with cirrhotic ascites and normal serum creatinine levels. *Liver international : official journal of the International Association for the Study of the Liver* 2009; **29**(10): 1521-7.
479. Kim DJ, Kim HY, Kim SJ, et al. Helicobacter pylori infection and peptic ulcer disease in patients with liver cirrhosis. *Korean Journal of Internal Medicine* 2008; **23**(1): 16-21.
480. Moon DB, Lee SG. Adult-to-adult living donor liver transplantation at the Asan Medical Center. *Yonsei Medical Journal* 2004; **45**(6): 1162-8.
481. Kim YS, Um SH, Ryu HS, et al. The Prognosis of Liver Cirrhosis in Recent Years in Korea. *Journal of Korean Medical Science* 2003; **18**(6): 833-41.
482. Park BC, Han BH, Ahn SY, et al. Prevalence of hepatitis C antibody in patients with chronic liver disease and hepatocellular carcinoma in Korea. *J Viral Hepat* 1995; **2**(4): 195-202.
483. Somé EN, Nana FW, Lompo IT, Maxime D, Sombié R. Cirrhosis: Therapeutic Aspects and Outcome for Hospitalized Patients in Burkina Faso. *Open Journal of Epidemiology* 2021; **11**(02): 152-62.
484. Aubry P, Larouze B, Muhirwa G, Henzel D, Ndabaneze E, Nsabimana JM. [Relationship between chronic liver diseases and hepatitis C in Burundi adults]. *Medecine tropicale : revue du Corps de sante colonial* 1995; **55**(3): 235-7.
485. Kowo MP, Diffo AF, Abena MEN, et al. Oral health status of patients with decompensated liver cirrhosis in two hospitals of Yaoundé Cameroon: A comparative study. *Open Journal of Gastroenterology and Hepatology* 2021.
486. Mudji J, Malala J, Horsmans Y. Seroprevalence of viral hepatitis B and C in two populations: blood donors and patients with suspected hepatic cirrhosis in Kwilu province, Democratic Republic of Congo. *International Journal of Infectious Diseases* 2021; **110**: 222-5.
487. Muhie OA. Causes and Clinical Profiles of Ascites at University of Gondar Hospital, Northwest Ethiopia: Institution-Based Cross-Sectional Study. *Canadian journal of gastroenterology & hepatology* 2019; **2019**: 5958032.
488. Tsega E, Nordenfelt E, Hansson BG. Hepatitis C virus infection and chronic liver disease in Ethiopia where hepatitis B infection is hyperendemic. *Transactions of the Royal Society of Tropical Medicine and Hygiene* 1995; **89**(2): 171-4.
489. Perret JL, Moussavou-Kombila JB, Delaporte E, et al. [HBs Ag and antibodies to hepatitis C virus in complicated chronic liver disease in Gabon. A case control study]. *Gastroenterologie clinique et biologique* 2002; **26**(2): 131-5.
490. Duah A, Agyei-Nkansah A, Osei-Poku F, Duah F, Addo BP. Sociodemographic characteristics, complications requiring hospital admission and causes of in-hospital death in patients with liver cirrhosis admitted at a district hospital in Ghana. *PloS one* 2021; **16**(6 June).
491. Duah A, Nkrumah KN, Tachi K. Oesophageal varices in patients with liver cirrhosis attending a major tertiary hospital in Ghana. *Pan African Medical Journal* 2018; **31**.
492. Ilako FM, McLigeyo SO, Riyat MS, Lule GN, Okoth FA, Kaptich D. The prevalence of hepatitis C virus antibodies in renal patients, blood donors and patients with chronic liver disease in Kenya. *East African medical journal* 1995; **72**(6): 362-4.
493. Dembele M, Maiga I, Minta D, et al. [Study of antigen HBs and antivirus antibodies of hepatitis C during hepatopathies in Mali]. *Bulletin de la Societe de pathologie exotique (1990)* 2004; **97**(3): 161-4.
494. Nwokediuko SC, Osuala PC, Uduma UV, Alaneme AK, Onwuka CC, Mesigo C. Pattern of liver disease admissions in a Nigerian tertiary hospital. *Nigerian Journal of Clinical Practice* 2013; **16**(3): 339-42.

495. Adekanle O, Sunmonu TA, Komolafe MA, Ndububa DA. Cognitive functions in patients with liver cirrhosis: Assessment using community screening interview for dementia. *Annals of African Medicine* 2012; **11**(4): 222-9.
496. Mets T, Smitz J, Ngendahayo P, Sabbe L, Bigilimana I, Ngirabatware B. Hepatitis C virus infection in African patients with liver cirrhosis or primary hepatocellular carcinoma. *Scand J Gastroenterol* 1993; **28**(4): 331-4.
497. Mbaye PS, Renaudineau Y, Diallo A, et al. [Hepatitis C virus and chronic hepatopathies in Dakar: case-control study]. *Medecine tropicale : revue du Corps de sante colonial* 2000; **60**(1): 47-52.
498. Soni PN, Tait DR, Gopaul W, Sathar MA, Simjee AE. Hepatitis C virus infection in chronic liver disease in Natal. *South African medical journal = Suid-Afrikaanse tydskrif vir geneeskunde* 1996; **86**(1): 80-3.
499. Yindom LM, Mendy M, Bodimeade C, et al. KIR content genotypes associate with carriage of hepatitis B surface antigen, e antigen and HBV viral load in Gambians. *PloS one* 2017; **12**(11): e0188307.
500. O'Hara G, Mokaya J, Hau JP, et al. Liver function tests and fibrosis scores in a rural population in Africa: a cross-sectional study to estimate the burden of disease and associated risk factors. *Bmj Open* 2020; **10**(3).
501. Apica BS, Ocama P, Seremba E, Opio KC, Kagimu MM. Decompensated cirrhosis-related admissions in a large urban hospital in Uganda: prevalence, clinical and laboratory features and implications for planning patient management. Afr Health Sci. 2014/06/19 ed; 2013. p. 927-32.
502. Salman A, Sholkamy A, Salman M, et al. Study of early postoperative doppler changes post living donor liver transplantation and their impact on early mortality and small-for-size syndrome: A retrospective study. *International Journal of General Medicine* 2021; **14**: 309-17.
503. Fouad TR, Abdelsameea E, Abdel-Razek W, et al. Upper gastrointestinal bleeding in Egyptian patients with cirrhosis: Post-therapeutic outcome and prognostic indicators. *Journal of Gastroenterology and Hepatology (Australia)* 2019; **34**(9): 1604-10.
504. Hamdy HS, El-Ray A, Salaheldin M, et al. Urinary Neutrophil Gelatinase-Associated Lipocalin in Cirrhotic Patients with Acute Kidney Injury. *Annals of hepatology* 2018; **17**(4): 624-30.
505. El-Amin H, Sabry AMM, Ahmed RE, Makhlouf NA. Types and microbiological spectrum of infections in patients with cirrhosis: A single-centre experience in Upper Egypt. *Arab Journal of Gastroenterology* 2017; **18**(3): 159-64.
506. Kandil MA, Abouelenain KM, Alsebaey A, et al. Impact of terlipressin infusion during and after live donor liver transplantation on incidence of acute kidney injury and neutrophil gelatinase-associated lipocalin serum levels: A randomized controlled trial. *Clinical transplantation* 2017; **31**(8).
507. Shaker MK, Abdel Fattah HI, Sabbour GS, et al. Annexin A2 as a biomarker for hepatocellular carcinoma in Egyptian patients. *World journal of hepatology* 2017; **9**(9): 469-76.
508. Emam M, Ibrahim A, Galal S, Darwish E. Study of frequency of spontaneous bacterial empyema in cirrhotic patients with hepatic hydrothorax. *Journal of Gastroenterology and Hepatology Research* 2015; **4**(4): 1569-72.
509. El-Mougy F, Youssef M, Omran D, et al. Aberrant p16INK4A methylation: Relation to viral related chronic liver disease and hepatocellular carcinoma. *South Asian Journal of Cancer* 2014; **3**(1): 1-4.
510. Fawzy A, Attia H, Khalaf FA, et al. Heat Shock Protein-70 and-27 Expressions as Parameters of Early Diagnosis and Disease Progression in Hepatocellular Carcinoma. *Life Sci J* 2013; **10**(1): 262-8.
511. Metwaly HA, Al-Gayyar MMH, Eletreby S, Ebrahim MA, El-Shishtawy MM. Relevance of serum levels of interleukin-6 and syndecan-1 in patients with hepatocellular carcinoma. *Scientia Pharmaceutica* 2012; **80**(1): 179-88.
512. Hafez MM, Shaarawy SM, Hassan AA, Salim RF, El Salam FMA, Ali AE. Prevalence of transfusion transmitted virus (TTV) genotypes among HCC patients in Qaluobia governorate. *Virology Journal* 2007; **4**.
513. Angelico M, Renganathan E, Gandin C, et al. Chronic liver disease in the Alexandria governorate, Egypt: contribution of schistosomiasis and hepatitis virus infections. *Journal of hepatology* 1997; **26**(2): 236-43.
514. Darwish MA, Amer AF, El-Moeity AA, Darwish NM. Association of hepatitis C virus with liver cirrhosis and hepatocellular carcinoma compared with hepatitis B virus in Egyptian patients. *The Journal of the Egyptian Public Health Association* 1997; **72**(5-6): 569-89.
515. Waked IA, Saleh SM, Moustafa MS, Raouf AA, Thomas DL, Strickland GT. High prevalence of hepatitis C in Egyptian patients with chronic liver disease. *Gut* 1995; **37**(1): 105-7.
516. Lahbabi M, Elyousfi M, Aqodad N, et al. Esophageal variceal ligation for hemostasis of acute variceal bleeding: efficacy and safety. *Pan Afr Med J* 2013; **14**: 95.
517. Elfaki AMH. Aetiology, complications, and preventive measures of liver cirrhosis; Elobeid Hospital; West Sudan. *Sudan JMS* 2008.
518. Elloumi H, Sabbah M, Bibani N, et al. Effect of low dose albumin administration in spontaneous bacterial peritonitis on renal function and survival. *Arab Journal of Gastroenterology* 2019; **20**(4): 205-8.
519. Said Y, Dabbabi A, Salem M, et al. Abdominal surgery in cirrhotic patients: Morbidity and mortality. *Journal Africain d'Hepato-Gastroenterologie* 2011; **5**(1): 19-22.
520. Triki H. [Epidemiology of hepatitis B virus, hepatitis C virus and Delta virus in the general population and in liver cirrhosis in Tunisia]. *Archives de l'Institut Pasteur de Tunis* 1994; **71**(3-4): 403-6.
